# Safety of oral JAK inhibitors in treating alopecia areata: a systematic review and network meta-analysis

**DOI:** 10.3389/fphar.2025.1576553

**Published:** 2025-08-11

**Authors:** Ziyu Qi, Yan Li

**Affiliations:** Department of Dermatology, Affiliated Hospital of Inner Mongolia Medical University, Hohhot, Inner Mongolia, China

**Keywords:** alopecia areata (AA), Janus kinase (JAK) inhibitors, adverse events, systematic review, network meta-analysis

## Abstract

**Objective:**

To assess the safety profile of oral Janus kinase (JAK) inhibitors for treating alopecia areata (AA) among adults.

**Methods:**

A comprehensive search was conducted up to July 2025 across PubMed, Cochrane Library, Embase, and Web of Science for randomized controlled trials (RCTs) on oral JAK inhibitors in managing AA. Two qualified investigators conducted a separate review of the studies, evaluated their quality, and gathered pertinent information. R was utilized for all statistical evaluations, while the “GeMTC” package facilitated the Bayesian network meta-analysis.

**Results:**

In this analysis, 12 studies encompassing 3,840 individuals were examined. The findings from the network meta-analysis suggested that oral JAK inhibitors were generally safe for managing AA. Nonetheless, Baricitinib was linked to an increased likelihood of developing acne (RR [95% CrI] = 4.66 [2.00, 13.44]), urinary tract infections (RR [95% CrI] = 2.72 [1.14, 8.44]), and hyperlipidemia (RR [95% CrI] = 1.77 [1.44, 2.20]). Deuruxolitinib was tied to an increased probability of acne occurrence (RR [95% CrI] = 2.74 [1.58, 5.29]) and elevated creatine phosphokinase (CPK) levels (RR [95% CrI] = 1.98 [1.11, 3.93]). Similarly, ritlecitinib was connected to a greater likelihood of elevated CPK levels (RR [95% CrI] = 2.31 [1.01, 6.70]). The experimental data, which included different dose groups, were further analyzed through subgroup analysis. The results indicated that Baricitinib and Deuruxolitinib exhibited dose-dependent differences in the occurrence of acne and elevated CPK levels.

**Conclusion:**

Oral JAK inhibitors exhibit a favorable safety profile for the treatment of AA. However, baricitinib is more likely to cause acne and infections as opposed to other agents.

## Introduction

Alopecia areata (AA) is a long-lasting autoimmune condition that specifically targets hair follicles, leading to hair loss without scarring on the scalp, face, body, and limbs. Based on disease progression, it can be categorized into three stages: active, stable, and regrowth phases (Dainichi et al., 2024). Around 2% of the worldwide population is affected by AA, and there is no notable difference in the prevalence between genders. Although spontaneous remission can occur, the condition often recurs, significantly impacting patients’ quality of life and overall physical and mental health (Craiglow et al., 2024). Compared to the general population, individuals with AA are more prone to depression and anxiety (Vañó-Galván et al., 2024; Nakamura et al., 2024). While the exact pathogenesis of AA remains unclear, evidence points to the significant involvement of the JAK-STAT signaling pathway in the onset of illnesses. Engaging the JAK-STAT pathway amplifies the expression of genes tied to pro-inflammatory cytokine generation and fosters the activation of CD8^+^ T cells, which specifically attack hair follicles during the anagen stage of the hair growth cycle. This mechanism underpins the therapeutic rationale for using JAK inhibitors to block inflammation triggered by receptor activation (Xing et al., 2014; O'Shea and Plenge, 2012). Among JAK inhibitors, baricitinib was the first drug sanctioned by the U.S. FDA and the European Union for addressing severe AA. Clinical trials have indicated that alternative JAK inhibitors like deuruxolitinib and brepocitinib may be effective for treating AA, but they have not yet received regulatory approval for clinical use. Oral administration is the most common route for JAK inhibitors, which have demonstrated therapeutic efficacy in AA. However, their use has also been associated with adverse effects. While several studies have reviewed the unfavorable consequences of using JAK inhibitors (Guan et al., 2024; Papierzewska et al., 2023), few have directly compared the safety of various JAK inhibitors.The variations in safety among these drugs remain unclear. This study primarily focused on safety assessment, thoroughly exploring various safety concerns while incorporating a greater number of high-quality studies. As a result, the findings provide more comprehensive insights into the safety considerations of JAK inhibitors in the treatment of AA.

A network meta-analysis (NMA) serves as a powerful approach to evaluating the impact of various treatment strategies on a specific disease. Its primary advantage lies in its ability to use indirect comparisons to evaluate the comparative effectiveness and safety of therapies that have not been directly compared in clinical studies, enabling comprehensive cross-sectional comparisons of various interventions. In this study, a systematic review combined with NMA was conducted to gather evidence and assess the safety profile of JAK inhibitors in managing AA.

## Materials and methods

The investigation adhered to the principles set forth in the PRISMA Extension Statement designed for systematic reviews and network meta-analyses (Hutton et al., 2015). (Supplementary Table S1) The research was pre-registered with PROSPERO (registration number: CRD42024489236).

### Strategy for conducting literature searches

An extensive investigation was carried out across PubMed, Cochrane Library, Embase, and Web of Science for articles published up to July 2025. The main search terms included “AA” and “JAK inhibitor.” The comprehensive strategy can be found in Appendices (Supplementary Table S2). There were no limitations on language during the search process.

### Literature screening

Two trained researchers independently screened the literature and extracted data based on predefined inclusion and exclusion criteria, with cross-verification of results. In cases of disagreement, a third researcher provided adjudication, and the final screening results were summarized. Inclusion criteria (Dainichi et al., 2024) Research design: Randomized controlled trials (RCTs) (double-blind or single-blind) (Craiglow et al., 2024); The group of individuals studied consisted of patients aged ≥18 years with a confirmed diagnosis of AA (due to significant physiological differences between children and adults, and among children of varying ages, it is inappropriate to analyze pediatric data alongside adult data. Therefore, this study only included adult patients) (Vañó-Galván et al., 2024); Interventions: The experimental group received oral JAK inhibitors, while the control group received either placebo or other medications (Nakamura et al., 2024); Outcome indicator: The number of occurrences of different types of adverse events (adverse events, AEs). Exclusion criteria (Dainichi et al., 2024) Studies where outcome data were unavailable or unclear (Craiglow et al., 2024); Publications such as reviews, case reports, and conference abstracts.

### Evaluation of potential bias

Two qualified researchers evaluated the quality of the RCTs utilizing the Cochrane Risk of Bias Assessment Tool. This tool evaluates seven domains: allocation concealment, blinding of participants and trial personnel, blinding of outcome assessors, completeness of outcome data, selective outcome reporting, and other potential sources of bias. Each domain was rated as “low risk of bias,” “high risk of bias,” or “uncertain risk of bias”.

### Evaluation of publication bias

Funnel plots were generated using the netmeta package in R to assess publication bias across the meta-analysis.

### Evaluation of credibility

The credibility of the included studies was evaluated using CINeMA, with the results visualized for clarity.

### Information retrieval

The tasks of literature screening and data extraction were executed independently by two researchers who had received training in accordance with the predetermined criteria for inclusion and exclusion. To resolve the discrepancies, a discussion was held with an additional researcher. Data were extracted using a custom-designed electronic spreadsheet, including the following details: first author, study design, sample size, sex distribution, mean age, and intervention measures for the experimental group. The primary outcome of interest was the number of occurrences of various adverse events.

### Statistical examination

R (version 4.3.2) was used in conjunction with the “GeMTC” package (version 1.0-2) for Bayesian meta-analysis. Adverse events (AEs) following drug treatment were treated as dichotomous data, and risk ratios (RRs) were utilized to convey the effect sizes, with their respective 95% credible intervals (95% CrIs). The convergence quality of NMA was evaluated using the potential scale reduction factor (PSRF), with PSRF = 1 indicating satisfactory convergence. If the network included a closed loop, an inconsistency test was performed using the “mtc.nodesplit” function. Heterogeneity was assessed using the “mtc.anohe” function, and I^2^ statistics was utilized to assess the level of inconsistency in various studies. An I^2^ < 50% was interpreted as low heterogeneity, whereas I^2^ ≥ 50% indicated significant heterogeneity. If the source of heterogeneity cannot be identified, NMA was abandoned, and only descriptive analysis would be performed. Treatment rankings were presented in three formats: probability ranking plots, surface under the cumulative ranking (SUCRA) values (where lower values indicated a higher likelihood of adverse events following drug treatment), ranking tables, and forest plots displaying relative effects.

## Results

### Literature acquisition

**FIGURE 1:**
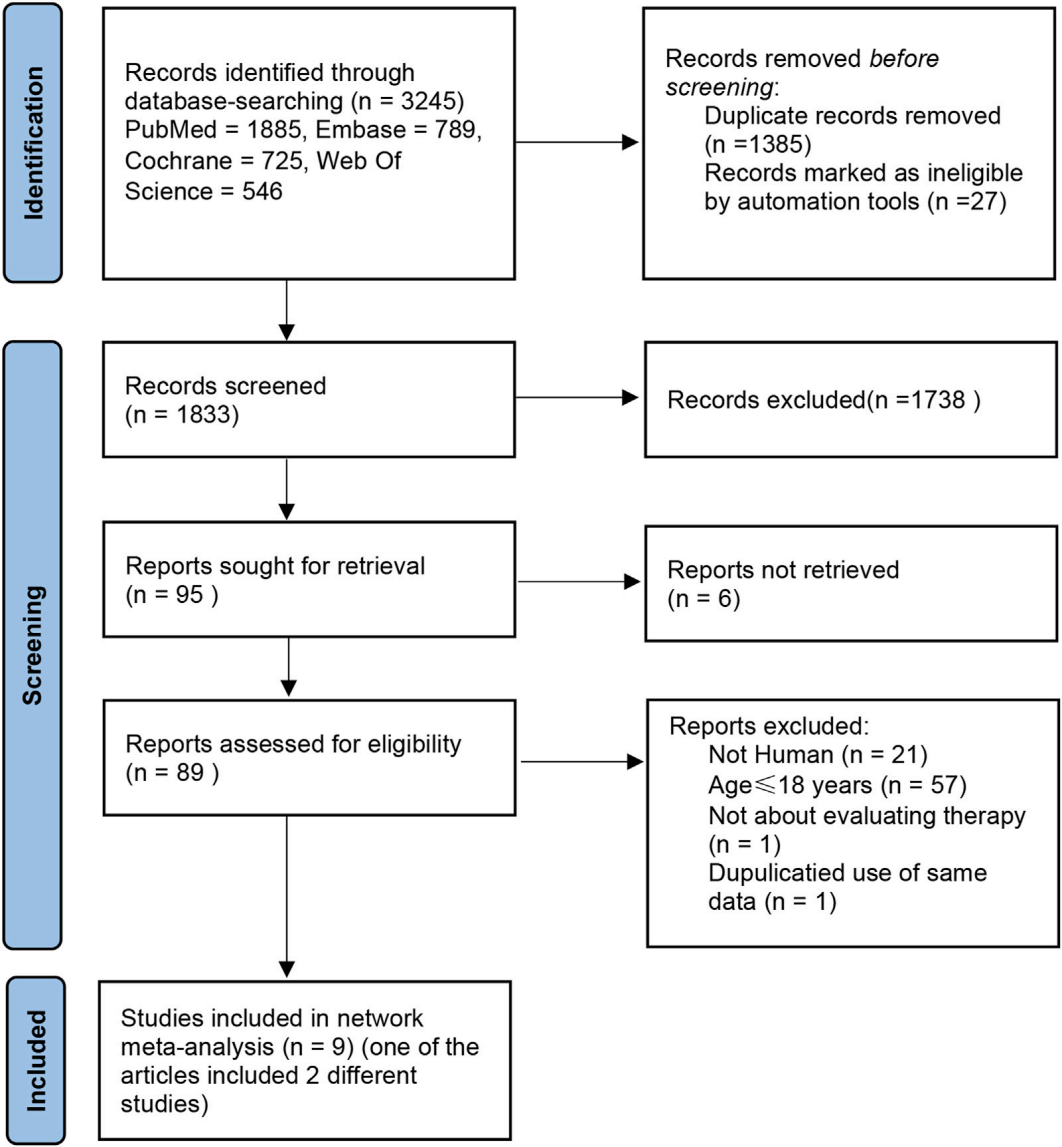
Search for studies for systematic reviews and meta-analyses.

A cumulative count of 3,245 publications was obtained from databases including PubMed, Cochrane Library, Embase, and Web of Science, with a timeframe from database inception to December 2024. After being imported into the literature management software and de-duplicated, 1,833 entries remained. By eliminating articles that were not pertinent based on their titles and abstracts, we identified 89 relevant studies. Upon reviewing the full texts, multiple articles were excluded for the following reasons: 57 articles included participants aged <18 years; 21 articles involved animal studies; 1 article was unrelated to treatment evaluation; and 1 article reused data. Finally, 10 articles were included (Paracha et al., 2024; King et al., 2024; King et al., 2023; King et al., 2021a; Almutairi et al., 2019; King et al., 2022a; King et al., 2021b; Anderson et al., 2024; Zhou et al., 2023; King et al., 2022b). Additionally, 1 article contained two separate RCTs (King et al., 2022b) and 1 unpublished RCT (ClinicalTrials.gov, 2023) (NCT: 04797650). Therefore, Ultimately, 12 RCTs were included in the analysis (Figure 1).

### Risk of bias information

**FIGURE 2:**
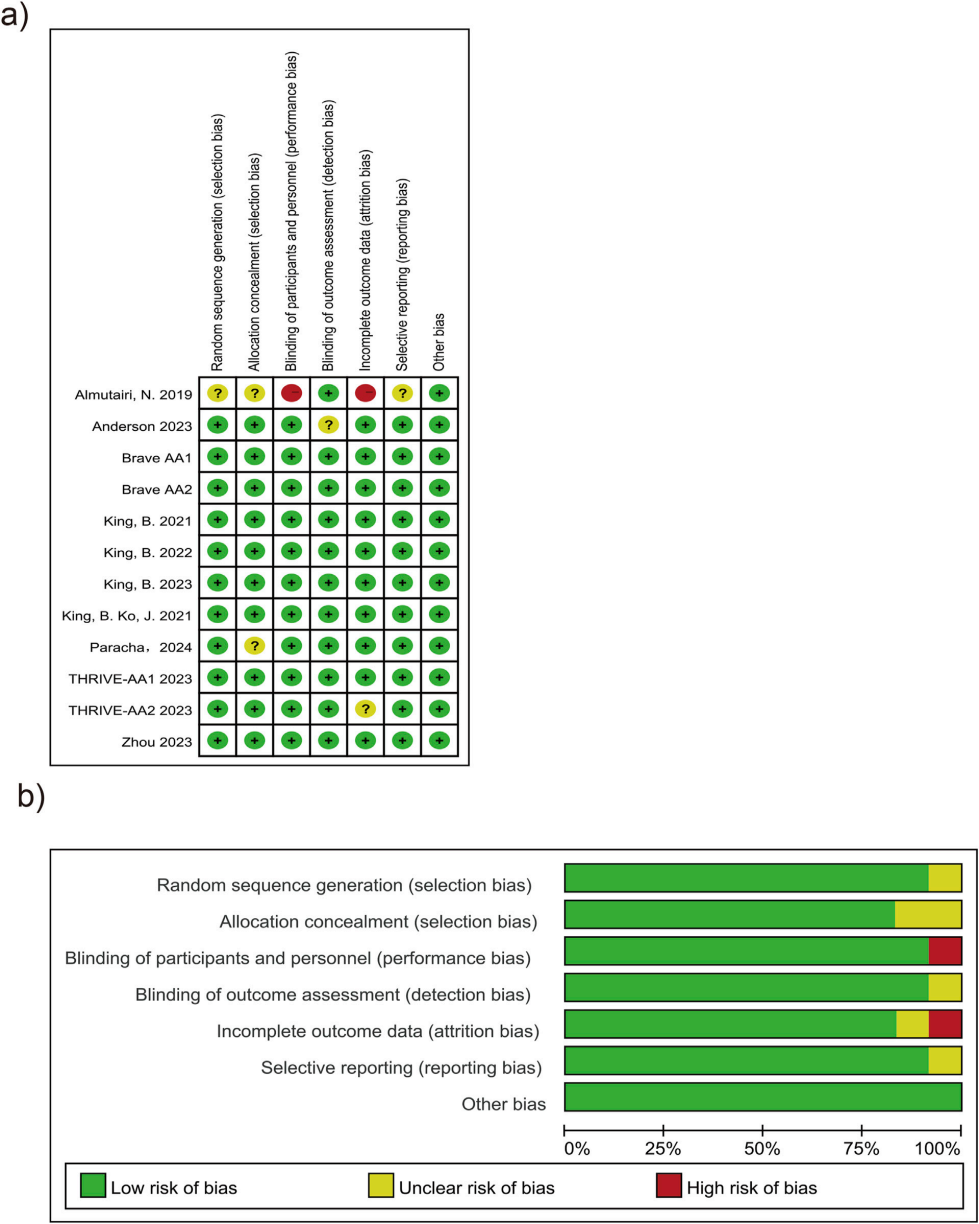
Risk of bias graph and summary. **(a)** Risk of bias graph. **(b)** Risk of bias summary.

A total of 10 articles (Paracha et al., 2024; King et al., 2024; King et al., 2023; King et al., 2021a; Almutairi et al., 2019; King et al., 2022a; King et al., 2021b; Anderson et al., 2024; Zhou et al., 2023; King et al., 2022b) and one unpublished RCT (NCT: 04797650) were included in this study, totaling 12 RCTs. The risk of bias mainly originated from two studies: (King et al., 2023; Almutairi et al., 2019) (Figure 2). In the article by Almutairi et al. (2019), the method of randomization, allocation concealment, and reasons for loss to follow-up were not clearly specified. Additionally, there was no indication of protocol registration (as detailed in Supplementary Figures S1–S3). Publication bias was assessed using funnel plots. The scatter points were approximately inverted funnel-shaped and symmetrically distributed around the effect size mean (vertical line), with no directional bias. The symmetry test yielded a P-value >0.05, indicating no significant statistical difference (Supplementary Figure S4).

### Basic information of the studies

This research involved a cumulative count of 3,840 individuals (Table 1). Most included patients were aged between 30 and 40 years, and the trials featured participants with an average age no younger than 12 years. The patient population can be considered relatively homogeneous. The types and dosages of included interventions were as follows: the control group received a placebo or other oral medications, while the experimental group was treated with oral JAK inhibitors. The following JAK inhibitors taken orally were explored: baricitinib, brepocitinib, ritlecitinib, ruxolitinib, tofacitinib, deuruxolitinib, and ivarmacitinib. The experimental duration ranged from 24 weeks to 36 weeks, with the most common duration being 24 weeks. Baseline SALT scores were typically centered around 80–90 points.

**TABLE 1:** Characteristics of included studies.

Study	Intervention	Treatment duration	Sample size	Age	sex (F, M)	SALT
Zhou et al. (2023)	Ivarmacitinib, 2/4/8mg, qd	24 weeks	70	35.76, 12.17	40, 30	63.74, 21.71
Zhou et al. (2023)	Placebo	24 weeks	24	34.70, 9.40	12, 12	61.30, 19.70
King et al. (2021a)	Baricitinib, 1/2/4 mg, qd	36 weeks	82	41.13, 13.35	66, 16	86.30, 18.11
King et al. (2021b)	placebo	36 weeks	28	40.50, 14.20	16, 12	90.00, 15.70
King et al. (2021a)	Brepocitinib,60mg, qd, 4weeks, then 30mg, qd, 20 weeks	24 weeks	47	34.00, 11.00	32, 15	86.40, 18.10
King et al. (2021b)	Ritlecitinib, 200 mg, qd, 4weeks, then 50 mg, qd, 20 weeks	24 weeks	48	37.00, 13.00	37, 11	89.40, 15.80
King et al. (2021a)	Placebo	24 weeks	47	38.00, 14.00	29, 18	88.40, 18.10
King et al. (2022a)	Deuruxolitinib, 4/8/12mg, bid	24 weeks	105	36.31, 12.60	29, 15	87.90, 17.41
King et al. (2022b)	Placebo	24 weeks	44	37.80, 13.50	76, 29	86.80, 18.39
Almutairi et al. (2019)	Ruxolitinib, 20 mg, bid	6 months	38	35.50, 13.80	21, 17	99.80, 13.63
Almutairi et al. (2019)	Tofacitinib, 5 mg, bid	6 months	37	47.40, 16.10	22, 15	99.60, 14.91
Brave-AA1 2022	Baricitinib, 2/4mg, qd	36 weeks	184	36.97, 13.12	109, 75	86.10, 18.01
Brave-AA1 2022	Placebo	36 weeks	189	37.40, 12.90	109, 80	84.70, 17.80
Brave-AA2 2022	Baricitinib, 2/4mg, qd	36 weeks	390	38.40, 12.81	247, 143	85.12, 18.08
Brave-AA2 2022	Placebo	36 weeks	156	37.10, 12.40	98, 58	85.00, 17.80
Anderson 2023	Ritlecitinib	9 months	36	35.10, 9.60	25, 10	59.60, 30.30
Anderson 2023	Placebo	9 months	35	34.20, 9.00	25, 11	56.90, 27.60
THRIVE-AA1 2023	Deuruxolitinib, 8/12mg, bid	24 weeks	566	36.62, 11.74	348, 218	85.39, 18.36
THRIVE-AA1 2023	Placebo	24 weeks	140	38.50, 11.75	89, 51	88.10, 15.10
Paracha et al. (2024)	Tofacitinib, 5 mg, bid	6 months	30	NA	NA	91.02, 10.21
PParacha et al. (2024)	Azathioprine, 2 mg/kg, qd	6 months	34	NA	NA	91.02, 10.63
THRIVE-AA2, 2023	Deuruxolitinib, 8/12mg, bid	24 weeks	387	38.83, 12.50	261, 126	NA
THRIVE-AA2, 2023	Placebo	24 weeks	130	39.70, 12.49	88, 42	NA

Abbreviations: Scores on the Severity of Alopecia Tool (SALT) range from 0 to 100, with 0 representing no scalp hair loss and 100 complete scalp hair loss; Plus–minus values are means ± SD; sex (F, M), F means the number of female, M means the number of male.

### Statistical analysis results

**FIGURE 3:**
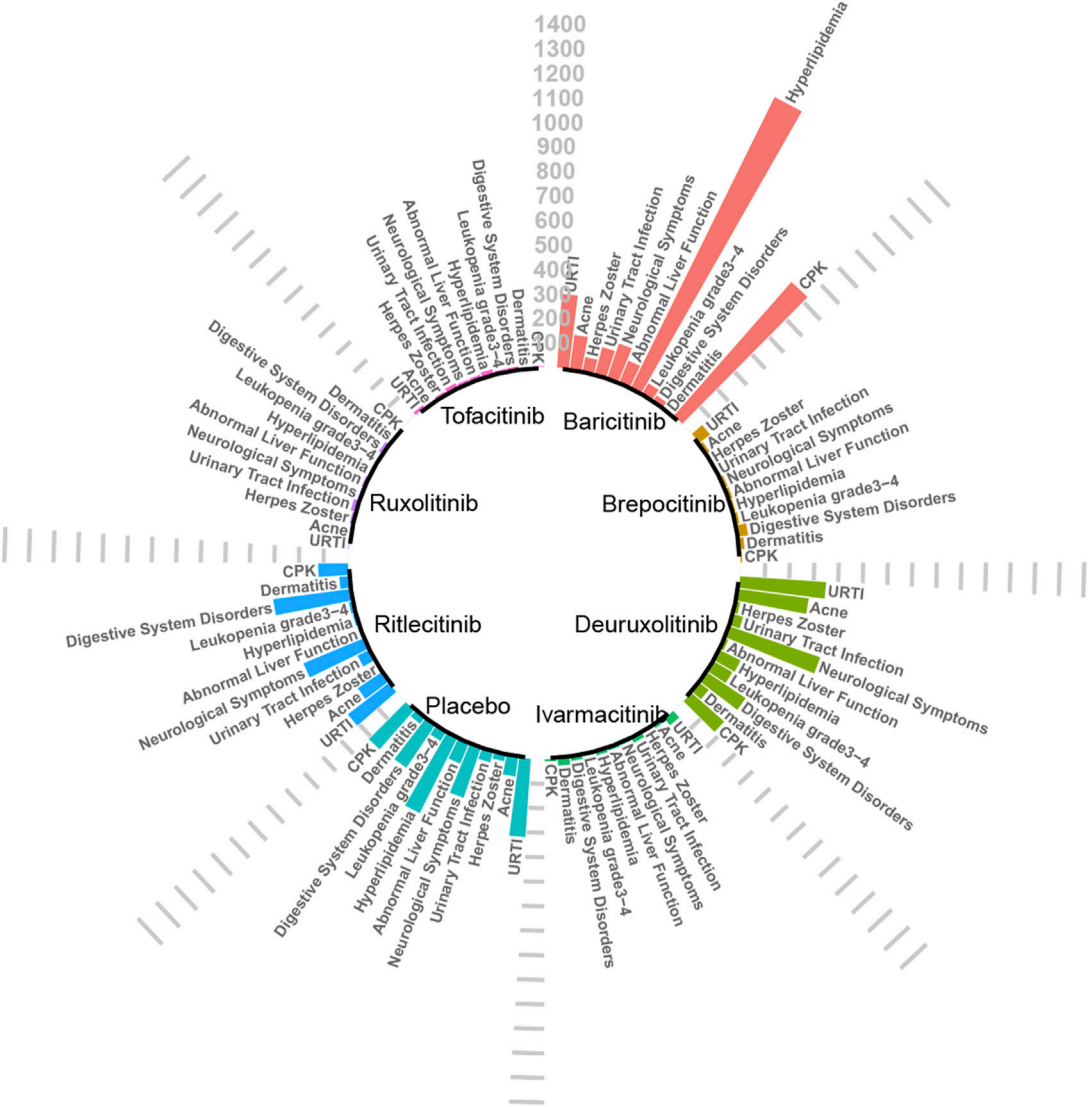
Absolute incidence of adverse reactions caused by JAK inhibitors (per 10,000 people).

**FIGURE 4:**
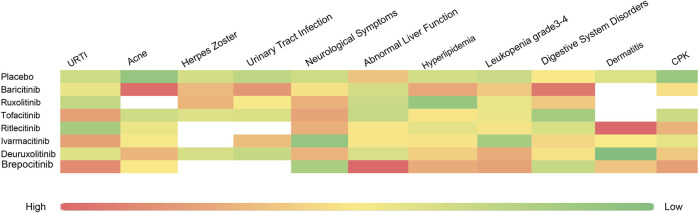
Heat map of SUCRA values for various types of adverse reactions.

Upon analysis, adverse reactions were categorized into two main aspects: the adverse events actually observed, and the abnormalities in laboratory test indices. The incidence rates of each type of adverse reaction are detailed in Figures 3, 4.

#### Upper respiratory tract infections

**FIGURE 5:**
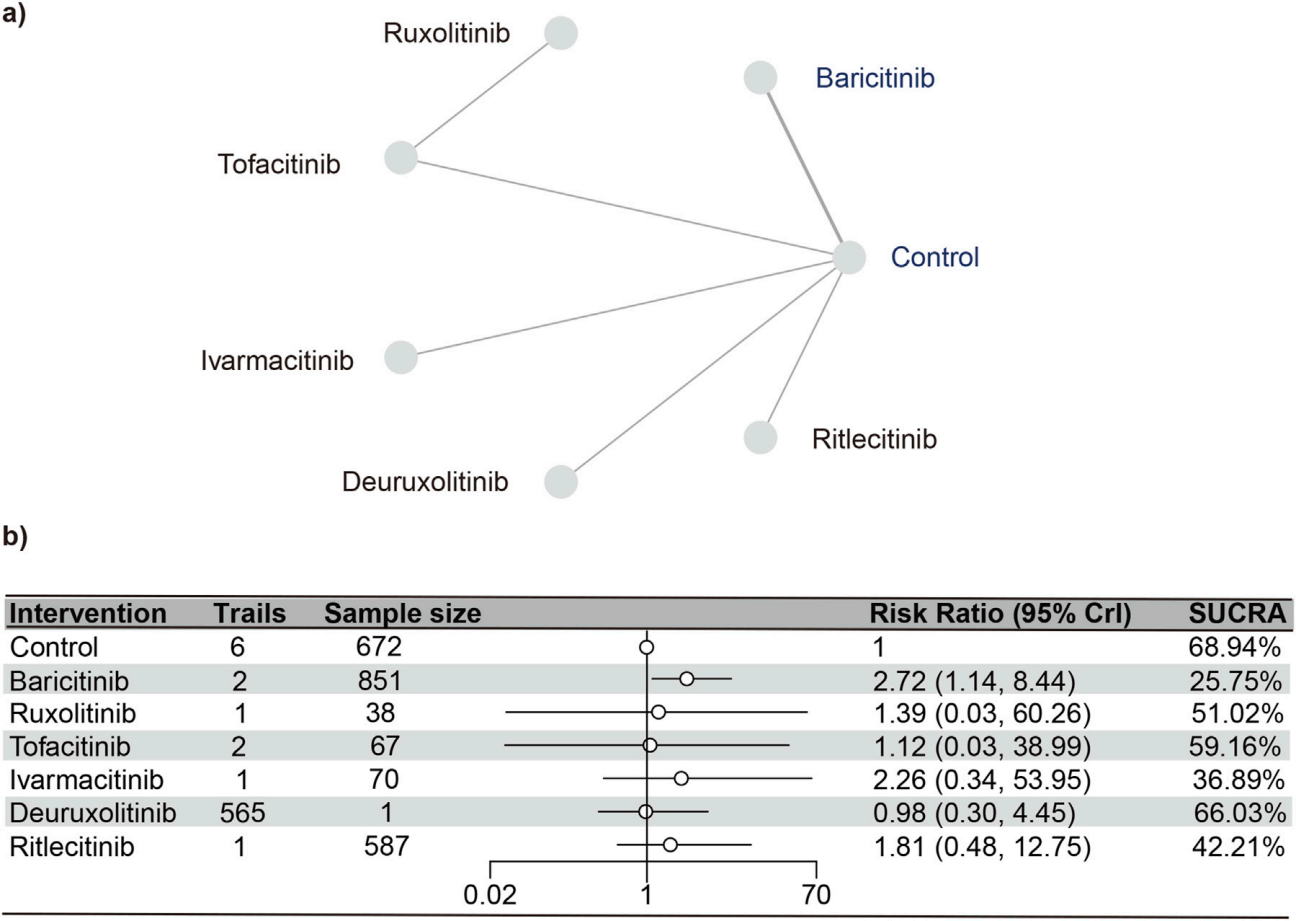
Netplot and forestplot of patients suffering from URTI. **(a)** Netplot of patients suffering from Urinary tract infection. **(b)** Forestplot of patients suffering from Ueinary tract infection.

A total of 12 RCTs (Paracha et al., 2024; King et al., 2024; King et al., 2023; King et al., 2021a; Almutairi et al., 2019; King et al., 2022a; King et al., 2021b; Anderson et al., 2024; Zhou et al., 2023; King et al., 2022b; ClinicalTrials.gov, 2023) involving 3,840 patients were included in the analyses, encompassing seven interventions. The NMA’s PSRF score of 1 demonstrated that convergence was adequate. The results of the network meta-analysis were consistent with the assumptions of homogeneity and consistency, as confirmed by the node-splitting inconsistency test (Supplementary Figures S4, S5). Brepocitinib (RR [95% CrI] = 1.16 [1.02, 2.90]) indicated a marked disparity in comparison to the control group (Figure 5). Additionally, brepocitinib was found to be significantly more likely than ritlecitinib (RR [95% CrI] = 1.98 [1.16, 3.37]) to cause upper respiratory tract infections, as reflected in the league table (Supplementary Table S3). The SUCRA values (Supplementary Figure S6) unveiled similar patterns identified in the forest plot (Figure 3) and the league table (Supplementary Table S3): brepocitinib (22.00%), tofacitinib (28.44%), ivarmacitinib (28.92%), baricitinib (54.98%), deuruxolitinib (57.83%), placebo (63.55%), ruxolitinib (67.61%), ritlecitinib (76.67%).

#### Urinary tract infections

**FIGURE 6:**
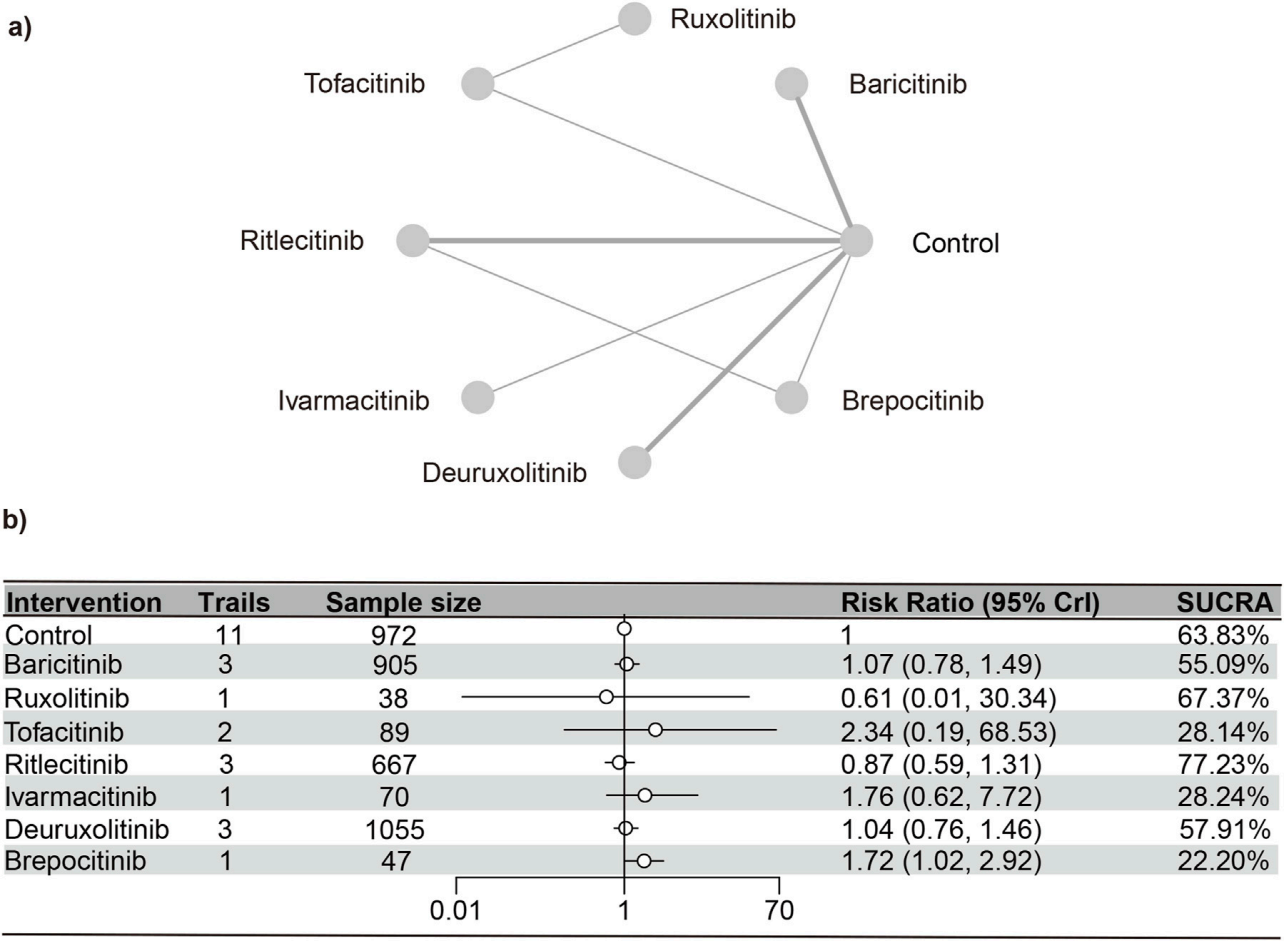
Netplot and forestplot of patients suffering from urinary tract infection. **(a)** Netplot of patients suffering from URTI. **(b)** Forestplot of patients suffering from URTI.

Seven studies (Paracha et al., 2024; King et al., 2024; King et al., 2023; Almutairi et al., 2019; Zhou et al., 2023; King et al., 2022b; ClinicalTrials.gov, 2023) reported urinary tract infections, encompassing 2,850 subjects and six interventions. The PSRF value for NMA was 1, indicating satisfactory convergence. Brepocitinib (RR [95% CrI] = 2.72 [1.14, 8.44]) demonstrated a notable distinction in relation to the control group (Figure 6). Heterogeneity analysis and inconsistency testing using the node-splitting method showed that the NMA satisfied both the homogeneity and consistency assumptions (as detailed in Annex Supplementary Figure S7). Additionally, ruxolitinib, tofacitinib, ivarmacitinib, deuruxolitinib, and ritlecitinib did not differ significantly from that of the control group in the cases of urinary tract infections. The SUCRA values (Supplementary Figure S8) showed patterns that aligned with those observed in the forest plot (Figure 6) and the league table (Supplementary Table S4). The SUCRA values are as follows: baricitinib (25.75%), ivarmacitinib (36.90%), ritlecitinib (42.21%), ruxolitinib (51.01%), tofacitinib (59.16%), deuruxolitinib (66.03%), placebo (68.94%).

#### Acne

**FIGURE 7:**
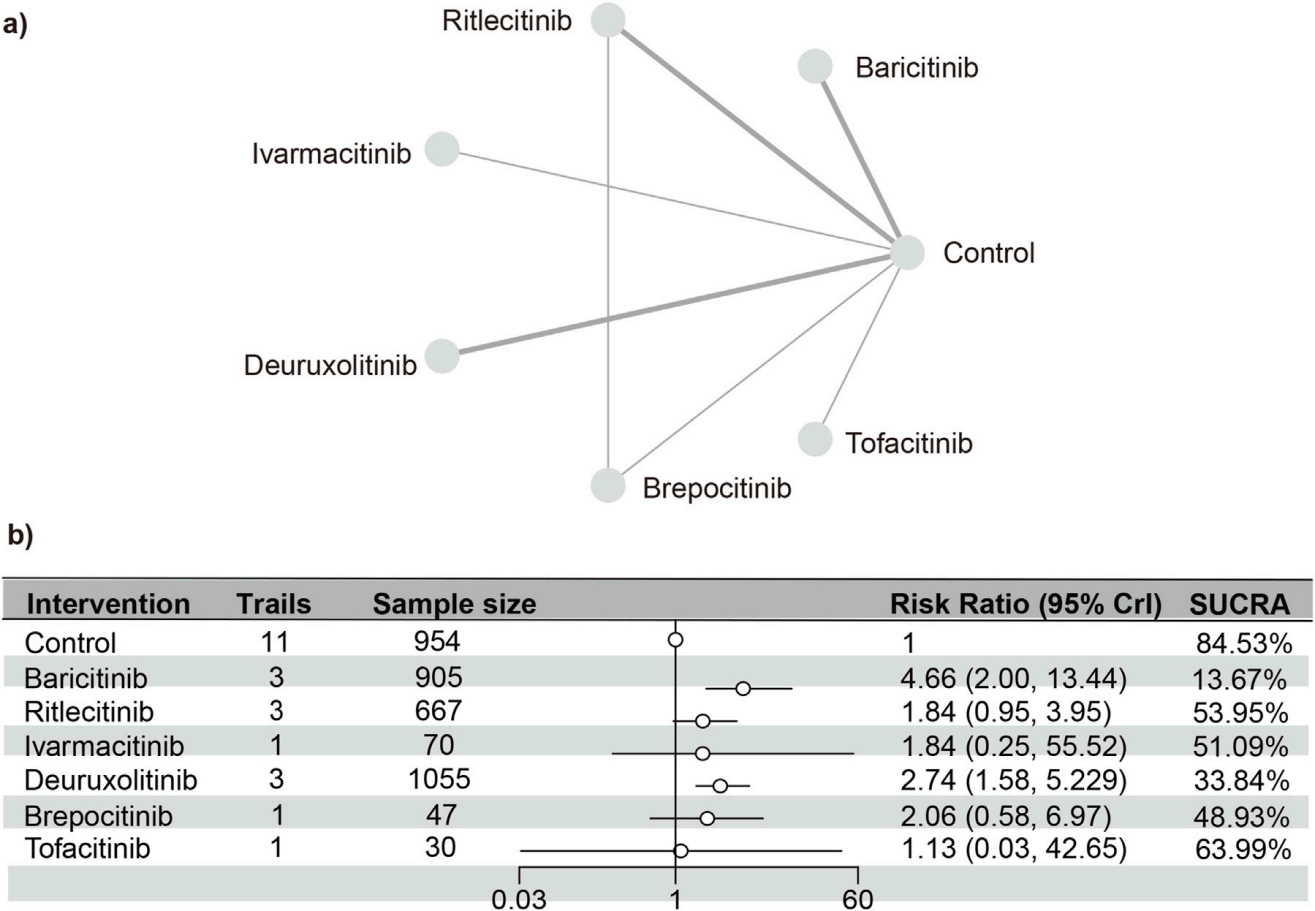
Netplot and forestplot of patients suffering from acne. **(a)** Netplot of patients suffering from acne. **(b)** Forestplot of patients suffering from acne.

Eleven studies (Paracha et al., 2024; King et al., 2024; King et al., 2023; King et al., 2021a; King et al., 2022a; King et al., 2021b; Anderson et al., 2024; Zhou et al., 2023; King et al., 2022b; ClinicalTrials.gov, 2023)were included in the analysis, representing a total of 3,728 patients and six interventions. The PSRF value for NMA was 1, indicating satisfactory convergence. According to heterogeneity analysis and inconsistency testing using the node-splitting method, NMA met the assumptions of homogeneity and consistency (Supplementary Figure S9). Baricitinib (RR [95% CrI] = 4.66 [2.00, 13.44]) and deuruxolitinib (RR [95% CrI] = 2.74 [1.58, 5.29]) significantly increased the number of individuals developing acne as opposed to the control group. Conversely, no significant statistical disparities were found in the incidence of serious adverse reactions with tofacitinib, ritlecitinib, ivarmacitinib, and brepocitinib compared to the control group. The SUCRA values (Supplementary Figure S10) demonstrated trends that were in agreement with the forest plot (depicted in Figure 7) and the league table (Supplementary Table S5): baricitinib (13.67%), deuruxolitinib (33.84%), brepocitinib (48.93%), ivarmacitinib (51.09%), ritlecitinib (53.95%), tofacitinib (63.99%), placebo (84.53%).

#### Remaining adverse reactions

In the incidence of neurologic adverse reactions, gastrointestinal adverse reactions, herpes zoster, and dermatitis, no substantial difference was noted between JAK inhibitors and the control group. Further details are provided in Appendices (depicted in Supplementary Figures S11–22; Supplementary Tables S6–9).

### Laboratory indicators

#### Hyperlipidemia

**FIGURE 8:**
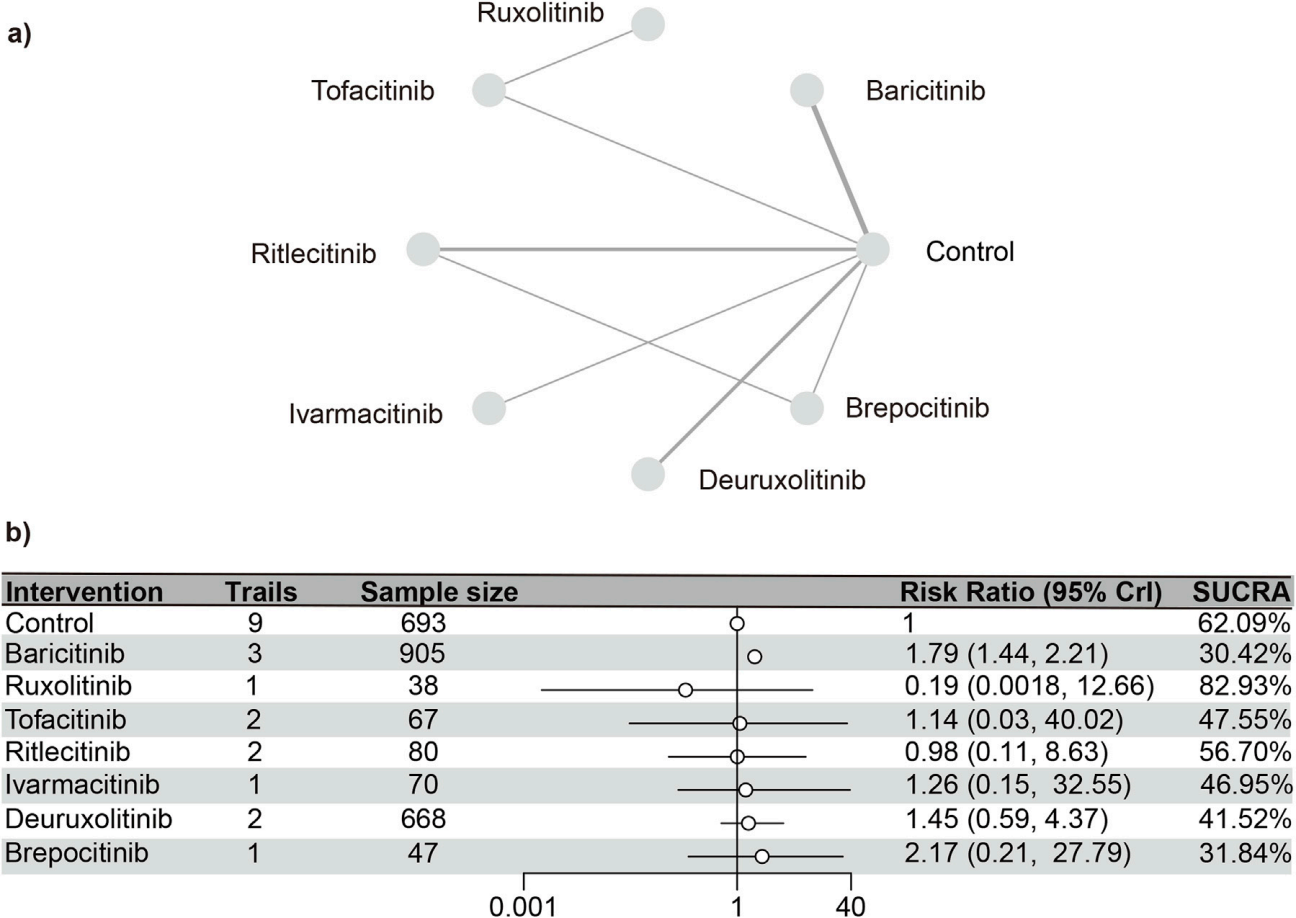
Netplot and forestplot of patients suffering from hyperlipidemia. **(a)** Netplot of patients suffering from Hyperlipidemia. **(b)** Forestplot of patients suffering from Hyperlipidemia.

A total of 10 studies (Paracha et al., 2024; King et al., 2024; King et al., 2021a; Almutairi et al., 2019; King et al., 2022a; King et al., 2021b; Anderson et al., 2024; Zhou et al., 2023; King et al., 2022b)were meta-analyzed, encompassing a cohort of 2,568 patients and seven interventions. The PSRF value for NMA was 1, indicating satisfactory convergence. Heterogeneity analysis and inconsistency testing using the node-splitting method demonstrated that the NMA satisfied both the homogeneity and consistency assumptions (Supplementary Figure S23). Baricitinib (RR [95% CrI] = 1.77 [1.44, 2.20]) exhibited a significant distinction from the control group. The SUCRA values (depicted in Supplementary Figure S24) demonstrated a trend that was in agreement with the forest plot (illustrated in Figure 8) and the league table (Supplementary Table S10): baricitinib (30.42%), brepocitinib (31.84%), deuruxolitinib (41.52%), ivarmacitinib (46.95%), tofacitinib (47.55%), ritlecitinib (56.70%), placebo (62.10%), ruxolitinib (82.93%).

#### CPK

Nine distinct studies (Paracha et al., 2024; King et al., 2024; King et al., 2023; King et al., 2022a; King et al., 2021b; Zhou et al., 2023; King et al., 2022b; ClinicalTrials.gov, 2023) contributed to the analysis, encompassing a total of 3,621 patients and six interventions (as detailed in the reticulation diagram). The PSRF value for NMA was 1, indicating satisfactory convergence. Heterogeneity analysis and inconsistency testing using the node-splitting method showed that the NMA satisfied both the homogeneity and consistency assumptions (Supplementary Figure S25). Compared with the control group, baricitinib (RR [95% CrI] = 1.81 [1.42, 2.36]), deuruxolitinib (RR [95% CrI] = 1.98 [1.11, 3.93]), and ritlecitinib (RR [95% CrI] = 2.31 [1.01, 6.70]) significantly increased the incidence of elevated CPK. In contrast, tofacitinib, ritlecitinib, ivarmacitinib, and brepocitinib did not demonstrate any significant statistical differences in the incidence of serious adverse reactions compared to the control group.

**FIGURE 9:**
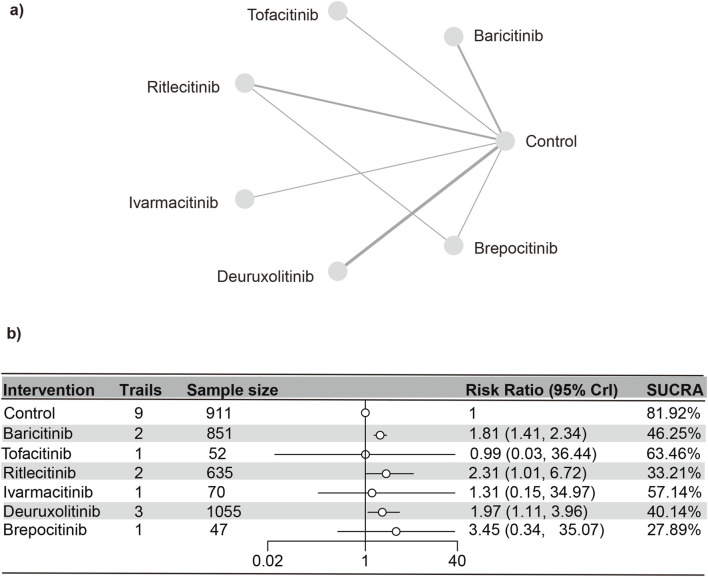
Netplot and forestplot of patients suffering from CPK. **(a)** Netplot of patients with elevated CPK. **(b)** Forestplot of patients with elevated CPK.

The SUCRA values (Supplementary Figure S26) demonstrated a pattern that aligned with the findings presented in the forest plot (Figure 9) and the league table (Supplementary Table S11): brepocitinib (27.89%), ritlecitinib (33.21%), deuruxolitinib (40.14%), baricitinib (46.25%), ivarmacitinib (57.14%), tofacitinib (63.46%), placebo (81.92%).

#### Other adverse reactions

In the incidence of liver function abnormalities and leukopenia up to grades 3–4, no remarkable discrepancies were noted between JAK inhibitors and the control group. Specific results are depicted in Appendices (Supplementary Figures S1, 27–31; Supplementary Tables S12, 13).

### Sensitivity analysis

In the study by King et al. (2023), approximately 10% of the study subjects were aged ≤18 years. The control group in Paracha et al. (2024) was azathioprine. We conducted a sensitivity analysis by excluding this study. More details are provided in Appendices. The results before and after excluding this study showed no significant changes (Supplementary Figures S1–3, S32–37)

Additionally, we conducted a subgroup analysis by splitting the experimental data according to different dose groups. The results revealed that, for acne as an adverse event, Baricitinib 2 mg (RR [95% CrI] = 4.72 [1.92, 14.68]) had a higher incidence than Baricitinib 4 mg (RR [95% CrI] = 4.48 [1.88, 13.78]), while Deuruxolitinib 12 mg (RR [95% CrI] = 3.17 [1.77, 6.15]) showed a higher incidence than Deuruxolitinib 8 mg (RR [95% CrI] = 2.45 [1.39, 4.72]). These results are provided in the supplementary materials. Similarly, for elevated CPK levels, Baricitinib 4 mg (RR [95% CrI] = 16.45 [4.96, 100.60]) showed a higher incidence than Baricitinib 2 mg (RR [95% CrI] = 6.19 [1.64, 40.13]), and Deuruxolitinib 12 mg (RR [95% CrI] = 3.38 [1.55, 8.34]) showed a higher incidence than Deuruxolitinib 4 mg (RR [95% CrI] = 2.57 [1.21, 6.21]). No significant safety differences were observed between different dose groups of the same drug, as detailed in the supplementary materials. For hyperlipidemia, Baricitinib 4 mg (RR [95% CrI] = 2.40 [1.92, 3.06]) had a higher incidence than Baricitinib 2 mg (RR [95% CrI] = 1.92 [1.51, 2.48]). However, no significant safety differences were found between dose groups for upper respiratory tract infections or urinary tract infections. This may be due to: 1) limited literature, which may not capture the differences effectively, and 2) the drugs being well-established, with even higher doses having proven safety in other indications (Supplementary Figures S38–42).

To assess the credibility of the included studies, we employed CINeMA to rate the quality of the evidence, with results presented in a visual format. In direct comparisons, the primary issue of indirectness stemmed from the comparison between Azathioprine and Tofacitinib. Since azathioprine data were combined with placebo data, this approach may have introduced bias, resulting in the downgrading of the evidence quality to “Major concerns.” Another key factor contributing to downgrading was the imprecision of the results. The studies on Ruxolitinib and Tofacitinib exhibited a high risk of within-study bias, which may affect the reliability of the findings. Downgrading factors for indirect comparisons were consistent with those for direct comparisons, primarily including issues of indirectness, imprecision, and heterogeneity (Supplementary Figures S43–47).

## Discussion

This comprehensive NMA suggests that JAK inhibitors are generally safe in the treatment of AA, although there are distinct safety profiles for different drugs. In particular, baricitinib is associated with a more significant risk of detrimental events than other medications, which was not clearly recognized in previous studies.

We observed that the oral administration of certain JAK inhibitors, such as baricitinib and brepocitinib, may increase the risk of specific infections. This finding is consistent with current clinical trial results (King et al., 2021a; King et al., 2022a; King et al., 2021b; King et al., 2022b). Although the mechanism by which JAK inhibitors induce infections has not been fully established, it is suggested that JAK inhibitors elevate the risk of opportunistic infections by impairing the function of leukocytes, particularly T cells and neutrophils. For instance, inflammatory mediators, including TNF-α, IFN-γ, and IL-12, are key factors for inhibiting the maturation of T cells that have a specific immune response to the varicella-zoster virus (VZV) (Mansilla-Polo and Morgado-Carrasco, 2024; Isufi et al., 2025). The production of IFN-γ is regulated by the JAK1-2 signaling pathway, while IL-12 is influenced by the JAK2-TYK2 pathway (van Panhuys et al., 2008). Inhibiting the JAK-STAT signaling pathway disrupts the maturation of VZV-specific immune T cells, potentially increasing the risk of intracellular viral replication and opportunistic infections (Torigo et al., 2000). While our analysis did not demonstrate a statistically significant difference in the inhibition of leukocytes or the likelihood of herpes zoster virus, the reliability of these findings remains uncertain. This is partly due to limited available literature and inconsistent definitions of leukocyte abnormalities across studies. Some studies even did not specify thresholds for leukocyte depletion or the duration of reduced counts. Therefore, our analysis exclusively included studies that clearly defined “neutropenia and lymphocytopenia” as CTCAE grade ≥3 (i.e., absolute neutrophil count <1000/μL or lymphocyte count <200/μL), significantly limiting the studies that could be assessed. Consequently, our findings provide only a preliminary evaluation. Given these results, patients taking oral JAK inhibitors, particularly baricitinib and brepocitinib, should be closely monitored for infectious events. Discontinuation of the drug may be necessary to prevent serious adverse effects. Special care should be taken for patients with pemphigus who have a history of immunosuppression, recurrent urinary tract infections, or cancer.

Skin adverse reactions, including acne (Paracha et al., 2024; King et al., 2024; King et al., 2023; King et al., 2021a; King et al., 2022a; King et al., 2021b; Anderson et al., 2024; Zhou et al., 2023; King et al., 2022b; ClinicalTrials.gov, 2023), folliculitis (Almutairi et al., 2019; King et al., 2021b; Zhou et al., 2023), atopic dermatitis (King et al., 2021b), contact dermatitis (King et al., 2024), urticaria (Zhou et al., 2023), and cellulitis (King et al., 2022a), were widely reported across the 12 articles included in this study. Previous research has demonstrated that JAK is overexpressed in common acne lesions, suggesting that combined JAK1 and JAK2 inhibitors could potentially be therapeutic for acne (Chen et al., 2024; Awad et al., 2021). However, the research indicates that patients administering baricitinib and deuruxolitinib are at a markedly higher risk of acne, implying a more complex association between the administration of JAK inhibitors and acne in AA patients. The results of Chen et al. are consistent with this conclusion. They conducted subgroup analyses of the incidence of acne after the use of JAK inhibitor based on different diseases, showing a significant increase in the incidence of acne in individuals with psoriasis, vitiligo, pemphigus, and atopic dermatitis (Chen et al., 2024). This study further emphasizes that the abnormally elevated likelihood of developing acne after administering JAK inhibitors in these specific populations remains to be elucidated. To mitigate the risk of acne as an adverse effect of JAK inhibitor therapy, a graded management approach is recommended: for mild cases, topical retinoids or antibiotics may be used while maintaining the original treatment regimen with regular follow-up assessments; for moderate-to-severe cases, if lesion progression persists, dose adjustment or temporary interruption of the JAK inhibitor may be considered. All therapeutic adjustments should be based on periodic dermatological evaluations, balancing efficacy, and safety to implement individualized treatment plans.

Most JAK inhibitors prescribed for individuals with AA have been linked to a higher likelihood of developing hyperlipidemia (Papierzewska et al., 2023). Current research suggests that the occurrence of cardiovascular events associated with JAK inhibitors may be closely linked to their multi-target inhibitory effects (Yang et al., 2025). The JAK-STAT signaling pathway, a critical regulator of various factors, when broadly inhibited, may contribute to vascular endothelial dysfunction, an imbalance in the coagulation-fibrinolysis system, and dyslipidemia, among other adverse effects. Particularly, the low selectivity of pan-JAK inhibitors may heighten the risk of cardiovascular events through multiple mechanisms (Al-Rasheed et al., 2016). Hypercholesterolemia is most common in adults taking baricitinib (King et al., 2021b), which is consistent with our study’s findings. In certain cases, patients administering oral ruxolitinib, tofacitinib, and deuruxolitinib exhibited weight increases; however, the incidence of hyperlipidemia was notably lower in patients receiving oral deuruxolitinib and ritlecitinib (Guan et al., 2024). Given these observations, oral deuruxolitinib and ritlecitinib may represent safer therapeutic options for AA patients with hyperlipidemia. To manage the risk of hyperlipidemia induced by JAK inhibitors, a stepwise approach is recommended: prior to treatment, comprehensive lipid profile and cardiovascular risk assessments should be conducted. Lipid levels should be monitored monthly during the initial treatment phase and reassessed every 3 months once stabilized. For mild abnormalities, lifestyle interventions (low-fat diet, regular exercise) are preferred. Statin therapy (e.g., atorvastatin 10–20 mg/d) may be considered for moderate abnormalities. For severe abnormalities (LDL-C ≥190 mg/dL), intensified lipid-lowering therapy is required, along with consideration of dose adjustments for JAK inhibitors. Clinicians should dynamically adjust treatment regimens based on lipid control to ensure medication safety.

We observed that baricitinib and deuruxolitinib significantly increased CPK levels compared to the control group. CPK is predominantly found in cardiac and skeletal muscle, and its elevation is commonly associated with muscle damage or cardiovascular diseases. In 2021, the FDA indicated that patients using JAK inhibitors may face an increased risk of major heart events, including acute myocardial infarction (USFaD, 2021). A subsequent study revealed that compared to those treated with TNF-α inhibitors, patients on baricitinib for rheumatoid arthritis had an elevated risk of encountering adverse cardiovascular events and venous thromboembolism (Register, 2021). In response, the EMA initiated an evaluation of the safety profile of Janus kinase inhibitors in managing inflammatory conditions (Agency). Furthermore, the occurrence of malignancies has been reported in studies involving ivarmacitinib (King et al., 2021b) and baricitinib (King et al., 2022b). Although the incidence remains very low, research indicates that individuals with AA who are administered JAK1 inhibitors may face a heightened likelihood of developing cancer (Papierzewska et al., 2023). The risks of cardiovascular and carcinogenic diseases associated with JAK inhibitors in AA patients are still under investigation, and the current evidence is insufficient. Further research is warranted to clarify these concerns.

This is the first NMA to examine the adverse reactions linked to a range of drugs, so as to clarify the differences in safety between various JAK inhibitors. The study addresses a gap in academic literature, where concerns about the safety of treating AA with JAK inhibitors have persisted, but evidence-based research has been lacking. Notably, this study identifies that baricitinib may pose a higher risk of infections, which was not highlighted in prior research. However, this research has certain limitations. First, the total number of the included studies is fairly low, with moderate quality and limited sample sizes. Second, there are inconsistencies in the assessment criteria for outcome measures, and a number of investigations lacked thorough data concerning some adverse reactions. To confirm and enhance the findings of this study, it is essential to conduct high-quality multicenter clinical trials with a substantial sample size.

## Conclusion

This study demonstrates that oral JAK inhibitors are generally safe for the AA population. However, baricitinib seems to cause an increased likelihood of acne and infections compared to other agents, a finding that has not been clearly identified in prior research. However, this study has several limitations (Dainichi et al., 2024): The number of included studies is limited, with moderate quality and small sample sizes (Craiglow et al., 2024). Variations in the outcome assessment criteria exist, and some studies did not fully report the types of adverse reactions. Therefore, additional high-quality, large-scale, multicenter clinical trials are needed to further confirm the findings of this study (Vañó-Galván et al., 2024). The combination of azathioprine and placebo data in the analysis may have introduced bias. Due to the various limitations of this research, these conclusions should be carefully interpreted. It is important to carry out well-designed, large-scale, multicenter RCTs to support and confirm the findings of this investigation.

## Data Availability

The original contributions presented in the study are included in the article/Supplementary Material, further inquiries can be directed to the corresponding author.
