# Supplementary material for: Safety of oral JAK inhibitors in treating alopecia areata: a systematic review and network meta-analysis
Source: Front Pharmacol. 2025 Aug 11;16:1576553. doi: 10.3389/fphar.2025.1576553 (PMC12375599; doi:10.3389/fphar.2025.1576553)
Supplement: Supplementary file 1 [file Supplementaryfile1.docx]

**Table S1. PRISMA NMA Checklist of Items to Include When Reporting A Systematic Review Involving a Network Meta-analysis**

| **Section/Topic** | **Item #** | **Checklist Item** | **Reported on Page #** |
| --- | --- | --- | --- |
| **TITLE** |  |  |  |
| Title | 1 | Identify the report as a systematic review *incorporating a network meta-analysis (or related form of meta-analysis).* | **Title** |
|  |  |  |  |
| **ABSTRACT** |  |  |  |
| Structured summary | 2 | Provide a structured summary including, as applicable:  **Background:** main objectives  **Methods:** data sources; study eligibility criteria, participants, and interventions; study appraisal; and *synthesis methods, such as network meta-analysis.*  **Results:** number of studies and participants identified; summary estimates with corresponding confidence/credible intervals; *treatment rankings may also be discussed. Authors may choose to summarize pairwise comparisons against a chosen treatment included in their analyses for brevity.*  **Discussion/Conclusions:** limitations; conclusions and implications of findings.  **Other:** primary source of funding; systematic review registration number with registry name. | **Abstract** |
|  |  |  |  |
| **INTRODUCTION** |  |  |  |
| Rationale | 3 | Describe the rationale for the review in the context of what is already known*, including mention of why a network meta-analysis has been conducted.* | Introduction |
| Objectives | 4 | Provide an explicit statement of questions being addressed, with reference to participants, interventions, comparisons, outcomes, and study design (PICOS). | Introduction |
|  |  |  |  |
| **METHODS** |  |  |  |
| Protocol and registration | 5 | Indicate whether a review protocol exists and if and where it can be accessed (e.g., Web address); and, if available, provide registration information, including registration number. | Materials & Methods |
| Eligibility criteria | 6 | Specify study characteristics (e.g., PICOS, length of follow-up) and report characteristics (e.g., years considered, language, publication status) used as criteria for eligibility, giving rationale. *Clearly describe eligible treatments included in the treatment network, and note whether any have been clustered or merged into the same node (with justification).* | Literature Screening |
| Information sources | 7 | Describe all information sources (e.g., databases with dates of coverage, contact with study authors to identify additional studies) in the search and date last searched. | Strategy for Conducting Literature Searches |
| Search | 8 | Present full electronic search strategy for at least one database, including any limits used, such that it could be repeated. | **Supplementary materials**  **Table S1.** |
| Study selection | 9 | State the process for selecting studies (i.e., screening, eligibility, included in systematic review, and, if applicable, included in the meta-analysis). | Literature Screening |
| Data collection process | 10 | Describe method of data extraction from reports (e.g., piloted forms, independently, in duplicate) and any processes for obtaining and confirming data from investigators. | Literature Screening |
| Data items | 11 | List and define all variables for which data were sought (e.g., PICOS, funding sources) and any assumptions and simplifications made. | Literature Screening |
| **Geometry of the network** | **S1** | Describe methods used to explore the geometry of the treatment network under study and potential biases related to it. This should include how the evidence base has been graphically summarized for presentation, and what characteristics were compiled and used to describe the evidence base to readers. |  |
| Risk of bias within individual studies | 12 | Describe methods used for assessing risk of bias of individual studies (including specification of whether this was done at the study or outcome level), and how this information is to be used in any data synthesis. | Evaluation of Potential Bias |
| Summary measures | 13 | State the principal summary measures (e.g., risk ratio, difference in means). *Also describe the use of additional summary measures assessed, such as treatment rankings and surface under the cumulative ranking curve (SUCRA) values, as well as modified approaches used to present summary findings from meta-analyses.* | Statistical Examination |
| Planned methods of analysis | 14 | Describe the methods of handling data and combining results of studies for each network meta-analysis. This should include, but not be limited to:   - *Handling of multi-arm trials;* - *Selection of variance structure;* - *Selection of prior distributions in Bayesian analyses; and* - *Assessment of model fit.* | Statistical Examination |
| **Assessment of Inconsistency** | **S2** | Describe the statistical methods used to evaluate the agreement of direct and indirect evidence in the treatment network(s) studied. Describe efforts taken to address its presence when found. | **Evaluation of Credibility** |
| Risk of bias across studies | 15 | Specify any assessment of risk of bias that may affect the cumulative evidence (e.g., publication bias, selective reporting within studies). | **Evaluation of Publication Bias** |
| Additional analyses | 16 | Describe methods of additional analyses if done, indicating which were pre-specified. This may include, but not be limited to, the following:   - Sensitivity or subgroup analyses; - Meta-regression analyses; - *Alternative formulations of the treatment network; and* - *Use of alternative prior distributions for Bayesian analyses (if applicable).* | Sensitivity Analysis **subgroup analysis** |
|  |  |  |  |
| **RESULTS†** |  |  |  |
| Study selection | 17 | Give numbers of studies screened, assessed for eligibility, and included in the review, with reasons for exclusions at each stage, ideally with a flow diagram. | **The detailed flow chart is shown in Figure 1.** |
| **Presentation of network structure** | **S3** | Provide a network graph of the included studies to enable  visualization of the geometry of the treatment network. | **Figure 4-9, Figure S13-16, Figure S25-26, and Figure S30-37.** |
| **Summary of network geometry** | **S4** | Provide a brief overview of characteristics of the treatment network. This may include commentary on the abundance of trials and randomized patients for the different interventions and pairwise comparisons in the network, gaps of evidence in the treatment network, and potential biases reflected by the network structure. | Literature Acquisition |
| Study characteristics | 18 | For each study, present characteristics for which data were extracted (e.g., study size, PICOS, follow-up period) and provide the citations. | Table 1 |
| Risk of bias within studies | 19 | Present data on risk of bias of each study and, if available, any outcome level assessment. | Figure 2 |
| Results of individual studies | 20 | For all outcomes considered (benefits or harms), present, for each study: 1) simple summary data for each intervention group, and 2) effect estimates and confidence intervals. *Modified approaches may be needed to deal with information from larger networks.* | Statistical Analysis Results |
| Synthesis of results | 21 | Present results of each meta-analysis done, including confidence/credible intervals. *In larger networks, authors may focus on comparisons versus a particular comparator (e.g. placebo or standard care), with full findings presented in an appendix. League tables and forest plots may be considered to summarize pairwise comparisons.* If additional summary measures were explored (such as treatment rankings), these should also be presented. | Risk of Bias Information |
| **Exploration for inconsistency** | **S5** | Describe results from investigations of inconsistency. This may include such information as measures of model fit to compare consistency and inconsistency models, *P* values from statistical tests, or summary of inconsistency estimates from different parts of the treatment network. | **Figures S43-S47** |
| Risk of bias across studies | 22 | Present results of any assessment of risk of bias across studies for the evidence base being studied. | Sensitivity Analysis |
| Results of additional analyses | 23 | Give results of additional analyses, if done (e.g., sensitivity or subgroup analyses, meta-regression analyses*, alternative network geometries studied, alternative choice of prior distributions for Bayesian analyses,* and so forth). | **Subgroup analysis** |
|  |  |  |  |
| **DISCUSSION** |  |  |  |
| Summary of evidence | 24 | Summarize the main findings, including the strength of evidence for each main outcome; consider their relevance to key groups (e.g., healthcare providers, users, and policy-makers). | Discussion |
| Limitations | 25 | Discuss limitations at study and outcome level (e.g., risk of bias), and at review level (e.g., incomplete retrieval of identified research, reporting bias). *Comment on the validity of the assumptions, such as transitivity and consistency. Comment on any concerns regarding network geometry (e.g., avoidance of certain comparisons).*  *For detailed discussion, please refer to the Discussion section.* | Conclusion |
| Conclusions | 26 | Provide a general interpretation of the results in the context of other evidence, and implications for future research. | Conclusion |
|  |  |  |  |
| **FUNDING** |  |  |  |
| Funding | 27 | Describe sources of funding for the systematic review and other support (e.g., supply of data); role of funders for the systematic review. This should also include information regarding whether funding has been received from manufacturers of treatments in the network and/or whether some of the authors are content experts with professional conflicts of interest that could affect use of treatments in the network. | **Funding statement** |

PICOS = population, intervention, comparators, outcomes, study design.

* Text in italics indicateS wording specific to reporting of network meta-analyses that has been added to guidance from the PRISMA statement.

† Authors may wish to plan for use of appendices to present all relevant information in full detail for items in this section.

**Table S2:** Search strategy.

**Embase**

ID Search Hits

#1 MeSH descriptor: [Alopecia Areata] explode all trees 437

#2 (’alopecia areata’ OR ‘allopecia areata maligna’ OR ‘alopecia circumscripta’ OR ‘area celsi’ OR ‘areate alopecia’):ti,ab,kw 792

#3 Janus Kinase Inhibitors 611

#4 (‘baricitinib’ OR ‘brepocitinib’ OR ‘delgocitinib’ OR ‘JAK inhibitor’ OR ‘JAK Inhibitors’ OR ‘Janus kinase inhibitor’ OR ‘Janus kinase inhibitors’ OR ‘Janus tyrosine kinase inhibitor’ OR ‘ritlecitinib’ OR ‘ruxolitinib’ OR ‘tofacitinib’):ti,ab,kw 3733

#5 (#1 OR #2) AND (#3 OR #4) 789

**Cochrane**

No. Query Results

#5 (#1 OR #2) AND (#3 OR #4) 725

#4 'baricitinib':ti,ab,kw OR 'brepocitinib':ti,ab,kw OR 'delgocitinib':ti,ab,kw OR 'jak inhibitor':ti,ab,kw OR 'jak inhibitors':ti,ab,kw OR 'janus kinase inhibitor':ti,ab,kw OR 'janus kinase inhibitors':ti,ab,kw OR 'janus tyrosine kinase inhibitor':ti,ab,kw OR 'ritlecitinib':ti,ab,kw OR 'ruxolitinib':ti,ab,kw OR 'tofacitinib':ti,ab,kw 18229

#3 'janus kinase inhibitors'/exp 33099

#2 'alopecia areata':ti,ab,kw OR 'allopecia areata maligna':ti,ab,kw OR 'alopecia circumscripta':ti,ab,kw OR 'area celsi':ti,ab,kw OR 'areate alopecia':ti,ab,kw 7672

#1 'alopecia areata'/exp 9406

**PubMed**

Search number Query Search Details Results

1. (#1 OR #2) AND (#3 OR #4) ("alopecia areata"[MeSH Terms] OR ("alopecia"[All Fields] AND "areata"[All Fields]) OR "alopecia areata"[All Fields] OR ("alopecia areata"[All Fields] OR ("allopecia"[All Fields] AND "areata"[All Fields] AND ("maligna"[All Fields] OR "malignas"[All Fields])) OR "alopecia circumscripta"[All Fields] OR "area celsi"[All Fields] OR ("areate"[All Fields] AND ("alopecia"[MeSH Terms] OR "alopecia"[All Fields] OR "alopecias"[All Fields])))) AND ("Janus kinase inhibitors"[Pharmacological Action] OR "Janus kinase inhibitors"[MeSH Terms] OR ("janus"[All Fields] AND "kinase"[All Fields] AND "inhibitors"[All Fields]) OR "Janus kinase inhibitors"[All Fields] OR ("baricitinib"[All Fields] OR "brepocitinib"[All Fields] OR "delgocitinib"[All Fields] OR "JAK inhibitor"[All Fields] OR "JAK Inhibitors"[All Fields] OR "Janus kinase inhibitor"[All Fields] OR "Janus kinase inhibitors"[All Fields] OR "Janus tyrosine kinase inhibitor"[All Fields] OR "ritlecitinib"[All Fields] OR "ruxolitinib"[All Fields] OR "tofacitinib"[All Fields])) 1885

4 "baricitinib" OR "brepocitinib" OR "delgocitinib" OR "JAK inhibitor" OR "JAK Inhibitors" OR "Janus kinase inhibitor" OR "Janus kinase inhibitors" OR "Janus tyrosine kinase inhibitor" OR "ritlecitinib" OR "ruxolitinib" OR "tofacitinib" "baricitinib"[All Fields] OR "brepocitinib"[All Fields] OR "delgocitinib"[All Fields] OR "JAK inhibitor"[All Fields] OR "JAK Inhibitors"[All Fields] OR "Janus kinase inhibitor"[All Fields] OR "Janus kinase inhibitors"[All Fields] OR "Janus tyrosine kinase inhibitor"[All Fields] OR "ritlecitinib"[All Fields] OR "ruxolitinib"[All Fields] OR "tofacitinib"[All Fields] 9,610

3 Janus Kinase Inhibitors "janus kinase inhibitors"[Pharmacological Action] OR "janus kinase inhibitors"[MeSH Terms] OR ("janus"[All Fields] AND "kinase"[All Fields] AND "inhibitors"[All Fields]) OR "janus kinase inhibitors"[All Fields] 9,242

2 "alopecia areata" OR "allopecia areata maligna" OR "alopecia circumscripta" OR "area celsi" OR "areate alopecia" "alopecia areata"[All Fields] OR ("allopecia"[All Fields] AND "areata"[All Fields] AND ("maligna"[All Fields] OR "malignas"[All Fields])) OR "alopecia circumscripta"[All Fields] OR "area celsi"[All Fields] OR ("areate"[All Fields] AND ("alopecia"[MeSH Terms] OR "alopecia"[All Fields] OR "alopecias"[All Fields])) 6,003

1 alopecia areata "alopecia areata"[MeSH Terms] OR ("alopecia"[All Fields] AND "areata"[All Fields]) OR "alopecia areata"[All Fields] 6,002

**WOS**

(TI=((“alopecia areata”) OR (“allopecia areata maligna”) OR (“alopecia circumscripta”) OR (“area celsi”) OR (“areate alopecia”)) OR AB=((“alopecia areata”) OR (“allopecia areata maligna”) OR (“alopecia circumscripta”) OR (“area celsi”) OR (“areate alopecia”)) OR AK=((“alopecia areata”) OR (“allopecia areata maligna”) OR (“alopecia circumscripta”) OR (“area celsi”) OR (“areate alopecia”))) AND (TI=((“baricitinib”) OR (“brepocitinib”) OR (“delgocitinib”) OR (“JAK inhibitor”) OR (“JAK Inhibitors”) OR (“Janus kinase inhibitor”) OR (“Janus kinase inhibitors”) OR (“Janus tyrosine kinase inhibitor”) OR (“ritlecitinib”) OR (“ruxolitinib”) OR (“tofacitinib”)) OR AB=((“baricitinib”) OR (“brepocitinib”) OR (“delgocitinib”) OR (“JAK inhibitor”) OR (“JAK Inhibitors”) OR (“Janus kinase inhibitor”) OR (“Janus kinase inhibitors”) OR (“Janus tyrosine kinase inhibitor”) OR (“ritlecitinib”) OR (“ruxolitinib”) OR (“tofacitinib”)) OR AK=((“baricitinib”) OR (“brepocitinib”) OR (“delgocitinib”) OR (“JAK inhibitor”) OR (“JAK Inhibitors”) OR (“Janus kinase inhibitor”) OR (“Janus kinase inhibitors”) OR (“Janus tyrosine kinase inhibitor”) OR (“ritlecitinib”) OR (“ruxolitinib”) OR (“tofacitinib”)))

546 hits

**Table S3.** League Table and SUCRA Values of URTI.

1. League Table of URTI.

|  | Control | Baricitinib | Ruxolitinib | Tofacitinib | Ritlecitinib | Ivarmacitinib | Deuruxolitinib | Brepocitinib |
| --- | --- | --- | --- | --- | --- | --- | --- | --- |
| Control | Control | 1.07(0.78, 1.49) | 0.61(0.01, 31.34) | 2.34(0.19, 68.53) | 0.87(0.6, 1.3) | 1.77 (0.63, 7.72) | 1.04(0.77, 1.46) | **1.72(1.02, 2.91)** |
| Baricitinib | 0.94(0.67, 1.28) | Baricitinib | 0.57(0.01, 29.4) | 2.18(0.18, 63.64) | 0.81(0.49, 1.34) | 1.66 (0.55, 7.43) | 0.98(0.62, 1.54) | 1.6(0.87, 2.97) |
| Ruxolitinib | 1.64(0.03, 90.18) | 1.75(0.03, 96.52) | Ruxolitinib | 3.74(0.44, 101.47) | 1.43(0.03, 79.84) | 3.06 (0.05, 204.22) | 1.71(0.03, 95.95) | 2.8(0.05, 158.86) |
| Tofacitinib | 0.43(0.01, 5.17) | 0.46(0.02, 5.64) | 0.27(0.01, 2.28) | Tofacitinib | 0.37(0.01, 4.63) | 0.78 (0.02, 13.28) | 0.45(0.02, 5.54) | 0.73(0.02, 9.36) |
| Ritlecitinib | 1.16(0.77, 1.68) | 1.23(0.74, 2.03) | 0.7(0.01, 36.64) | 2.69(0.22, 79.25) | Ritlecitinib | 2.05 (0.66, 9.23) | 1.2(0.72, 1.99) | **1.98(1.16, 3.37)** |
| Ivarmacitinib | 0.57(0.13, 1.6) | 0.6(0.13, 1.81) | 0.33(0, 19.53) | 1.28(0.08, 42.1) | 0.49(0.11, 1.51) | Ivarmacitinib | 0.59(0.13, 1.76) | 0.96(0.21, 3.14) |
| Deuruxolitinib | 0.96(0.69, 1.31) | 1.02(0.65, 1.62) | 0.59(0.01, 30.19) | 2.24(0.18, 66.4) | 0.83(0.5, 1.38) | 1.7 (0.57, 7.58) | Deuruxolitinib | 1.64(0.89, 3.04) |
| Brepocitinib | **0.58(0.34, 0.98)** | 0.62(0.34, 1.15) | 0.36(0.01, 18.51) | 1.36 (0.11, 40.97) | **0.51 (0.3, 0.86)** | 1.04 (0.32, 4.81) | 0.61(0.33, 1.12) | Brepocitinib |

1. SUCRA Values of URTI

| Treatment | SUCRA |
| --- | --- |
| Brepocitinib | 0.221966071 |
| Tofacitinib | 0.281396429 |
| Ivarmacitinib | 0.282439286 |
| Baricitinib | 0.550894643 |
| Deuruxolitinib | 0.57905 |
| Control | 0.638308929 |
| Ruxolitinib | 0.673667857 |
| Ritlecitinib | 0.772276786 |

**Table S4.** League Table and SUCRA Values of Urinary Tract Infection.

1. League Table of Urinary Tract Infection.

|  | Control | Baricitinib | Ruxolitinib | Tofacitinib | Ivarmacitinib | Deuruxolitinib | Ritlecitinib |
| --- | --- | --- | --- | --- | --- | --- | --- |
| Control | Control | 2.72(1.14, 8.44) | 1.39(0.03, 60.26) | 1.12(0.03, 38.99) | 2.26(0.34, 53.95) | 0.98(0.3, 4.45) | 1.81(0.48, 12.75) |
| Baricitinib | 0.37(0.12, 0.88) | Baricitinib | 0.49(0.01, 24.39) | 0.4(0.01, 15.72) | 0.83(0.09, 22.32) | 0.36(0.07, 2.05) | 0.66(0.11, 5.67) |
| Ruxolitinib | 0.72(0.02, 33.94) | 2.04(0.04, 108.95) | Ruxolitinib | 0.82(0.2, 2.98) | 1.82(0.02, 216.66) | 0.73(0.01, 43.59) | 1.38(0.03, 97.44) |
| Tofacitinib | 0.9(0.03, 33.05) | 2.51(0.06, 106.22) | 1.23(0.34, 4.91) | Tofacitinib | 2.25(0.04, 224.81) | 0.9(0.02, 42.5) | 1.71(0.04, 97.96) |
| Ivarmacitinib | 0.44(0.02, 2.97) | 1.21(0.04, 11.15) | 0.55(0, 42.05) | 0.44(0, 27.28) | Ivarmacitinib | 0.43(0.01, 4.99) | 0.79(0.03, 11.74) |
| Deuruxolitinib | 1.02(0.22, 3.29) | 2.81(0.49, 14.39) | 1.37(0.02, 72.48) | 1.11(0.02, 46.33) | 2.31(0.2, 68.14) | Deuruxolitinib | 1.86(0.25, 17.79) |
| Ritlecitinib | 0.55(0.08, 2.09) | 1.51(0.18, 8.76) | 0.72(0.01, 38.83) | 0.59(0.01, 25.54) | 1.26(0.09, 36.43) | 0.54(0.06, 4.04) | Ritlecitinib |

1. SUCRA Values of Urinary Tract Infection.

| Treatment | SUCRA |
| --- | --- |
| Baricitinib | 0.257495833 |
| Ivarmacitinib | 0.368925 |
| Ritlecitinib | 0.42210625 |
| Ruxolitinib | 0.510175 |
| Tofacitinib | 0.591566667 |
| Deuruxolitinib | 0.660329167 |
| Control | 0.689402083 |

**Table S5.** League Table and SUCRA Values of ACNE.

1. League Table of ACNE.

|  | Control | Baricitinib | Ritlecitinib | Ivarmacitinib | Deuruxolitinib | Brepocitinib | Tofacitinib |
| --- | --- | --- | --- | --- | --- | --- | --- |
| Control | Control | 4.66(2.0, 13.44) | 1.84(0.95, 3.95) | 1.84(0.25, 55.52) | 2.74(1.58, 5.29) | 2.06(0.58, 6.97) | 1.13(0.03, 42.65) |
| Baricitinib | 0.21(0.07, 0.5) | Baricitinib | 0.39(0.12, 1.24) | 0.4(0.04, 13.32) | 0.59(0.18, 1.71) | 0.43(0.09, 1.98) | 0.24(0.01, 9.52) |
| Ritlecitinib | 0.54(0.25, 1.05) | 2.55(0.8, 8.69) | Ritlecitinib | 1.01(0.12, 30.02) | 1.49(0.59, 3.76) | 1.11(0.33, 3.48) | 0.61(0.02, 24.24) |
| Ivarmacitinib | 0.54(0.02, 4.08) | 2.52(0.08, 25.5) | 0.99(0.03, 8.63) | Ivarmacitinib | 1.49(0.05, 12.36) | 1.07(0.03, 11.75) | 0.56(0, 36.71) |
| Deuruxolitinib | 0.37(0.19, 0.63) | 1.69(0.59, 5.66) | 0.67(0.27, 1.7) | 0.67(0.08, 21.36) | Deuruxolitinib | 0.74(0.18, 2.86) | 0.41(0.01, 15.63) |
| Brepocitinib | 0.49(0.14, 1.73) | 2.31(0.5, 11.76) | 0.9(0.29, 3.02) | 0.94(0.09, 31.7) | 1.35(0.35, 5.5) | Brepocitinib | 0.56(0.01, 24.83) |
| Tofacitinib | 0.88(0.02, 32.48) | 4.2(0.11, 168.36) | 1.63(0.04, 63.59) | 1.79(0.03, 210.65) | 2.45(0.06, 93.33) | 1.8(0.04, 78.34) | Tofacitinib |

1. SUCRA Values of ACNE.

| Treatment | SUCRA |
| --- | --- |
| Baricitinib | 0.13670625 |
| Deuruxolitinib | 0.338425 |
| Brepocitinib | 0.489277083 |
| Ivarmacitinib | 0.5108625 |
| Ritlecitinib | 0.539522917 |
| Tofacitinib | 0.639891667 |
| Control | 0.845314583 |

**Table S6.** League Table and SUCRA Values of Herpes Zoster.

1. League Table of Herpes Zoster.

|  | Control | Baricitinib | Ruxolitinib | Tofacitinib | Deuruxolitinib |
| --- | --- | --- | --- | --- | --- |
| Control | Control | 1.75(0.56, 7.54) | 2.54(0.04,244.53) | 1.05(0.03,28.87) | 0.84(0.1,18.16) |
| Baricitinib | 0.57(0.13,1.78) | Baricitinib | 1.4(0.02,153.08) | 0.57(0.01,19.35) | 0.48(0.04,12.3) |
| Ruxolitinib | 0.39(0, 28.27) | 0.72(0.01, 61.35) | Ruxolitinib | 0.43(0.02, 4.89) | 0.36(0,58.17) |
| Tofacitinib | 0.95(0.03,31.48) | 1.74(0.05, 71.76) | 2.32(0.2, 59.99) | Tofacitinib | 0.88(0.02,75.51) |
| Deuruxolitinib | 1.18(0.06,10.13) | 2.09(0.08, 28.53) | 2.77(0.02,442.55) | 1.14(0.01,62.21) | Deuruxolitinib |

1. SUCRA Values of Herpes Zoster.

| Treatment | SUCRA |
| --- | --- |
| Ruxolitinib | 0.338659375 |
| Baricitinib | 0.353146875 |
| Tofacitinib | 0.58528125 |
| Deuruxolitinib | 0.609159375 |
| Control | 0.613753125 |

**Table S7.** League Table and SUCRA Values of Neurological Symptoms.

1. League Table of Neurological Symptoms.

|  | Control | Baricitinib | Ruxolitinib | Tofacitinib | Ritlecitinib | Ivarmacitinib | Deuruxolitinib | Brepocitinib |
| --- | --- | --- | --- | --- | --- | --- | --- | --- |
| Control | Control | 1.17(0.72,2.01) | 2.35(0.08,118.36) | 2.3(0.2,60.19) | 1.38(0.92,2.14) | 0.35(0.04,3.11) | 1.36(0.96,1.98) | 0.66(0.18,1.83) |
| Baricitinib | 0.85(0.5,1.39) | Baricitinib | 1.99(0.07,106.34) | 1.95(0.15,53.9) | 1.17(0.6,2.28) | 0.29(0.03,2.81) | 1.16(0.61,2.16) | 0.55(0.14,1.76) |
| Ruxolitinib | 0.43(0.01,11.91) | 0.5(0.01,14.66) | Ruxolitinib | 1.01(0.11,8.92) | 0.59(0.01,17.04) | 0.15(0,7.98) | 0.58(0.01,16.59) | 0.27(0,9.06) |
| Tofacitinib | 0.43(0.02,5.12) | 0.51(0.02,6.5) | 0.99(0.11,8.83) | Tofacitinib | 0.6(0.02,7.5) | 0.15(0,4.21) | 0.59(0.02,7.31) | 0.27(0.01,4.19) |
| Ritlecitinib | 0.73(0.47,1.08) | 0.85(0.44,1.66) | 1.7(0.06,86.2) | 1.67(0.13,44.29) | Ritlecitinib | 0.25(0.03,2.33) | 0.99(0.57,1.73) | 0.48(0.13,1.29) |
| Ivarmacitinib | 2.88(0.32,26.22) | 3.41(0.36,33.18) | 6.87(0.13,576.96) | 6.88(0.24,329.83) | 3.99(0.43,37.48) | Ivarmacitinib | 3.95(0.43,37.27) | 1.85(0.15,21.33) |
| Deuruxolitinib | 0.73(0.5,1.04) | 0.86(0.46,1.63) | 1.72 (0.06,87.63) | 1.69(0.14,45.32) | 1.01(0.58,1.76) | 0.25(0.03,2.34) | Deuruxolitinib | 0.48(0.13,1.42) |
| Brepocitinib | 1.53(0.55,5.43) | 1.81(0.57,7.09) | 3.72(0.11,218.04) | 3.65(0.24,114.72) | 2.1(0.78,7.43) | 0.54(0.05,6.57) | 2.09(0.7,7.85) | Brepocitinib |

1. SUCRA Values of Neurological Symptoms.

| Treatment | SUCRA |
| --- | --- |
| Tofacitinib | 0.293130357142857 |
| Ritlecitinib | 0.330528571428571 |
| Ruxolitinib | 0.332257142857143 |
| Deuruxolitinib | 0.336567857142857 |
| Baricitinib | 0.467003571428571 |
| Control | 0.635283928571429 |
| Brepocitinib | 0.761680357142857 |
| Ivarmacitinib | 0.843548214285714 |

**Table S8.** League Table and SUCRA Values of Digestive System Disorders.

a) League Table of Digestive System Disorders.

|  | Control | Baricitinib | Ruxolitinib | Tofacitinib | Ritlecitinib | Ivarmacitinib | Deuruxolitinib | Brepocitinib |
| --- | --- | --- | --- | --- | --- | --- | --- | --- |
| Control | Control | 3.38(0.51,73.09) | 1.6(0.03,94.03) | 0.43(0.01,5.26) | 0.88 (0.58, 1.37) | 1.28 (0.15,32.08) | 1.05 (0.58, 2.04) | 0.78 (0.33, 1.67) |
| Baricitinib | 0.3(0.01,1.97) | Baricitinib | 0.42(0,41.44) | 0.11(0,2.98) | 0.26 (0.01, 1.84) | 0.37(0.01, 14.38) | 0.31 (0.01, 2.35) | 0.22 (0.01, 1.82) |
| Ruxolitinib | 0.63(0.01,34.09) | 2.36(0.02,311.15) | Ruxolitinib | 0.27(0.01,2.37) | 0.55 (0.01, 30.21) | 0.88 (0.01, 129.42) | 0.66 (0.01, 37.89) | 0.48 (0.01, 28.02) |
| Tofacitinib | 2.35(0.19,71.11) | 9.02(0.34,735.75) | 3.67(0.42,102.29) | Tofacitinib | 2.07 (0.16, 64.51) | 3.38 (0.11, 312.16) | 2.5 (0.19, 79.81) | 1.82 (0.13, 59.87) |
| Ritlecitinib | 1.14(0.73, 1.74) | 3.86(0.54,87.39) | 1.81(0.03,107.31) | 0.48(0.02,6.09) | Ritlecitinib | 1.46 (0.16, 37.61) | 1.2 (0.57, 2.64) | 0.88 (0.37, 1.89) |
| Ivarmacitinib | 0.78(0.03, 6.68) | 2.7(0.07,109) | 1.14(0.01,125.13) | 0.3(0,9.26) | 0.69 (0.03, 6.22) | Ivarmacitinib | 0.82 (0.03, 7.79) | 0.6 (0.02, 5.97) |
| Deuruxolitinib | 0.95(0.49, 1.73) | 3.21(0.42,74.56) | 1.51(0.03,91.92) | 0.4(0.01,5.33) | 0.83 (0.38, 1.77) | 1.22 (0.13, 32.12) | Deuruxolitinib | 0.73 (0.25, 1.96) |
| Brepocitinib | 1.28(0.6, 3.07) | 4.48(0.55,111.11) | 2.08(0.04,131.07) | 0.55(0.02,7.81) | 1.13 (0.53, 2.72) | 1.68 (0.17, 46.05) | 1.36 (0.51, 4) | Brepocitinib |

b) SUCRA Values of Digestive System Disorders.

| Treatment | SUCRA |
| --- | --- |
| Baricitinib | 0.169166071 |
| Ruxolitinib | 0.399871429 |
| Ivarmacitinib | 0.438507143 |
| Deuruxolitinib | 0.468353571 |
| Control | 0.488730357 |
| Ritlecitinib | 0.601667857 |
| Brepocitinib | 0.661391071 |
| Tofacitinib | 0.7723125 |

**Table S9.** League Table and SUCRA Values of Dermatitis.

a）League Table of Dermatitis.

|  | Control | Ritlecitinib | Ivarmacitinib | Deuruxolitinib | Brepocitinib |
| --- | --- | --- | --- | --- | --- |
| Control | Control | 5.04(0.68,133.65) | 1.3(0.15, 31.82) | 0.36(0.09, 1.5) | 2.35(0.2, 68.65) |
| Ritlecitinib | 0.2(0.01,1.47) | Ritlecitinib | 0.25(0.01, 11.07) | 0.07(0, 0.84) | 0.47(0.06, 2.43) |
| Ivarmacitinib | 0.77(0.03,6.54) | 3.97(0.09,188.11) | Ivarmacitinib | 0.27(0.01, 3.68) | 1.79(0.03, 92.93) |
| Deuruxolitinib | 2.81(0.67,10.97) | 14.55(1.19,508.83) | 3.74(0.27, 107.53) | Deuruxolitinib | 6.62(0.36, 248.74) |
| Brepocitinib | 0.43 (0.01,5.11) | 2.15(0.41,16.91) | 0.56(0.01, 31.28) | 0.15(0, 2.75) | Brepocitinib |

b）SUCRA Values of Dermatitis.

| Treatment | SUCRA |
| --- | --- |
| Ritlecitinib | 0.121896875 |
| Brepocitinib | 0.38623125 |
| Ivarmacitinib | 0.4949875 |
| Control | 0.58649375 |
| Deuruxolitinib | 0.910390625 |

**Table S10.** League Table and SUCRA Values of Hyperlipidemia.

a) League Table of Hyperlipidemia.

|  | Control | Baricitinib | Ruxolitinib | Tofacitinib | Ritlecitinib | Ivarmacitinib | Deuruxolitinib | Brepocitinib |
| --- | --- | --- | --- | --- | --- | --- | --- | --- |
| Control | Control | 1.7 (1.44,2.2) | 0.2 (0, 12.66) | 1.14(0.03,40.02) | 0.98 (0.11, 8.63) | 1.26 (0.15, 32.55) | 1.45 (0.59, 4.37) | 2.17 (0.21, 27.79) |
| Baricitinib | 0.56(0.45,0.69) | Baricitinib | 0.11(0, 7.22) | 0.65(0.02,22.88) | 0.55 (0.06, 4.93) | 0.71 (0.08, 18.6) | 0.82 (0.33, 2.53) | 1.23 (0.11, 15.78) |
| Ruxolitinib | 4.98(0.08, 528) | 8.81(0.14,931.93) | Ruxolitinib | 5.2 (0.7, 135.52) | 4.92(0.05,778.85) | 7.02(0.06,1680.45) | 7.41 (0.1,909.92) | 11.3 (0.1, 2020.06) |
| Tofacitinib | 0.88(0.02,31.2) | 1.55(0.04, 56.12) | 0.19(0.01,1.43) | Tofacitinib | 0.86(0.01, 53.81) | 1.2 (0.02, 125.88) | 1.3 (0.03, 54.79) | 1.93 (0.03, 147.34) |
| Ritlecitinib | 1.02(0.12,9.13) | 1.81(0.2, 16.55) | 0.2(0, 22.13) | 1.16(0.02, 75.2) | Ritlecitinib | 1.35 (0.06, 61.31) | 1.52 (0.14, 17.1) | 2.23 (0.2, 29.27) |
| Ivarmacitinib | 0.79(0.03,6.76) | 1.41(0.05, 12.13) | 0.14(0, 16.65) | 0.83(0.01,58.29) | 0.74 (0.02, 16.27) | Ivarmacitinib | 1.16 (0.04,13.16) | 1.64 (0.03, 46.7) |
| Deuruxolitinib | 0.69(0.23,1.69) | 1.22(0.39, 3.06) | 0.13(0, 9.7) | 0.77(0.02,31.27) | 0.66 (0.06, 7.09) | 0.86 (0.08, 25) | Deuruxolitinib | 1.47 (0.11, 21.76) |
| Brepocitinib | 0.46(0.04,4.86) | 0.81(0.06, 8.74) | 0.09(0, 10.42) | 0.52(0.01,35.81) | 0.45 (0.03, 4.89) | 0.61 (0.02, 29.82) | 0.68 (0.05, 8.97) | Brepocitinib |

b) SUCRA Values of Hyperlipidemia.

| Treatment | SUCRA |
| --- | --- |
| Baricitinib | 0.304226786 |
| Brepocitinib | 0.318383929 |
| Deuruxolitinib | 0.41515 |
| Ivarmacitinib | 0.469494643 |
| Tofacitinib | 0.475525 |
| Ritlecitinib | 0.56695 |
| Control | 0.620925 |
| Ruxolitinib | 0.829344643 |

**Table S11.** League Table and SUCRA Values of CPK.

1. League Table of CPK.

|  | Control | Baricitinib | Tofacitinib | Ritlecitinib | Ivarmacitinib | Deuruxolitinib | Brepocitinib |
| --- | --- | --- | --- | --- | --- | --- | --- |
| Control | Control | 1.8(1.41, 2.34) | 0.99(0.03, 36.44) | 2.31(1.01, 6.7) | 1.31(0.15, 34.97) | 1.97(1.11, 3.96) | 3.45(0.34, 35.07) |
| Baricitinib | 0.55(0.43, 0.71) | Baricitinib | 0.55(0.01, 20.12) | 1.28(0.54, 3.83) | 0.72(0.08, 19.16) | 1.09(0.58, 2.29) | 1.9(0.19, 19.84) |
| Tofacitinib | 1.01(0.03, 38.51) | 1.82(0.05, 69.91) | Tofacitinib | 2.4(0.06, 102.12) | 1.42(0.02, 167.46) | 2.02(0.05, 80.45) | 3.48(0.05, 258.47) |
| Ritlecitinib | 0.43(0.15, 0.99) | 0.78(0.26, 1.87) | 0.42(0.01, 16.91) | Ritlecitinib | 0.56(0.05, 16.5) | 0.85(0.26, 2.52) | 1.45(0.15, 13.32) |
| Ivarmacitinib | 0.77(0.03, 6.86) | 1.38(0.05, 12.62) | 0.7(0.01, 49.97) | 1.79(0.06, 20.56) | Ivarmacitinib | 1.52(0.06, 15.06) | 2.49(0.05, 64.77) |
| Deuruxolitinib | 0.51(0.25, 0.9) | 0.91(0.44, 1.73) | 0.5(0.01, 19) | 1.17(0.4, 3.9) | 0.66(0.07, 17.52) | Deuruxolitinib | 1.72(0.16, 19.36) |
| Brepocitinib | 0.29(0.03, 2.91) | 0.53(0.05, 5.33) | 0.29(0, 19.61) | 0.69(0.08, 6.45) | 0.4(0.02, 19.71) | 0.58(0.05, 6.34) | Brepocitinib |

1. SUCRA Values of CPK.

| Treatment | SUCRA |
| --- | --- |
| Brepocitinib | 0.278927083 |
| Ritlecitinib | 0.33205625 |
| Deuruxolitinib | 0.401377083 |
| Baricitinib | 0.46245 |
| Ivarmacitinib | 0.571397917 |
| Tofacitinib | 0.634577083 |
| Control | 0.819214583 |

**Table S12.** League Table and SUCRA Values of Abnormal Liver Function.

- 1. League Table of Abnormal Liver Function.

|  | Control | Baricitinib | Ruxolitinib | Tofacitinib | Ritlecitinib | Ivarmacitinib | Deuruxolitinib | Brepocitinib |
| --- | --- | --- | --- | --- | --- | --- | --- | --- |
| Control | Control | 0.62(0.33,1.16) | 0.39(0.01,11.17) | 0.42(0.01,5.16) | 0.97(0.02,37.94) | 0.82(0.07,24) | 0.55 (0.09, 4.57) | 3.8(0.42,116.19) |
| Baricitinib | 1.62(0.86,3) | Baricitinib | 0.62(0.01,19.12) | 0.67(0.02,9.05) | 1.56(0.04,65.25) | 1.34(0.1,40.86) | 0.89 (0.12, 8.13) | 6.2(0.62,196.05) |
| Ruxolitinib | 2.59(0.09,139.63) | 1.61(0.05,91.27) | Ruxolitinib | 1.04(0.11,9.25) | 2.56(0.02,537.16) | 2.36(0.03,356.29) | 1.49 (0.03, 123.9) | 11(0.17,1792.63) |
| Tofacitinib | 2.4(0.19,74.24) | 1.5(0.11,48.2) | 0.96(0.11,8.8) | Tofacitinib | 2.46(0.03,329.7) | 2.21(0.06,231.46) | 1.4 (0.05, 71.95) | 10.31(0.31,1115) |
| Ritlecitinib | 1.04(0.03,41.13) | 0.64(0.02,26.99) | 0.39(0,56.38) | 0.41(0,35.55) | Ritlecitinib | 0.91(0.01,114.16) | 0.59 (0.01, 38.36) | 3.91(0.44,121.16) |
| Ivarmacitinib | 1.21(0.04,14.37) | 0.75(0.02,9.58) | 0.42(0,31.96) | 0.45(0,17.79) | 1.1(0.01,92.97) | Ivarmacitinib | 0.66 (0.01, 17.45) | 4.68(0.09,292.55) |
| Deuruxolitinib | 1.81(0.22,11.66) | 1.12(0.12,8.05) | 0.67(0.01,34.74) | 0.71(0.01,18.37) | 1.7(0.03,106.12) | 1.52(0.06,69.06) | Deuruxolitinib | 7.04(0.32,329.75) |
| Brepocitinib | 0.26(0.01,2.38) | 0.16(0.01,1.62) | 0.09(0,5.96) | 0.1(0,3.24) | 0.26(0.01,2.3) | 0.21(0,11.35) | 0.14 (0, 3.11) | Brepocitinib |

b) SUCRA Values of Abnormal Liver Function

| Treatment | SUCRA |
| --- | --- |
| Brepocitinib | 0.121898214 |
| Control | 0.384244643 |
| Ritlecitinib | 0.478544643 |
| Ivarmacitinib | 0.481946429 |
| Deuruxolitinib | 0.602101786 |
| Baricitinib | 0.613875 |
| Ruxolitinib | 0.653191071 |
| Tofacitinib | 0.664198214 |

**Table S13.** League Table and SUCRA Values of Leukopenia Grade 3-4.

1. League Table of leukopenia grade3-4.

|  | Control | Baricitinib | Tofacitinib | Ritlecitinib | Ivarmacitinib | Deuruxolitinib | Brepocitinib | Ruxolitinib |
| --- | --- | --- | --- | --- | --- | --- | --- | --- |
| Control | Control | 1.81(0.57,8.27) | 1.1(0.03,37.77) | 1.56(0.25,12.23) | 0.35(0.01,11.69) | 2.28(0.76,11.16) | 2.81(0.39,27) | 1.01(0.01,145.84) |
| Baricitinib | 0.55(0.12,1.77) | Baricitinib | 0.59(0.01,24.28) | 0.84(0.08,9.36) | 0.18(0, 7.92) | 1.26(0.2,8.7) | 1.51(0.13, 19.97) | 0.54(0,89.35) |
| Tofacitinib | 0.91(0.03,35.69) | 1.7(0.04,85.43) | Tofacitinib | 1.46(0.03,90.19) | 0.32(0,47.76) | 2.19(0.05,109.28) | 2.61(0.05,178.21) | 0.93(0.02, 31.49) |
| Ritlecitinib | 0.64(0.08,4.08) | 1.2(0.11,12.57) | 0.69(0.01,36.82) | Ritlecitinib | 0.22(0,11.84) | 1.51(0.14,15.97) | 1.77(0.28,12.91) | 0.63(0,132.55) |
| Ivarmacitinib | 2.88(0.09,101.67) | 5.44(0.13,244.12) | 3.11(0.02,479.04) | 4.59(0.08,262.89) | Ivarmacitinib | 6.96(0.16,309.53) | 8.24(0.14,520.65) | 2.85(0.01,1295.84) |
| Deuruxolitinib | 0.44(0.09,1.31) | 0.79(0.11,5.09) | 0.46(0.01,19.15) | 0.66(0.06,7.13) | 0.14(0, 6.08) | Deuruxolitinib | 1.19(0.1,15.11) | 0.42(0,71) |
| Brepocitinib | 0.36(0.04,2.56) | 0.66(0.05,7.83) | 0.38(0.01,21.06) | 0.56(0.08,3.55) | 0.12(0, 6.94) | 0.84(0.07,9.78) | Brepocitinib | 0.35(0,74.81) |
| Ruxolitinib | 0.99(0.01,165.71) | 1.86(0.01,385.4) | 1.07(0.03,41.88) | 1.59(0.01,367.72) | 0.35(0,161.92) | 2.37(0.01,474.71) | 2.87(0.01,708.4) | Ruxolitinib |

1. SUCRA Values of leukopenia grade3-4.

| Treatment | SUCRA |
| --- | --- |
| Brepocitinib | 0.288096428571429 |
| Deuruxolitinib | 0.317389285714286 |
| Baricitinib | 0.398426785714286 |
| Ritlecitinib | 0.472598214285714 |
| Tofacitinib | 0.547792857142857 |
| Ruxolitinib | 0.548860714285714 |
| Control | 0.652466071428571 |
| Ivarmacitinib | 0.774369642857143 |


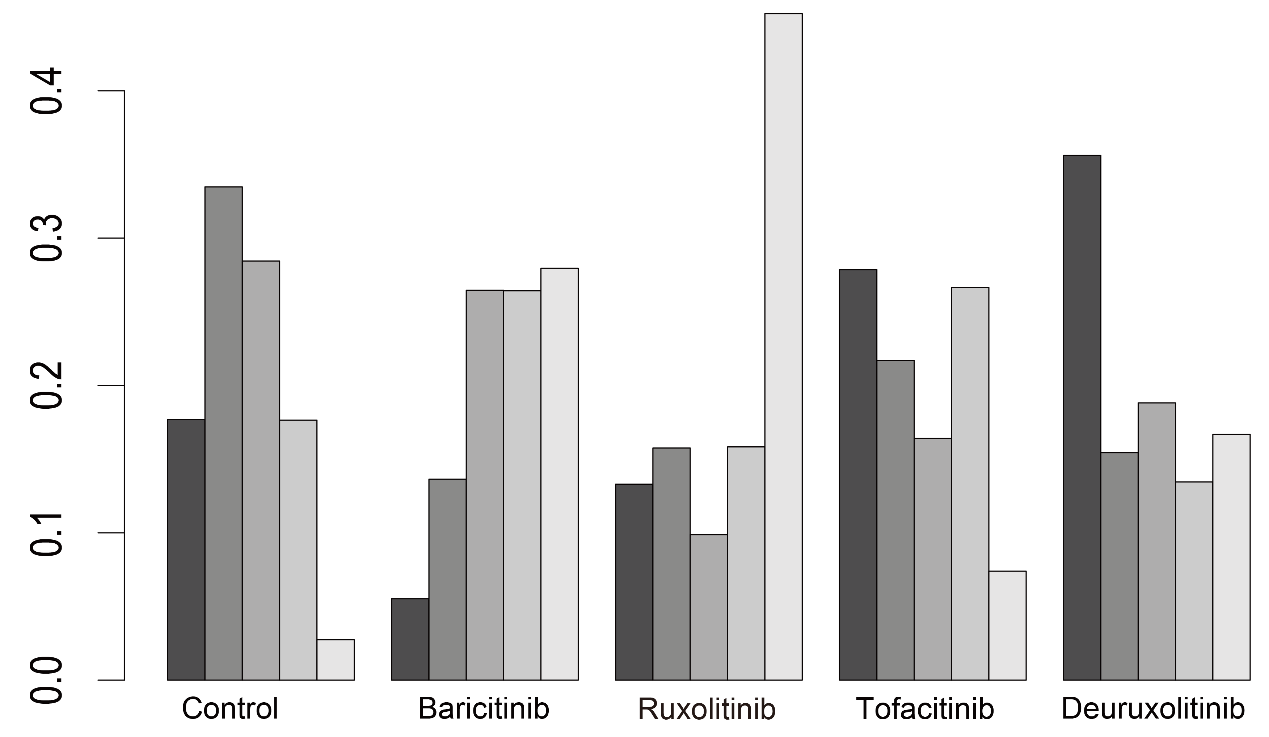


**Figure S1**. Probability Ranking Chart of Herpes Zoster.


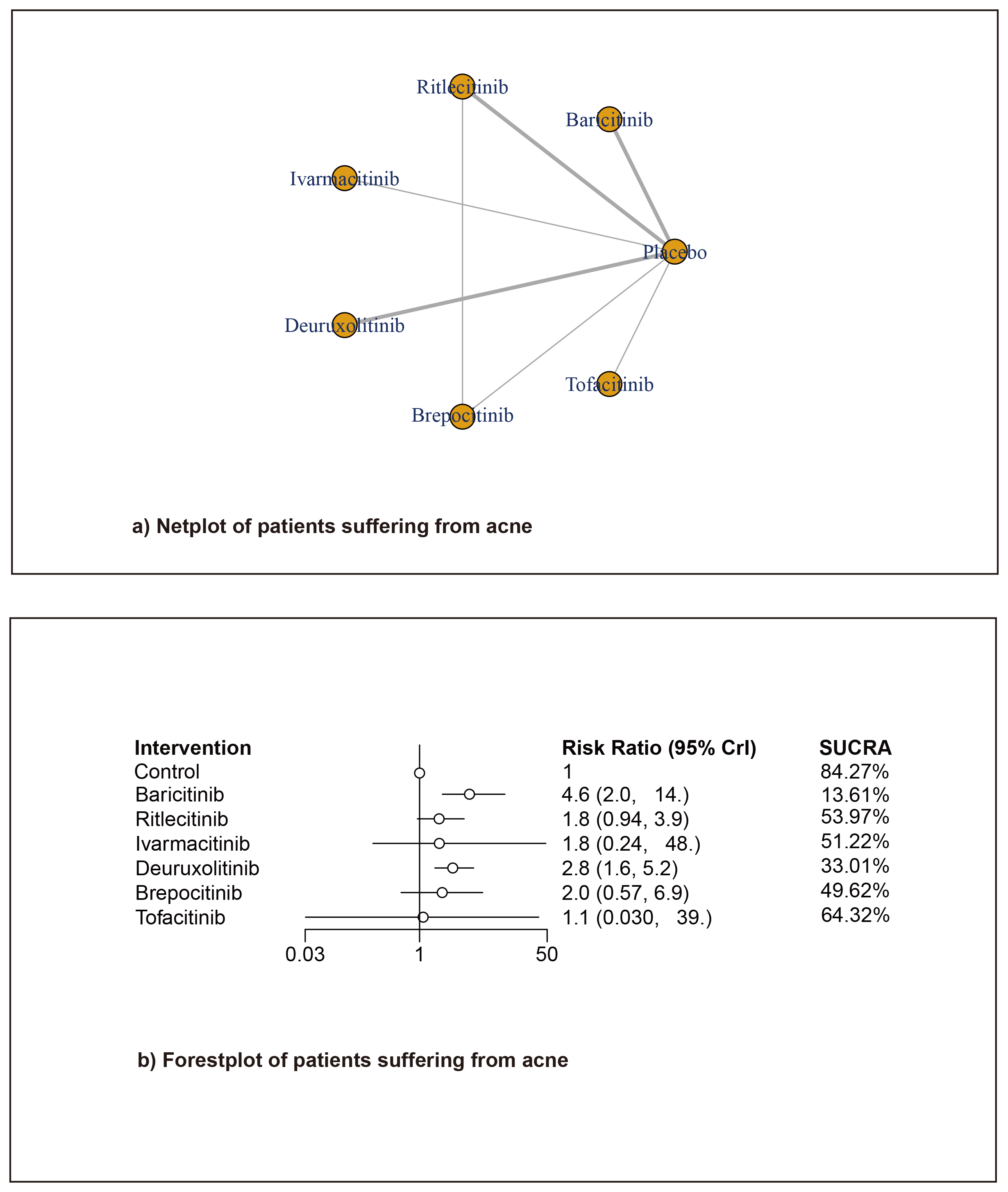


**Figure S2.** Netplot and forestplot of patients suffering from Acne.


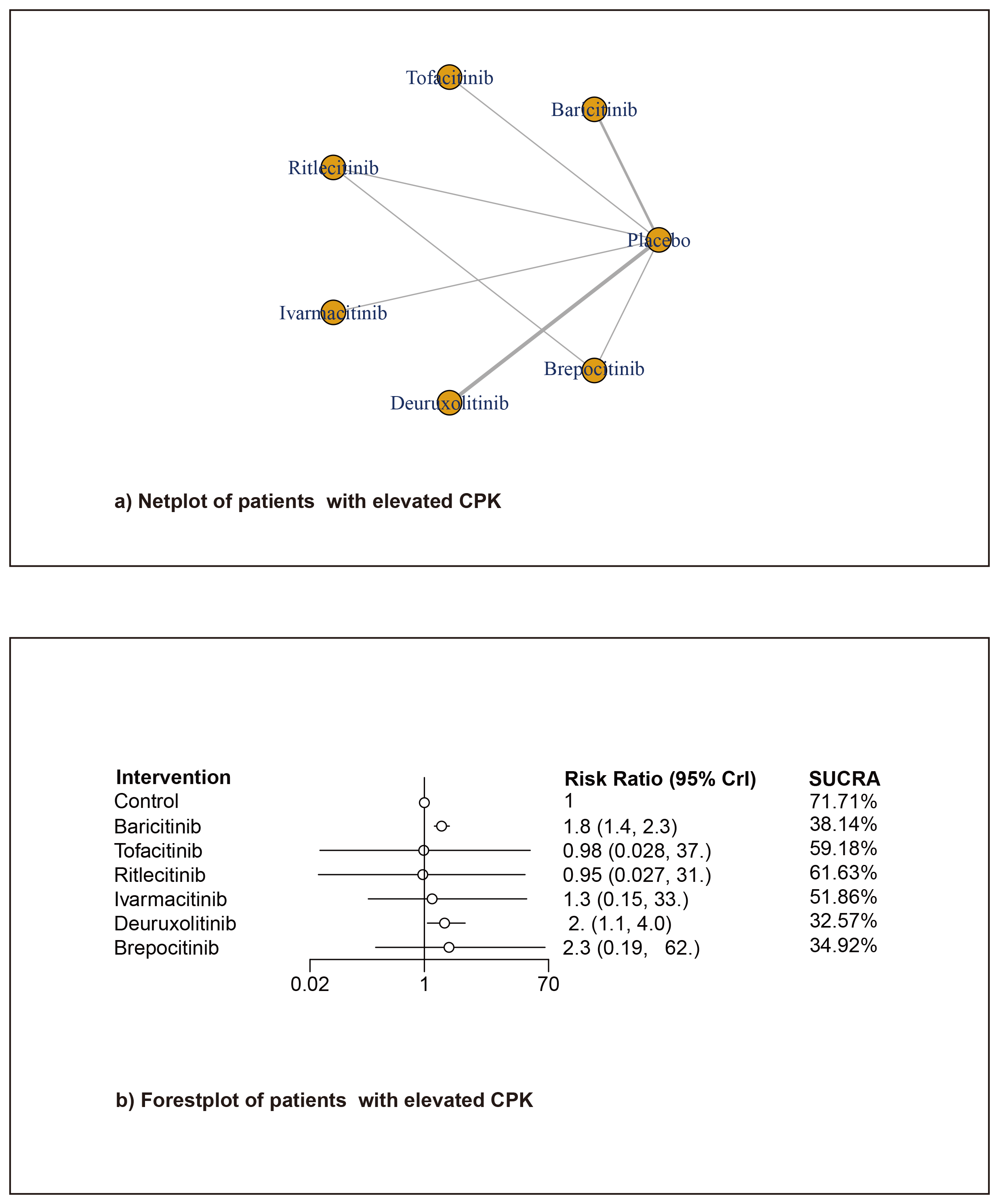


**Figure S3.** Netplot and forestplot of patients suffering from CPK.

**
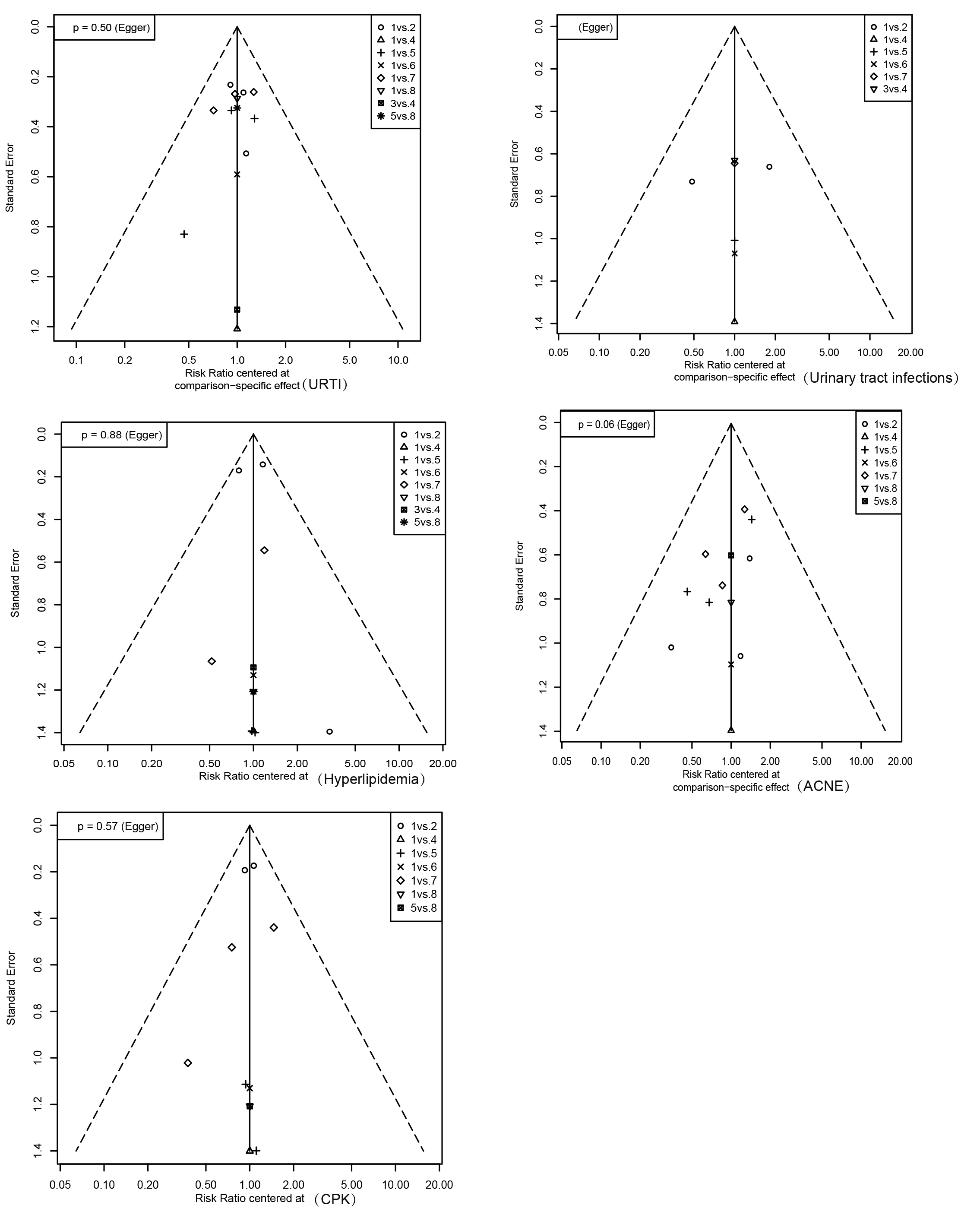
**

**Figure S4** Funnel Plot of Experimental Results

**
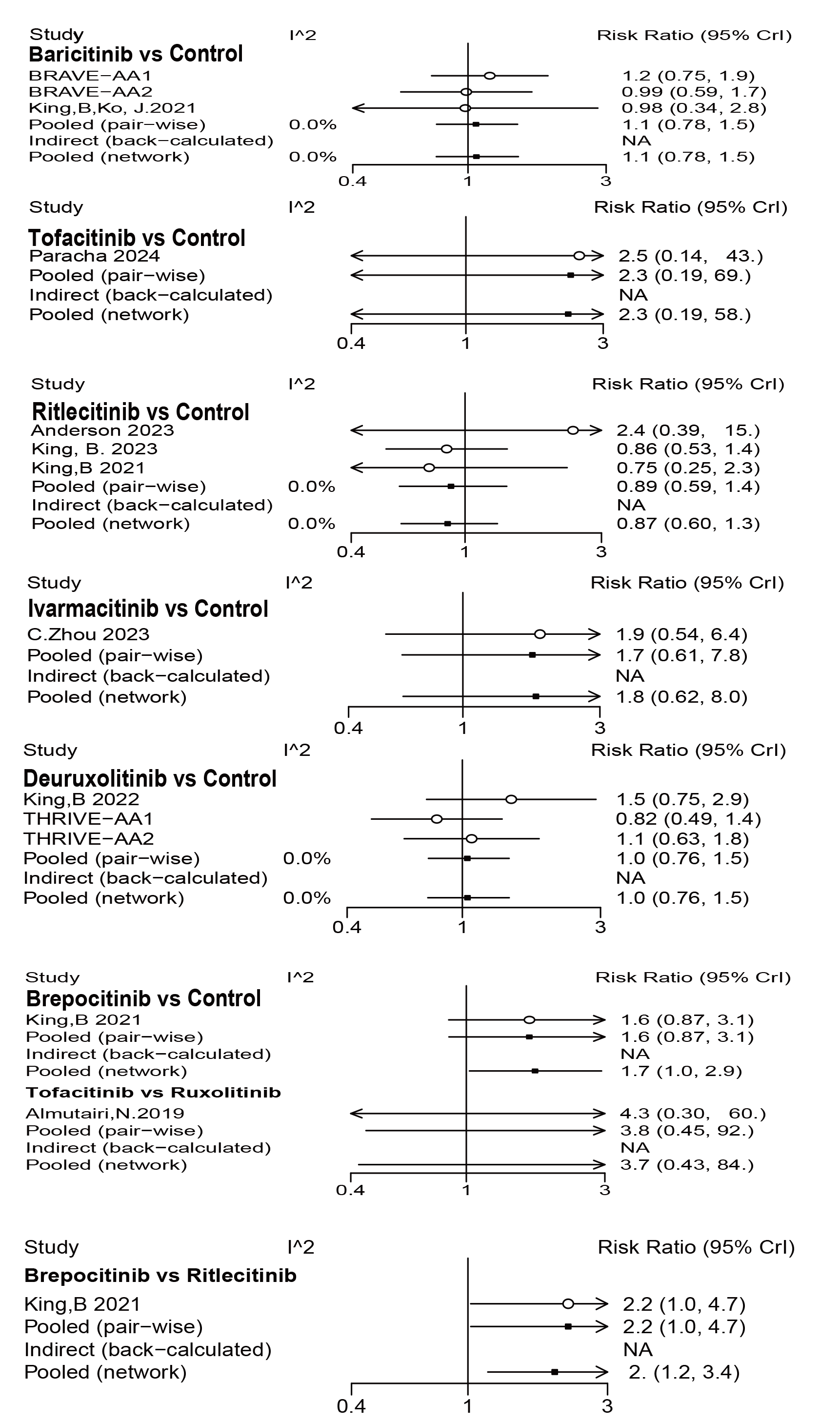
**

**Figure S5**. Heterogeneity test of URTI.


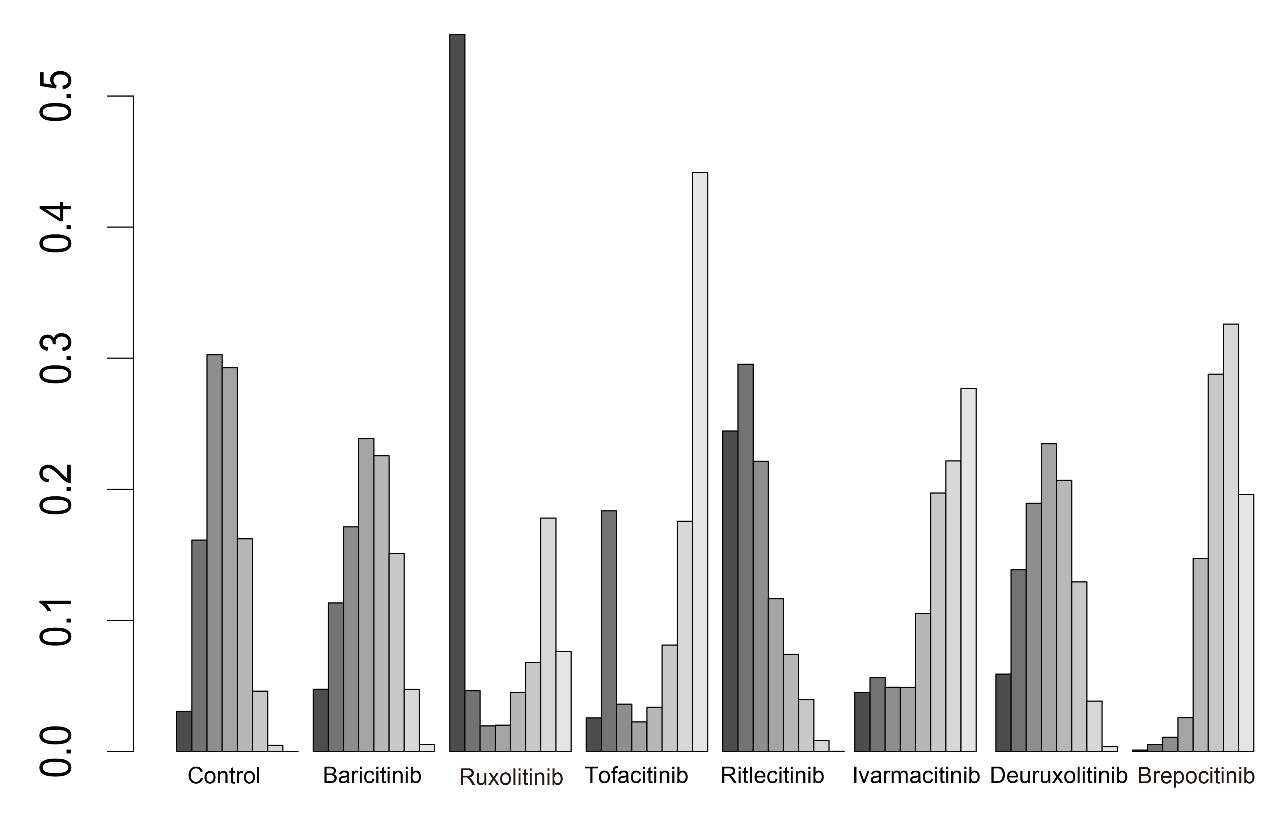


**Figure S6.** Probability Ranking Chart of URTI.


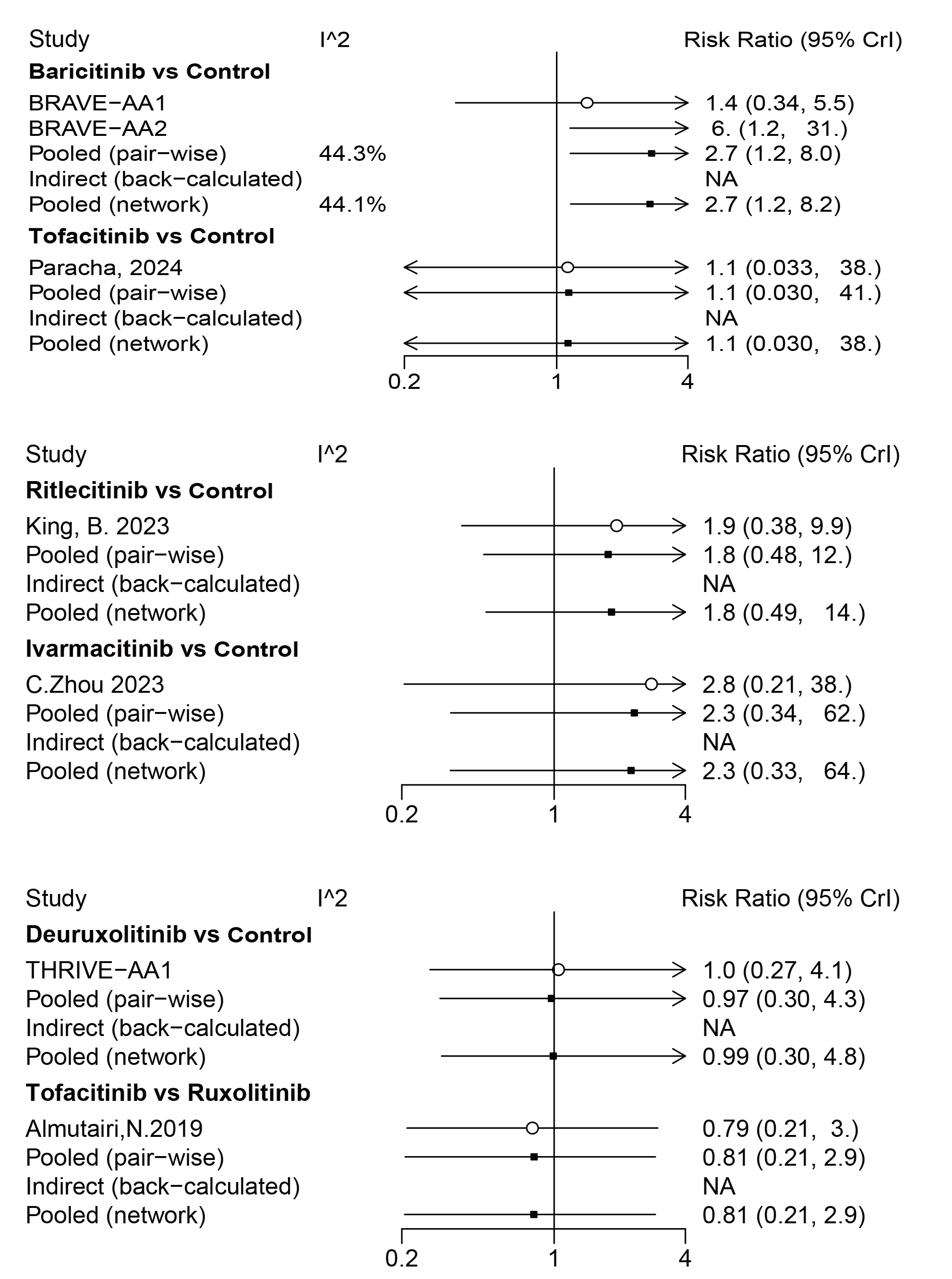


**Figure S7.** Heterogeneity test of Urinary Tract Infection Cases.


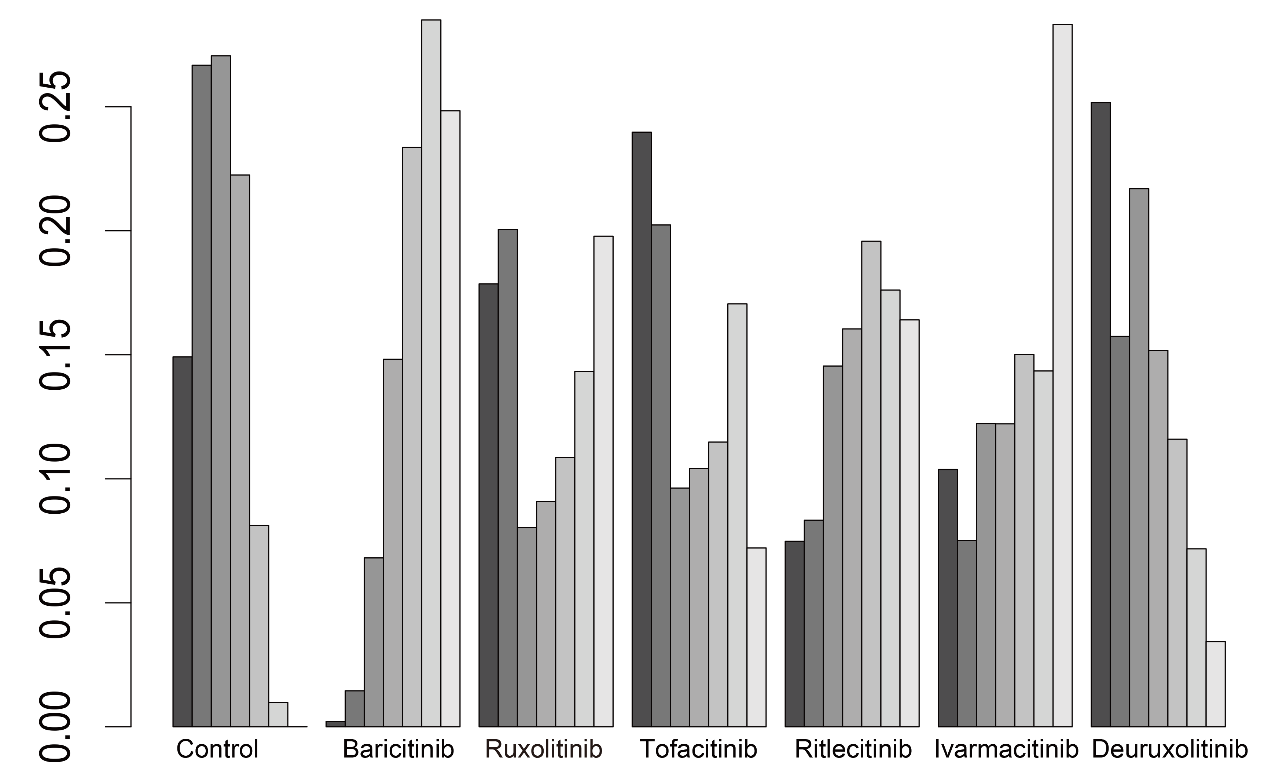


**Figure S8**. Probability Ranking Chart of Urinary Tract Infection.


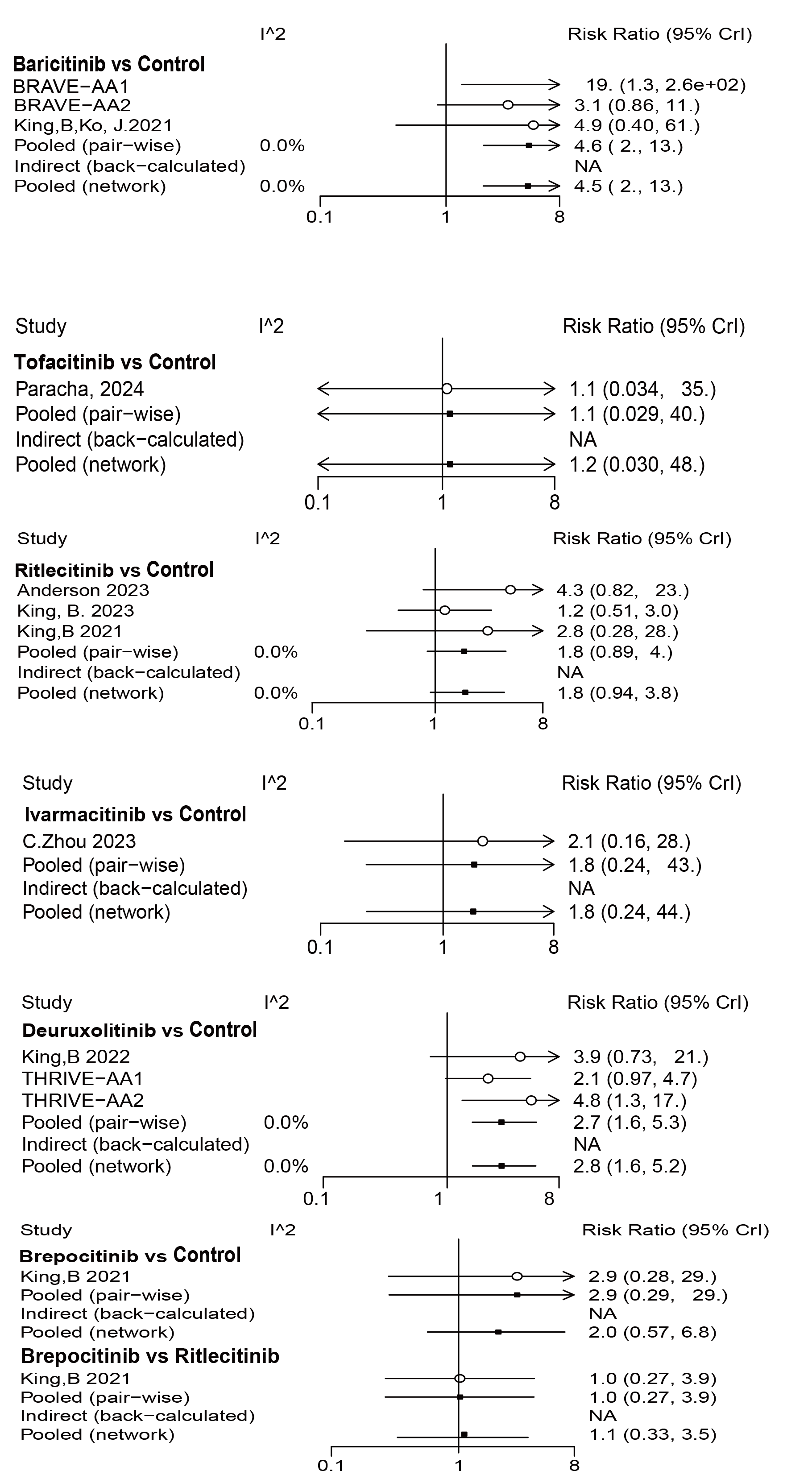


**Figure S9.** Heterogeneity Test of ACNE.


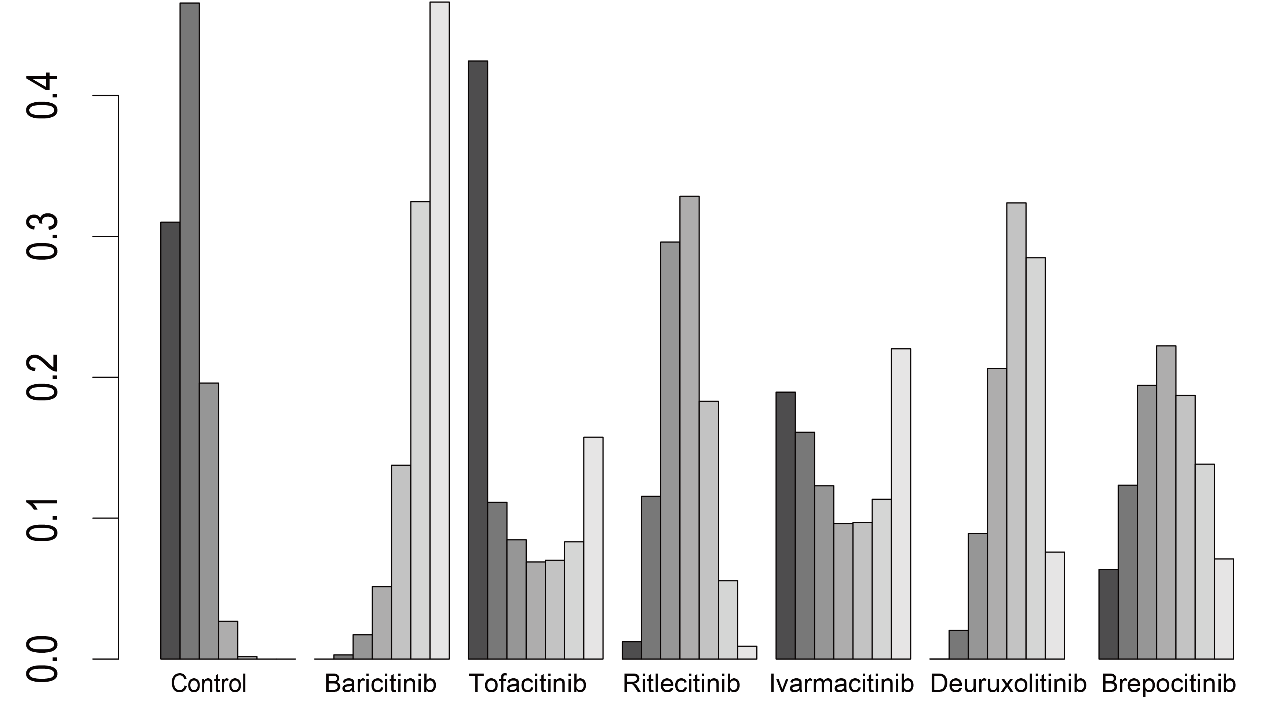


**Figure S10**. Probability Ranking Chart of ACNE.


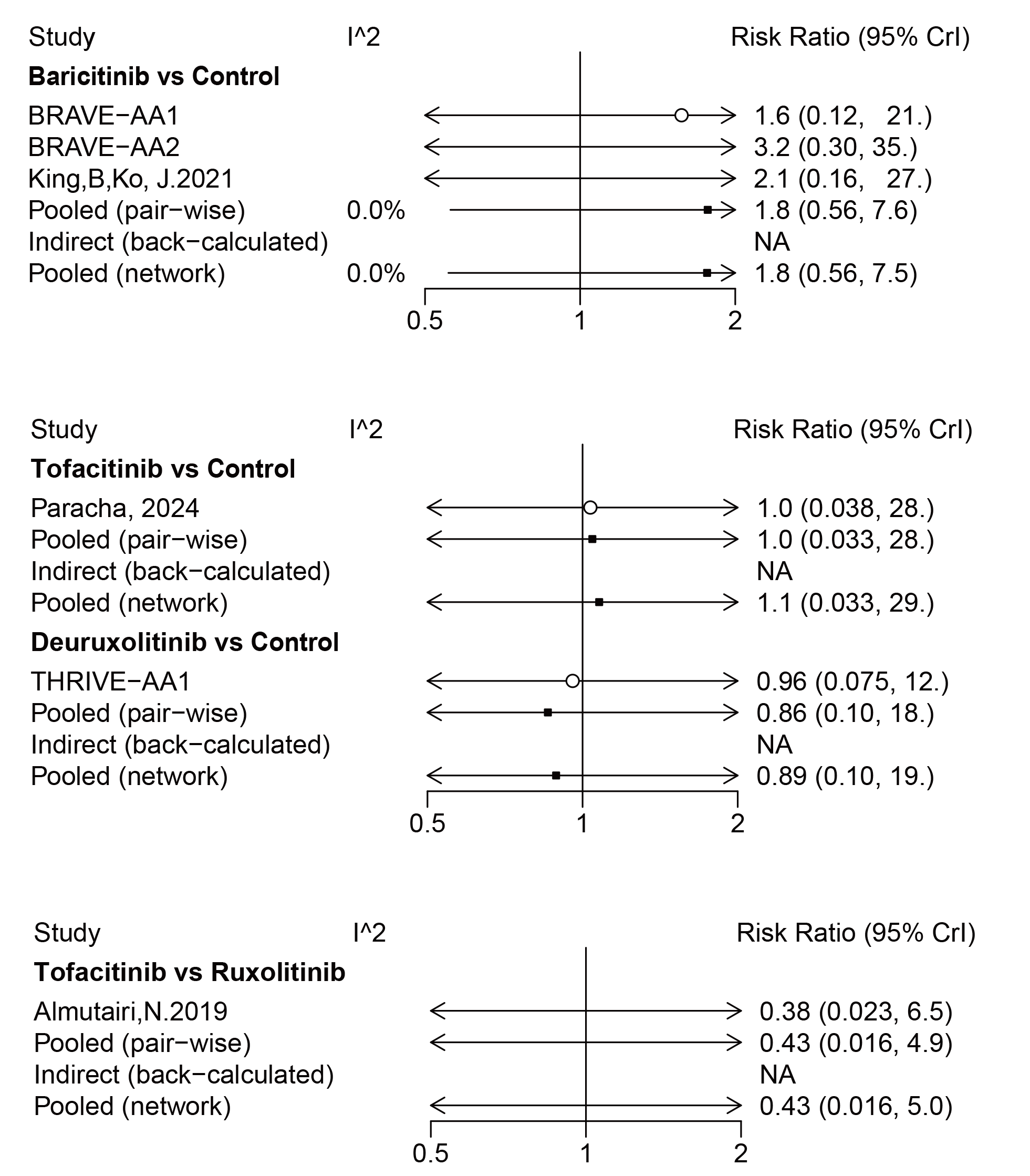


**Figure S11.** Heterogeneity Test of Herpes Zoster.


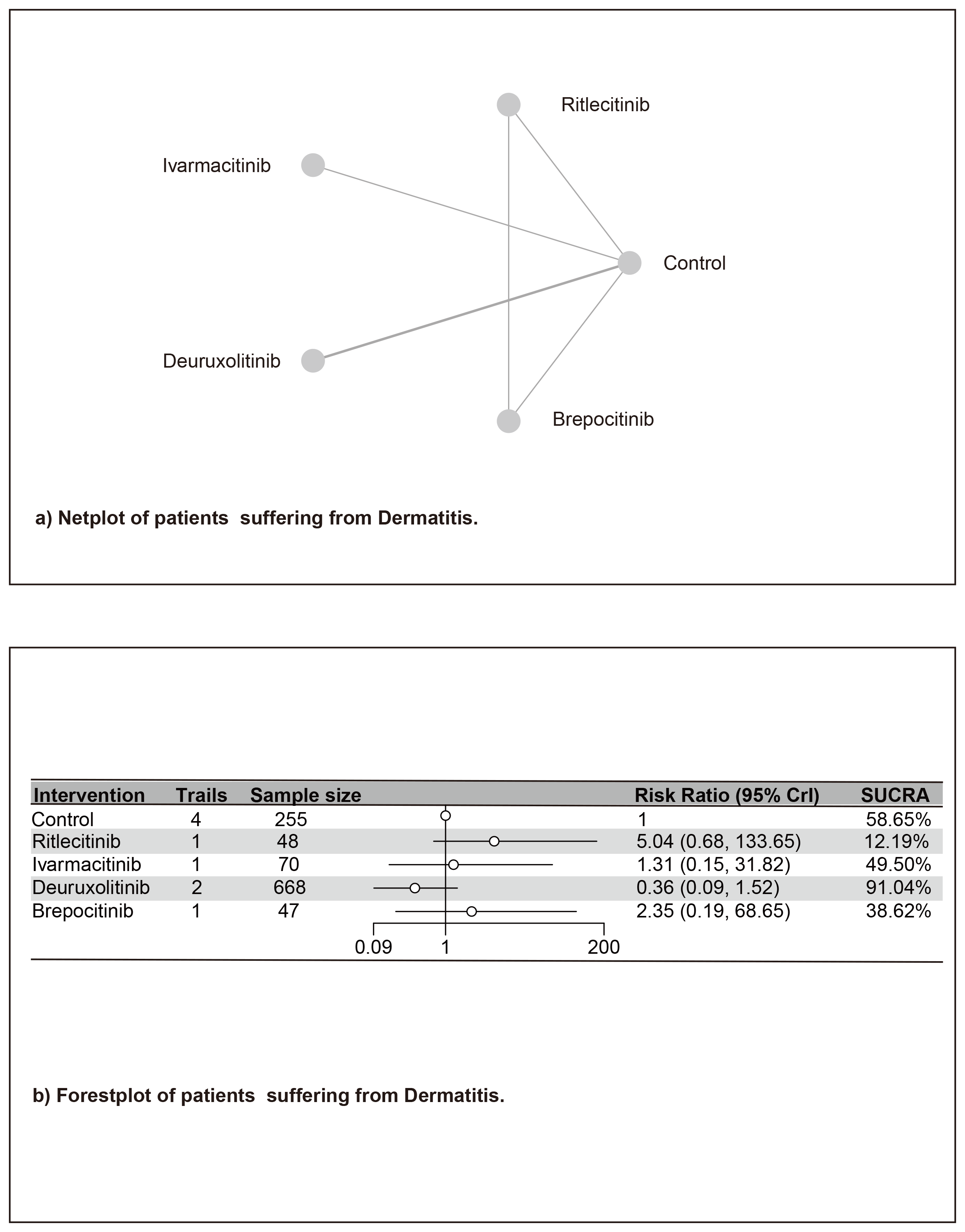


**Figure S12.** Netplot and Forestplot of Patients Suffering from Dermatitis.


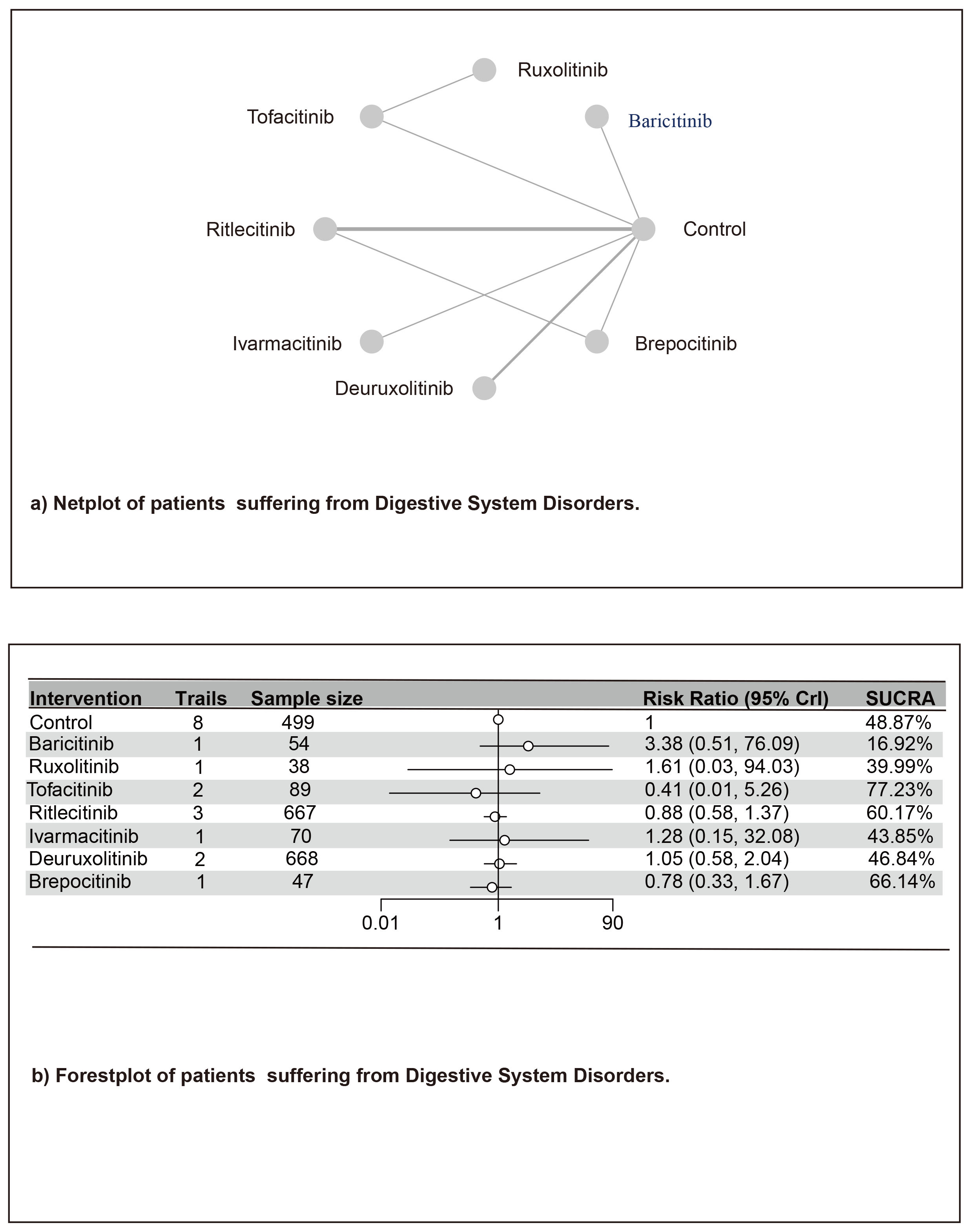


**Figure S13.** Netplot and Forestplot of Patients Suffering from Digestive System Disorders.


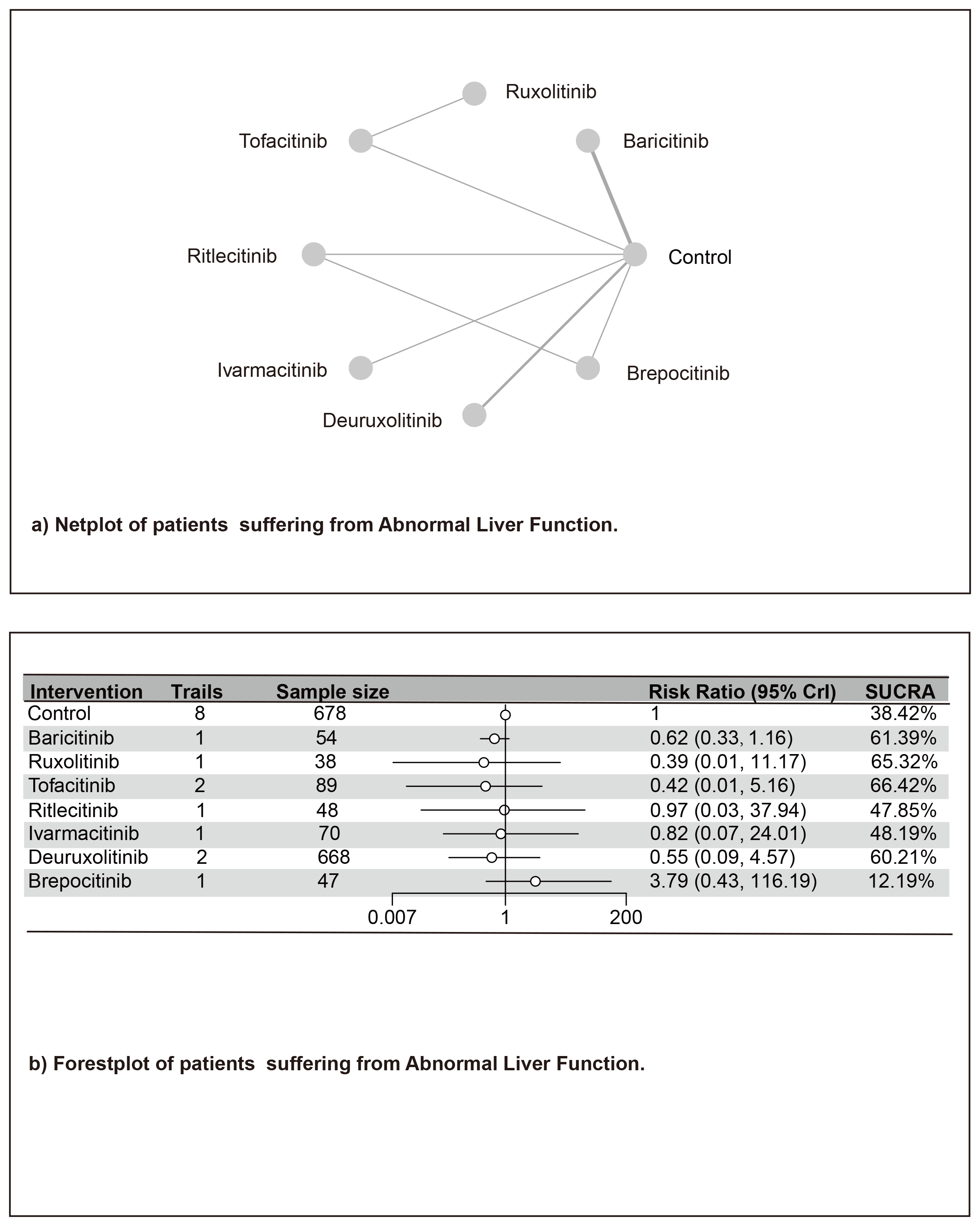


**Figure S14**. Netplot and Forestplot of Patients Suffering from Abnormal Liver Function.


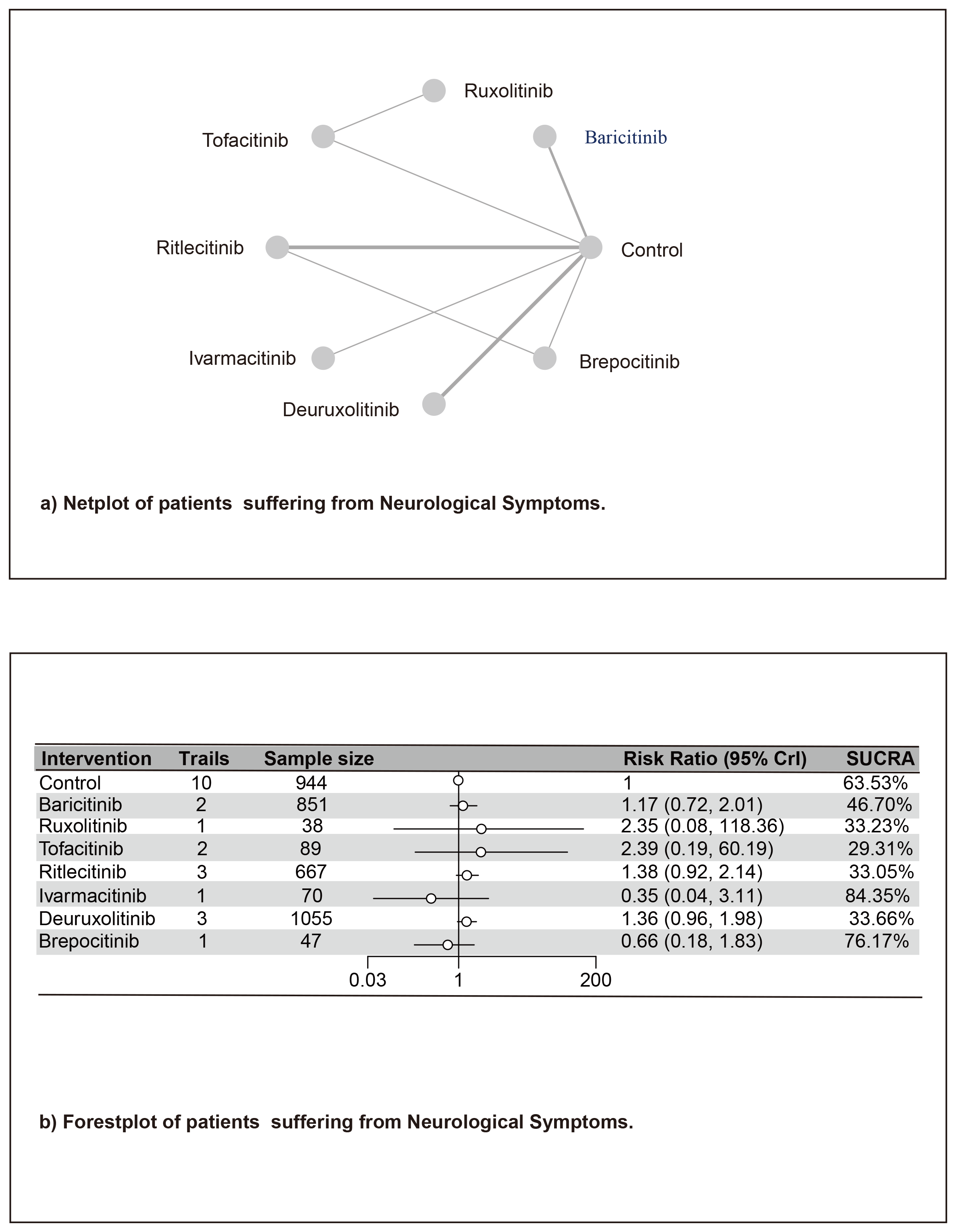


**Figure S15.** Netplot and Forestplot of Patients Suffering from Neurological Symptoms.


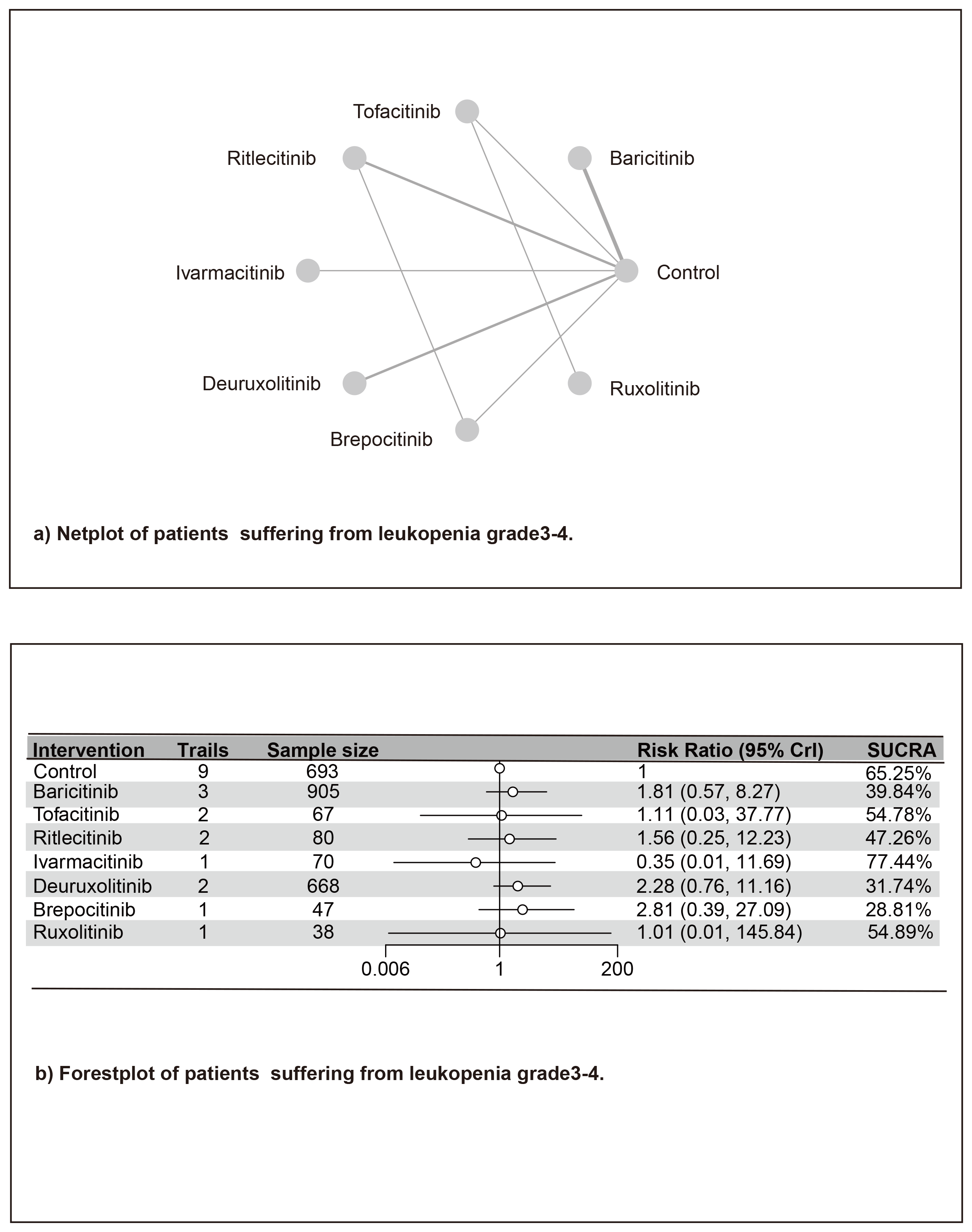


**Figure S16.** Netplot and Forestplot of Patients Suffering from Leukopenia Grade 3-4.


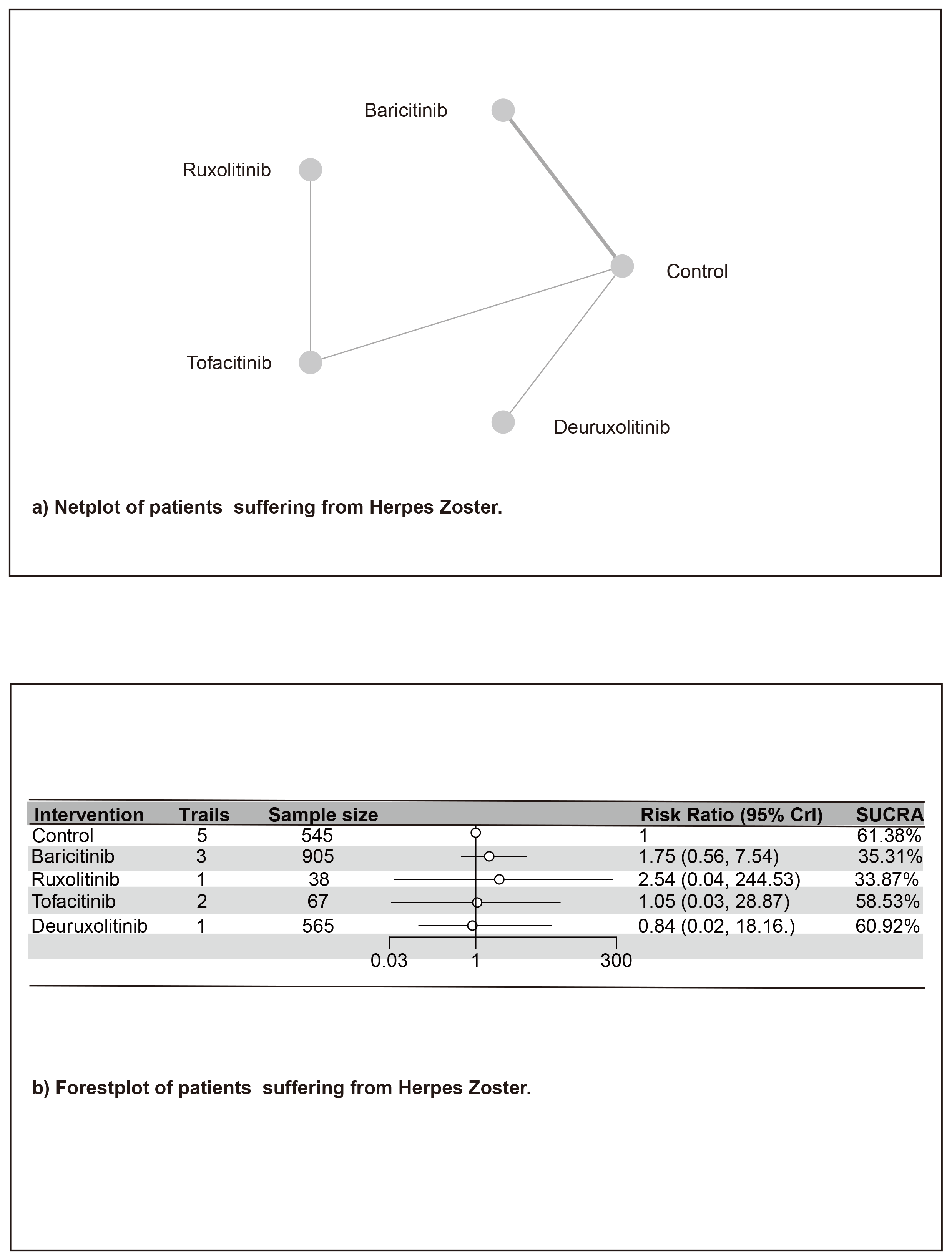


**Figure S17.** Netplot and Forestplot of Patients Suffering from Herpes Zoster.


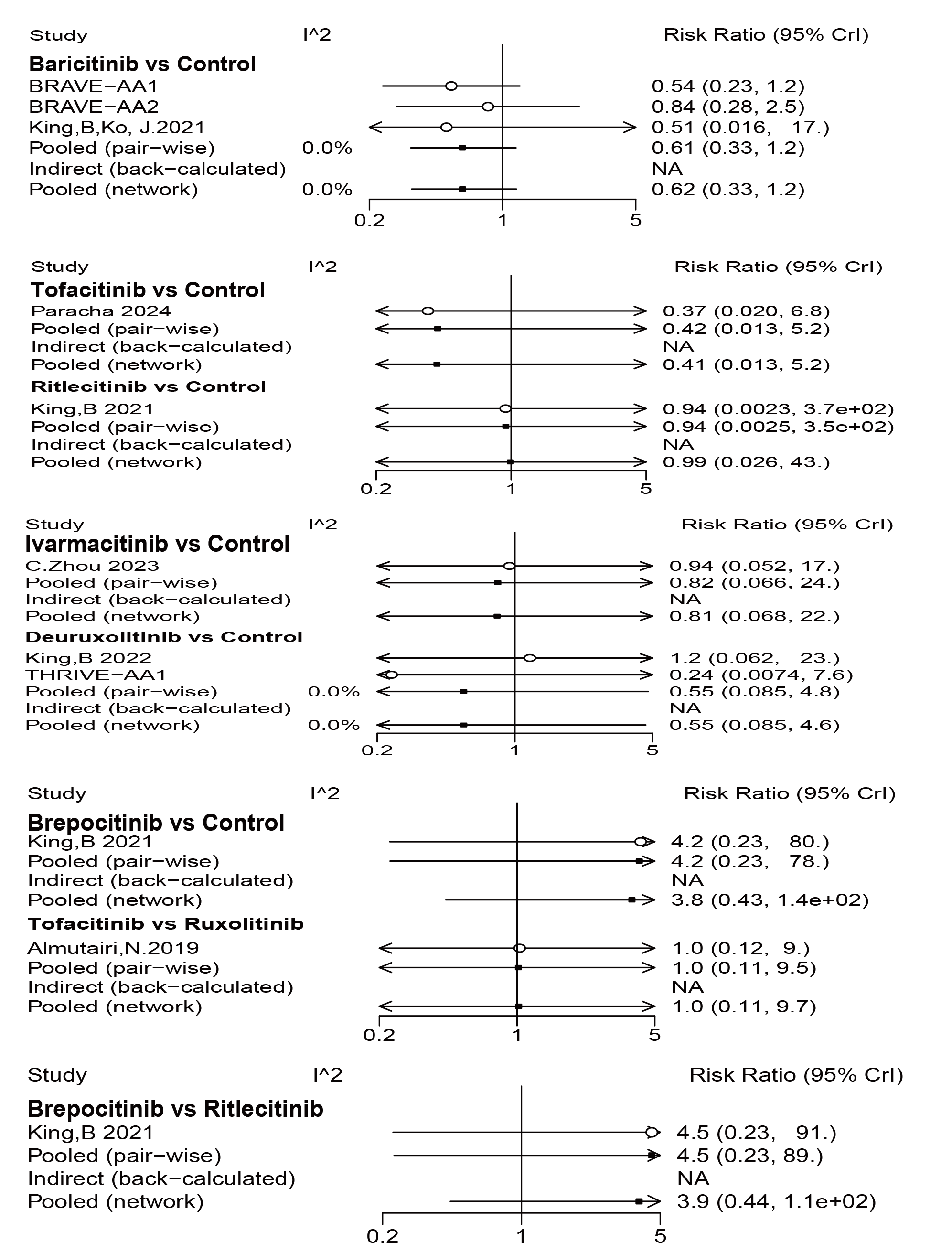


**Figure S18**. Heterogeneity Test of Abnormal Liver Function.


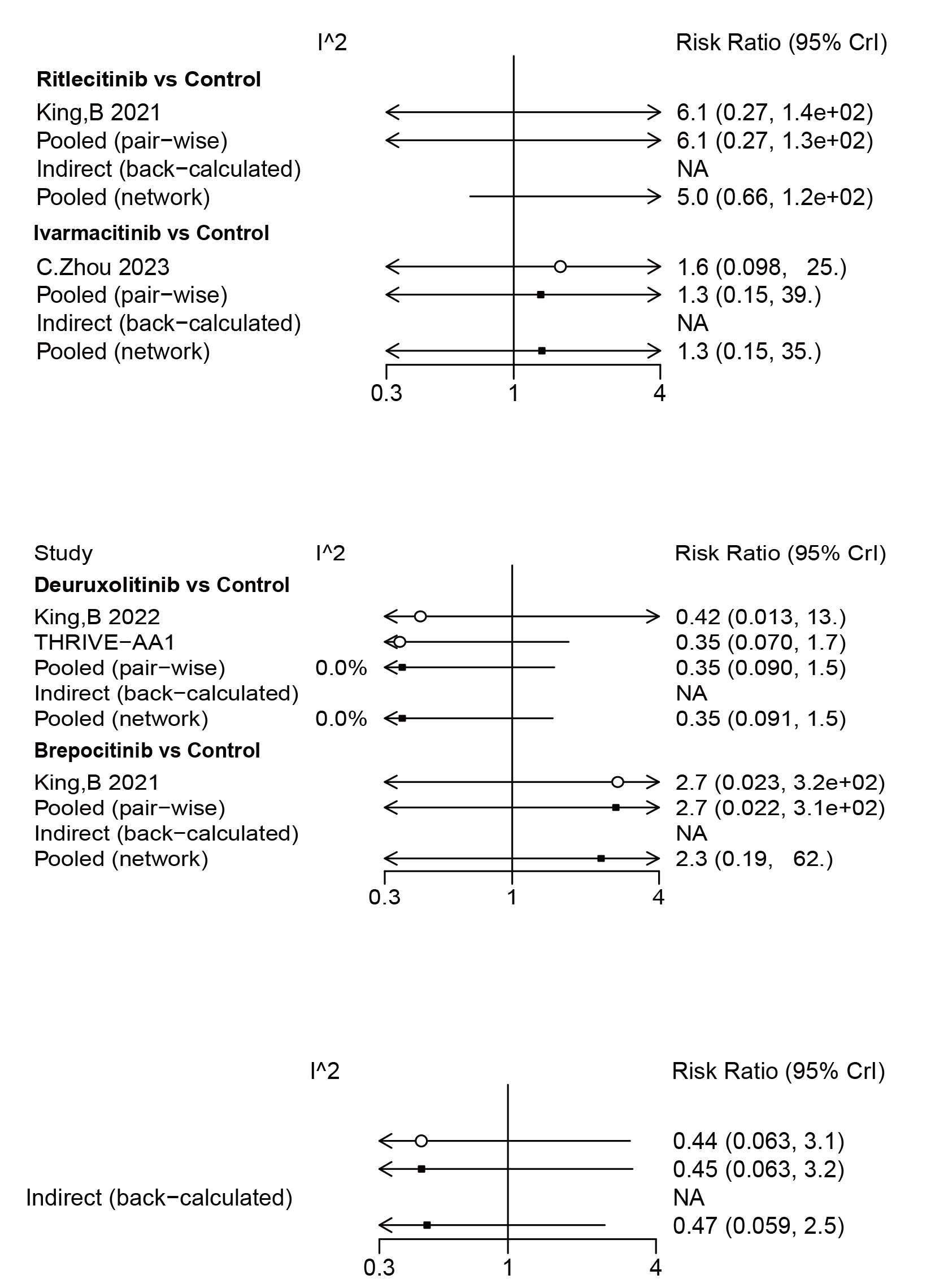


**Figure S19.** Heterogeneity Test of Dermatitis.


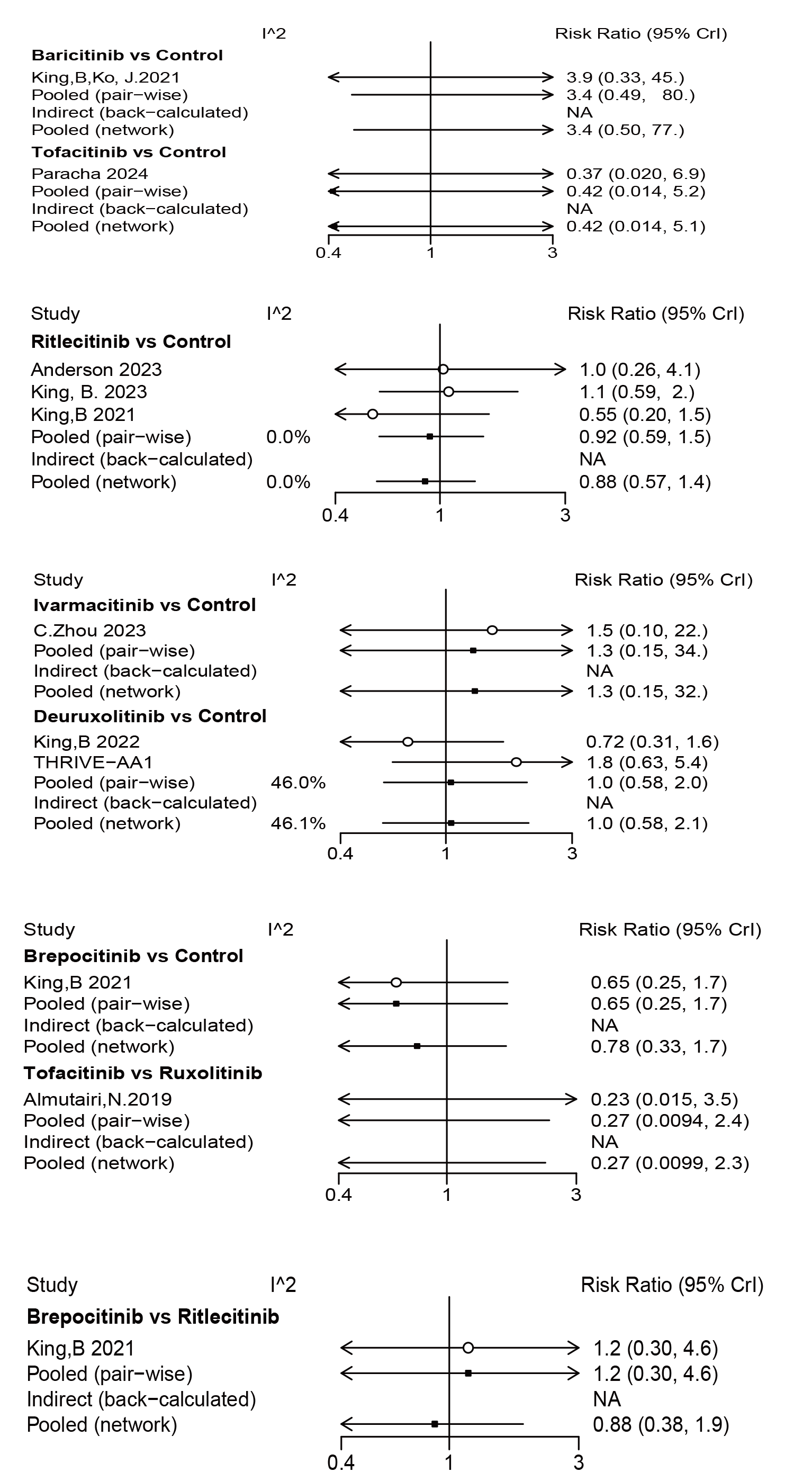


**Figure S20.** Heterogeneity Test of Digestive System Disorders.


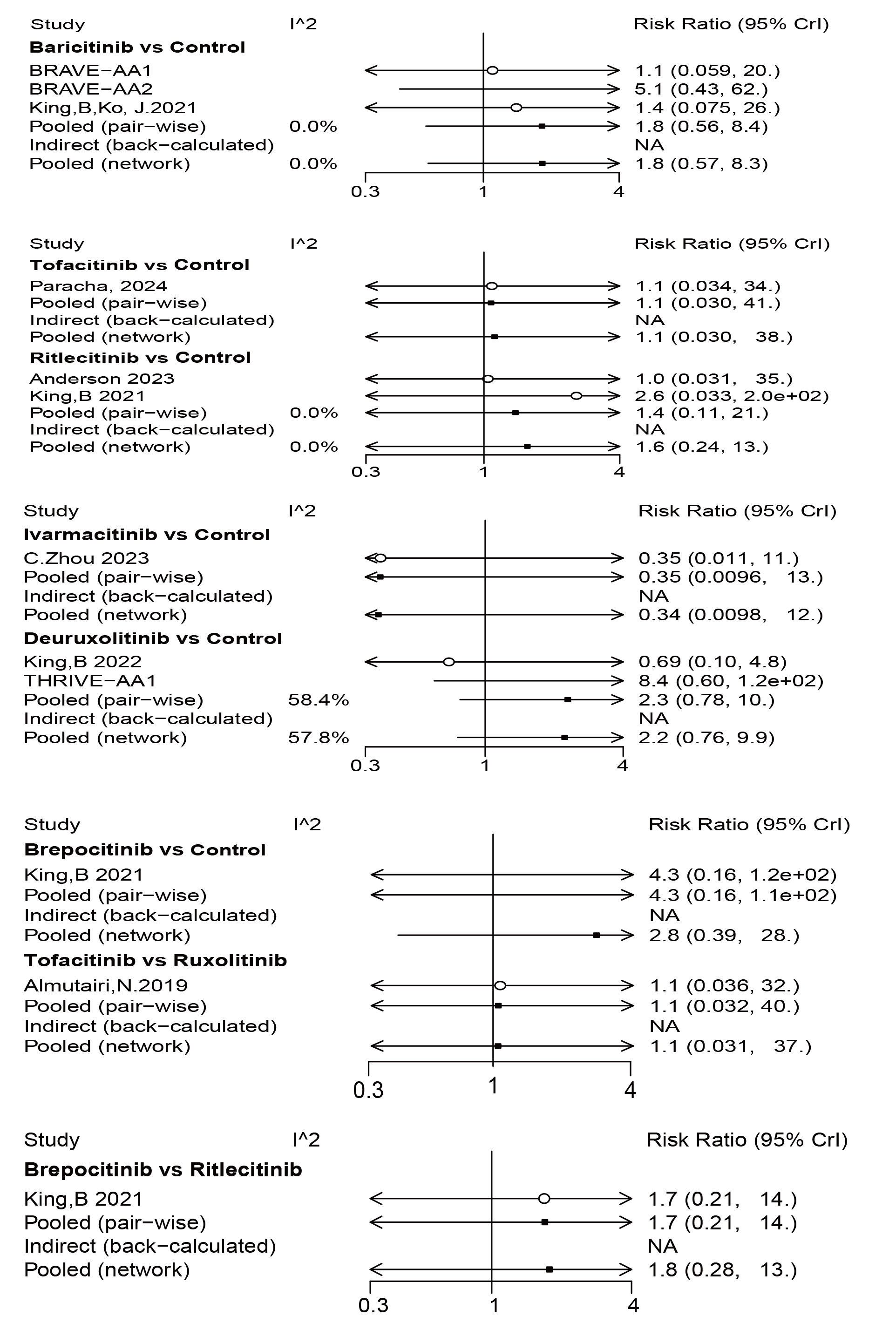


**Figure S21**. Heterogeneity Test of Leukopenia Grade 3-4.


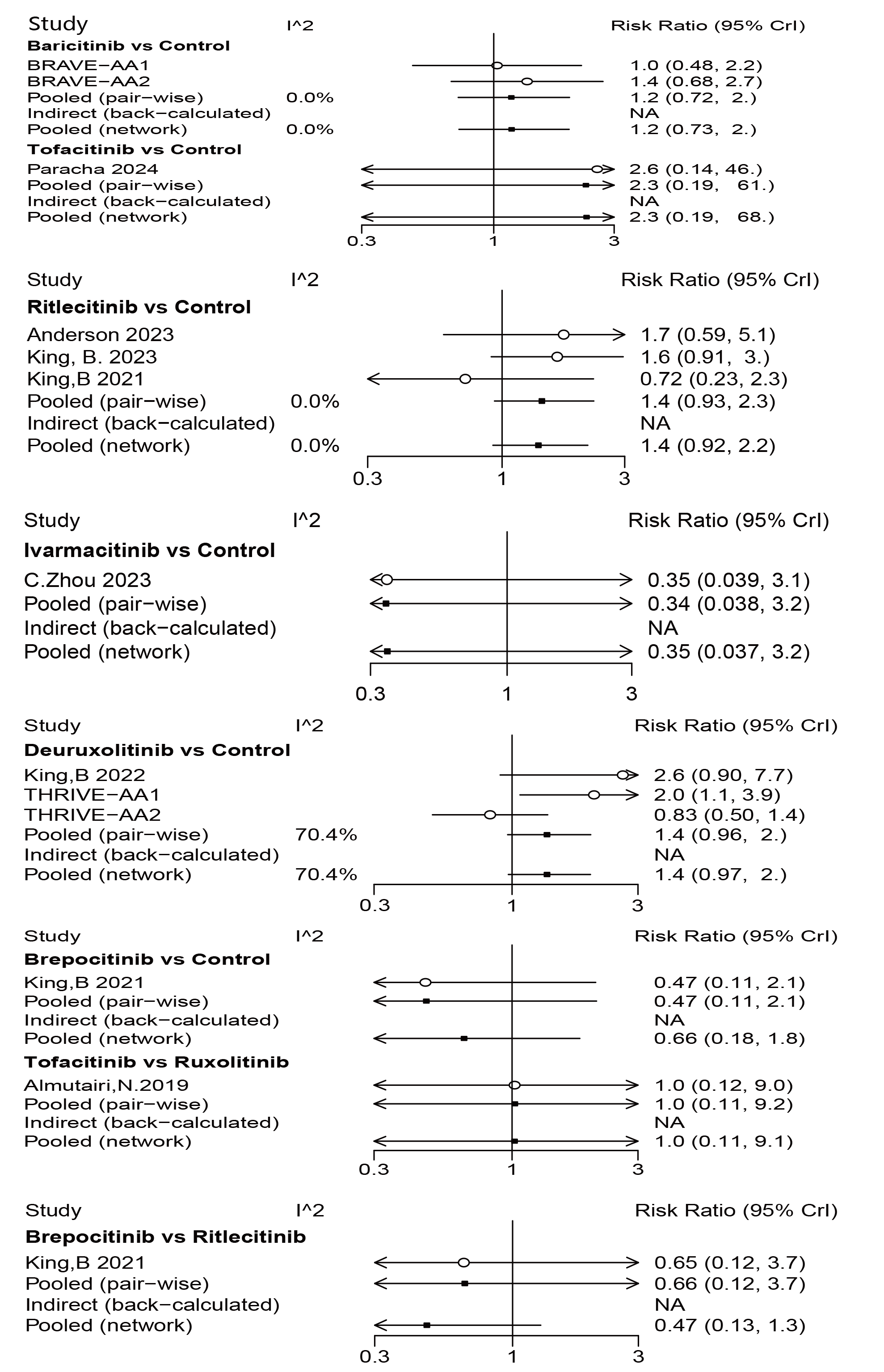


**Figure S22**. Heterogeneity Test of Neurological Symptoms.


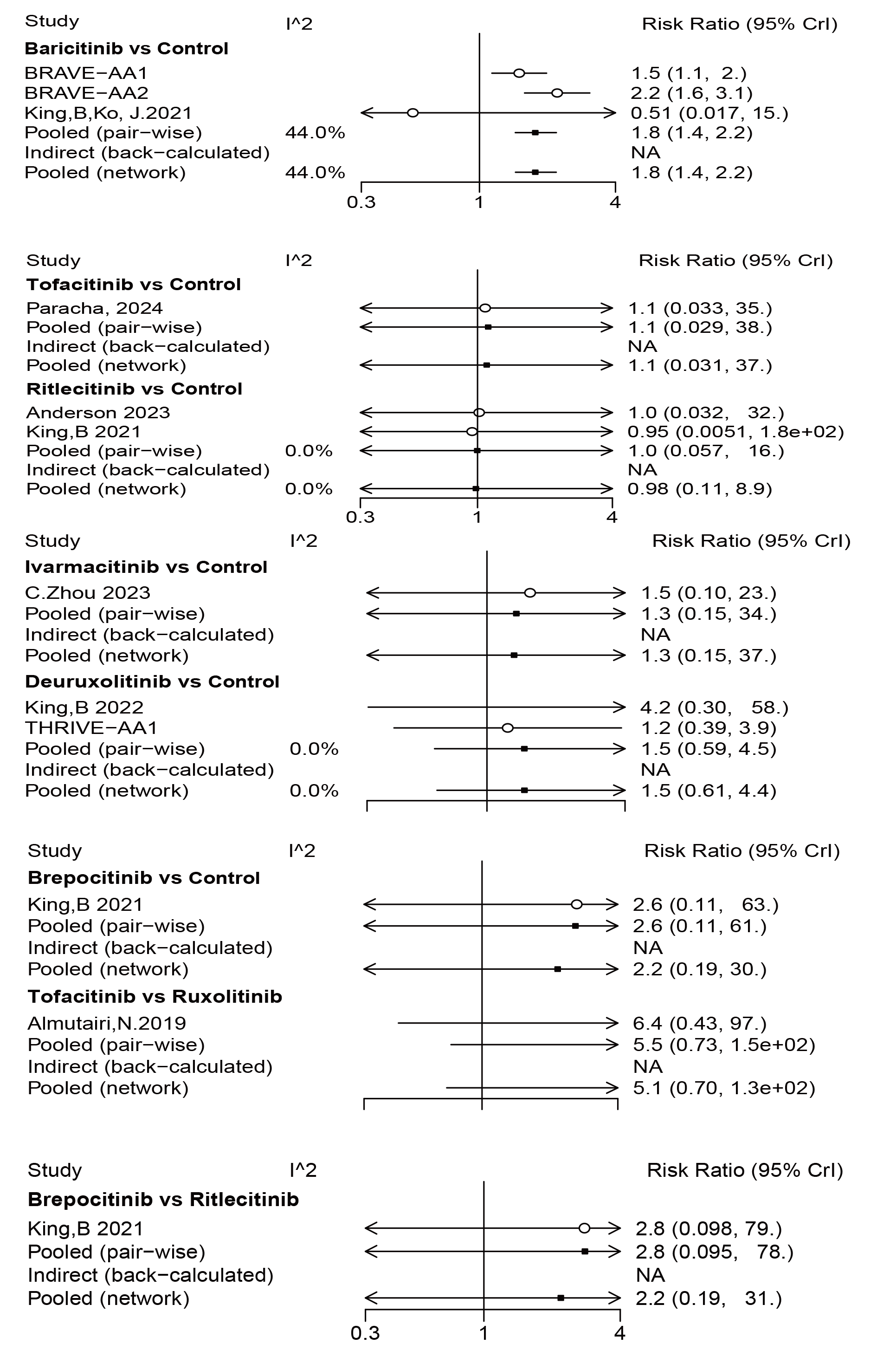


**Figure S23.** Heterogeneity Test of Hyperlipidemia.


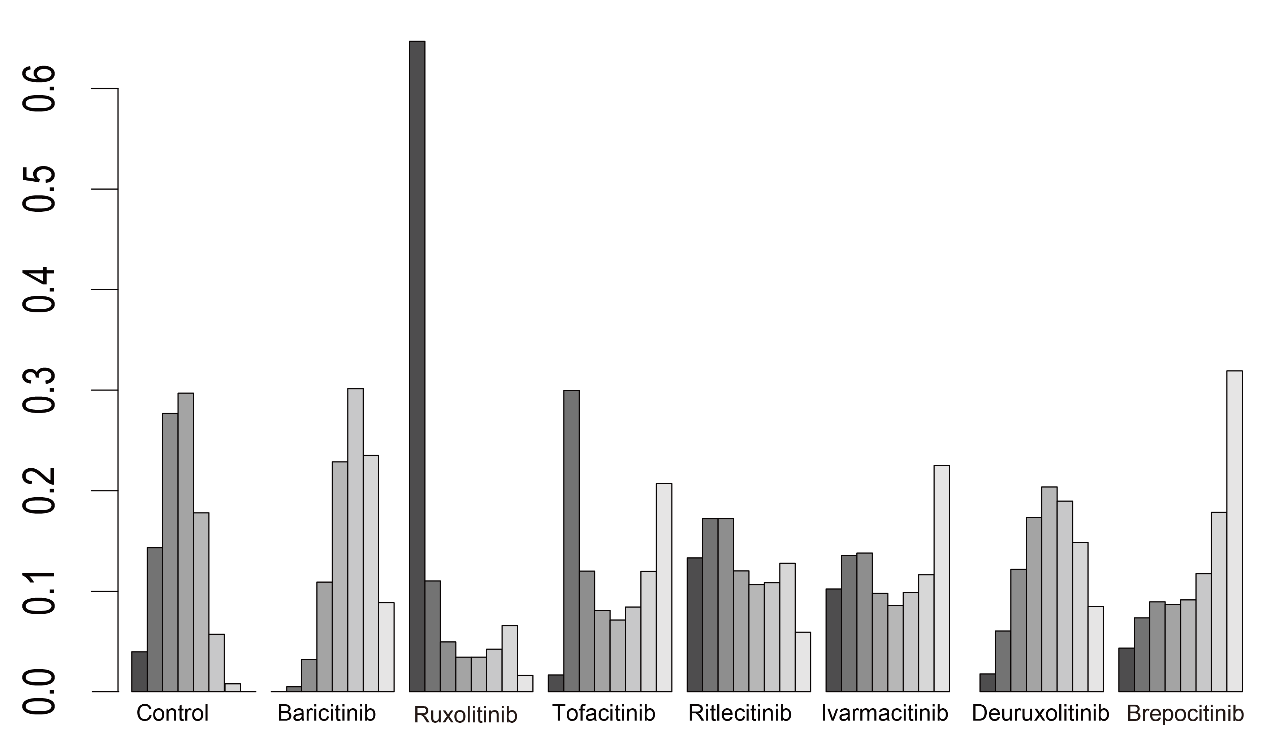


**Figure S24**. Probability Ranking Chart of Hyperlipidemia.


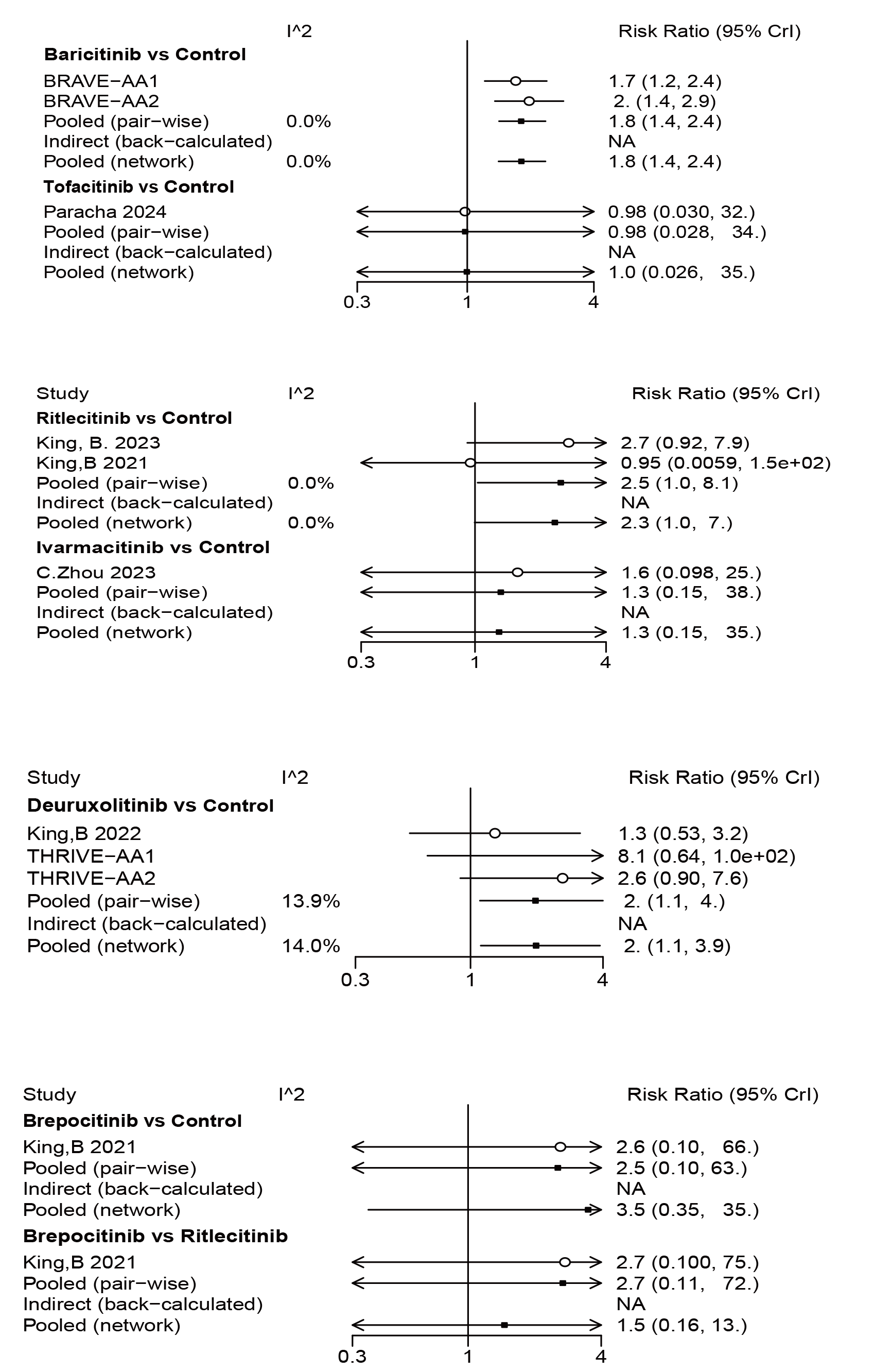


**Figure S25.** Heterogeneity Test of CPK.


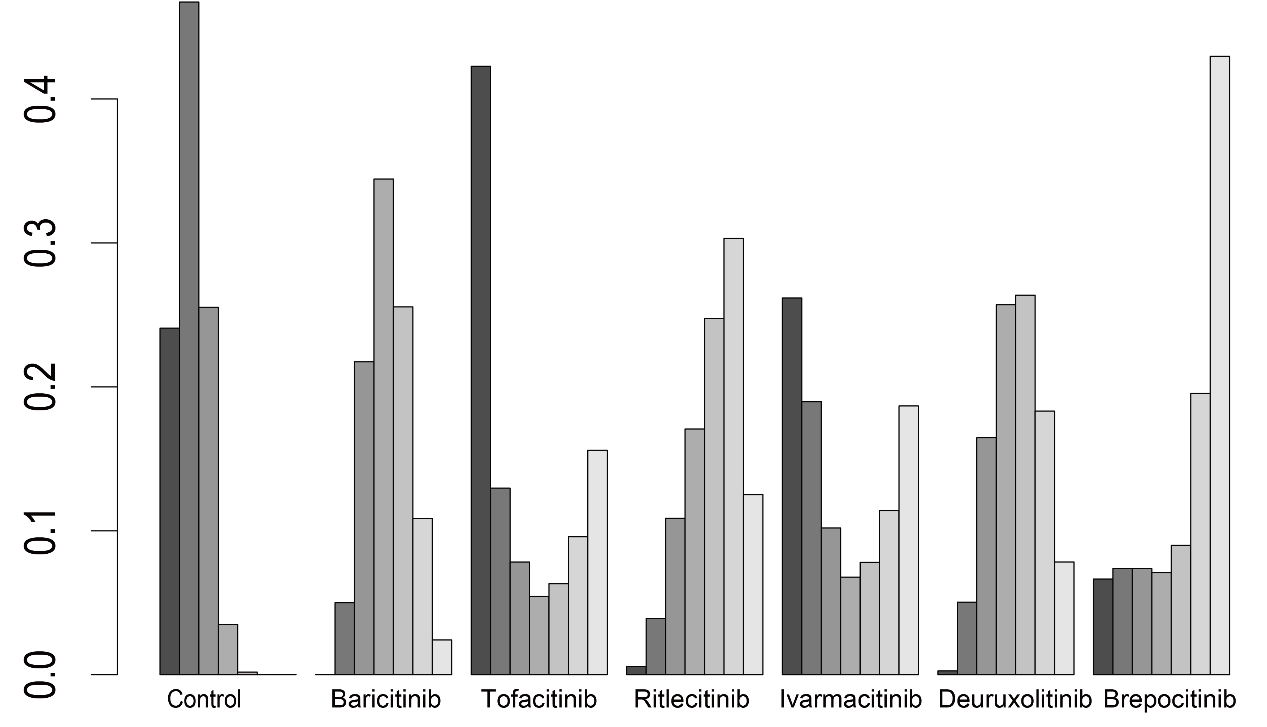


**Figure S26**. Probability Ranking Chart of CPK.


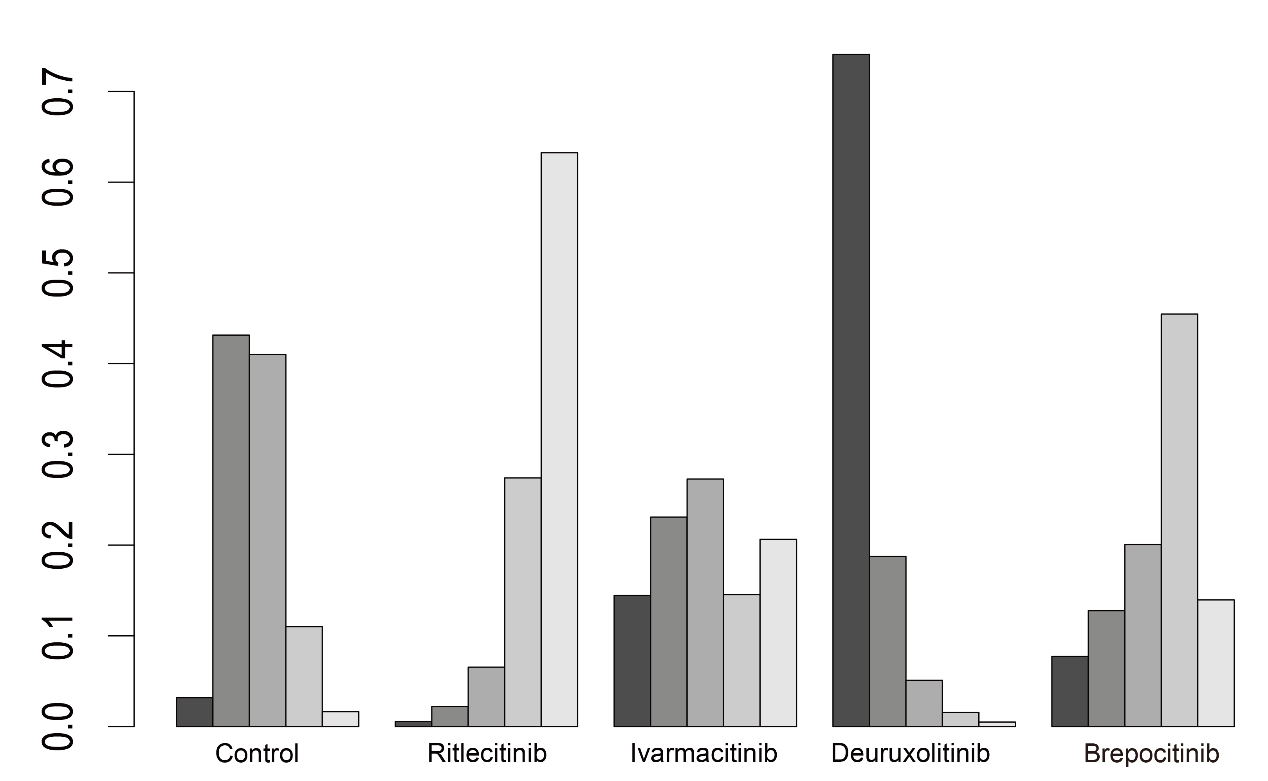


**Figure S27.** Probability Ranking Chart of Dermatitis.


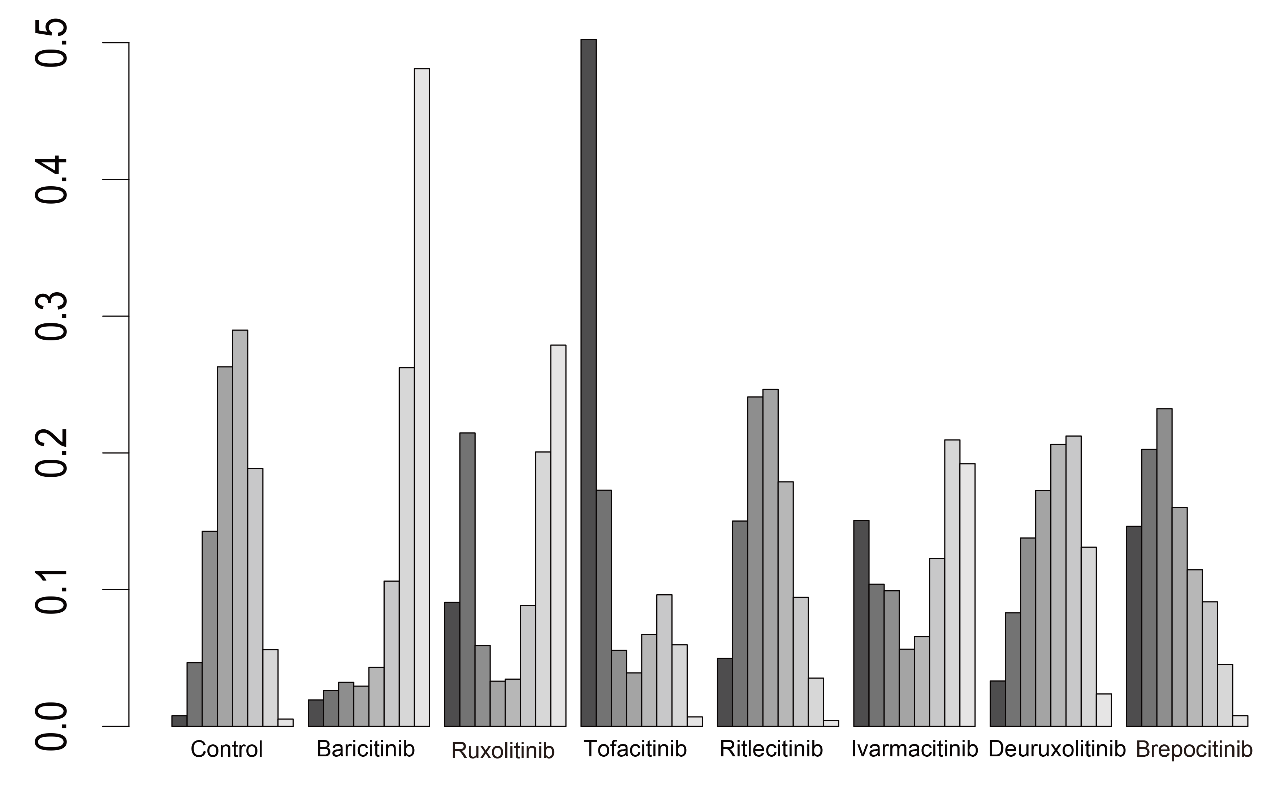


**Figure S28**. Probability Ranking Chart of Digestive System Disorders.


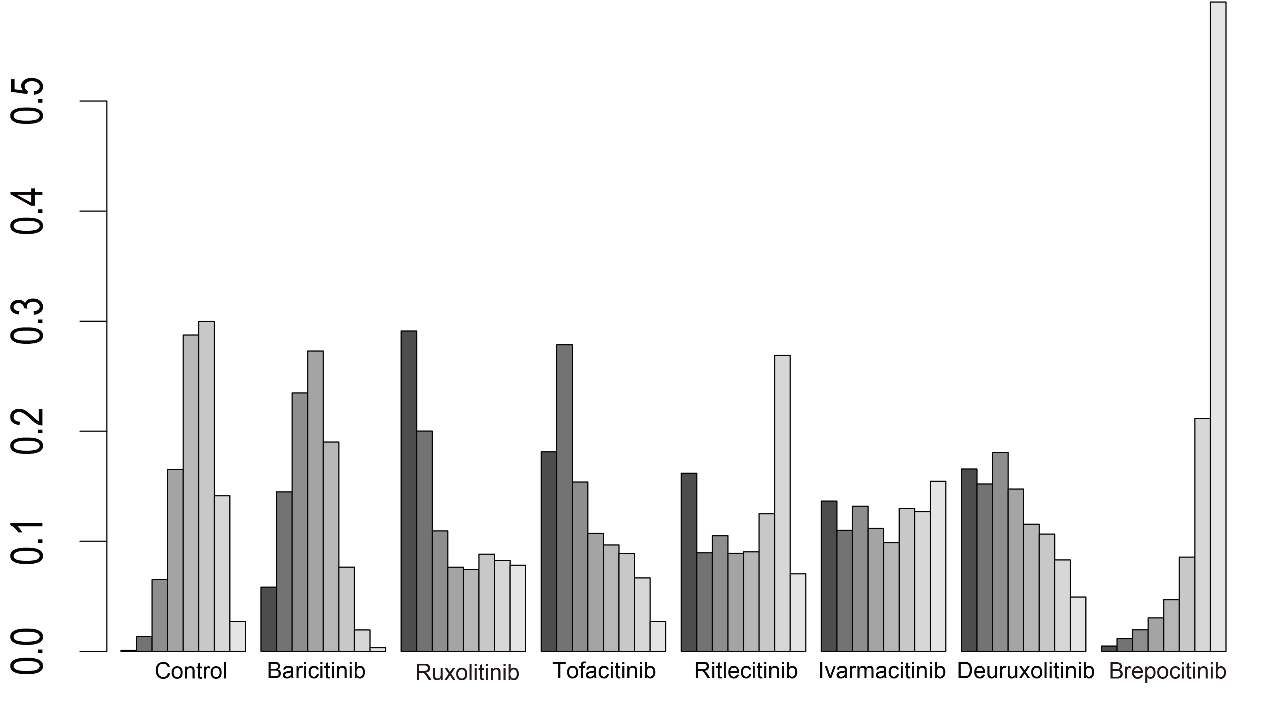


**Figure S29**. Probability Ranking Chart of Abnormal Liver Function.


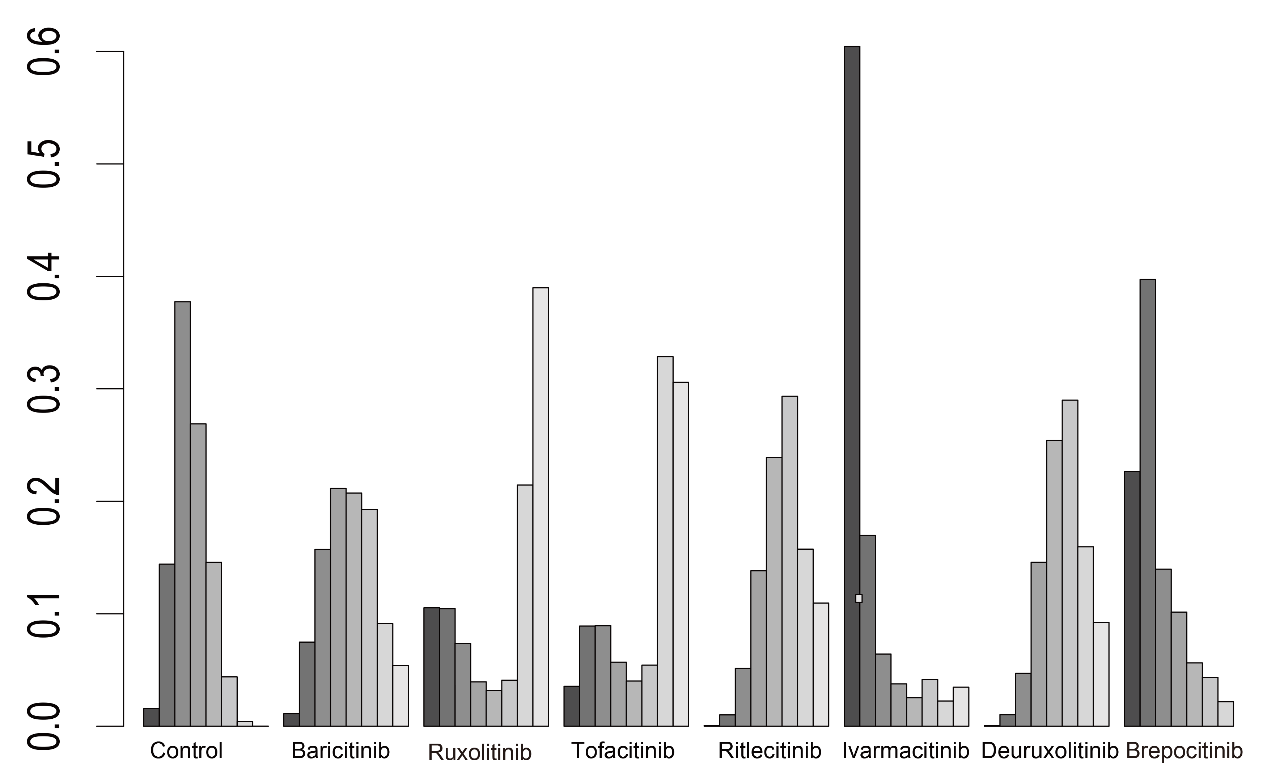


**Figure S30.** Probability Ranking Chart of Neurological Symptoms.


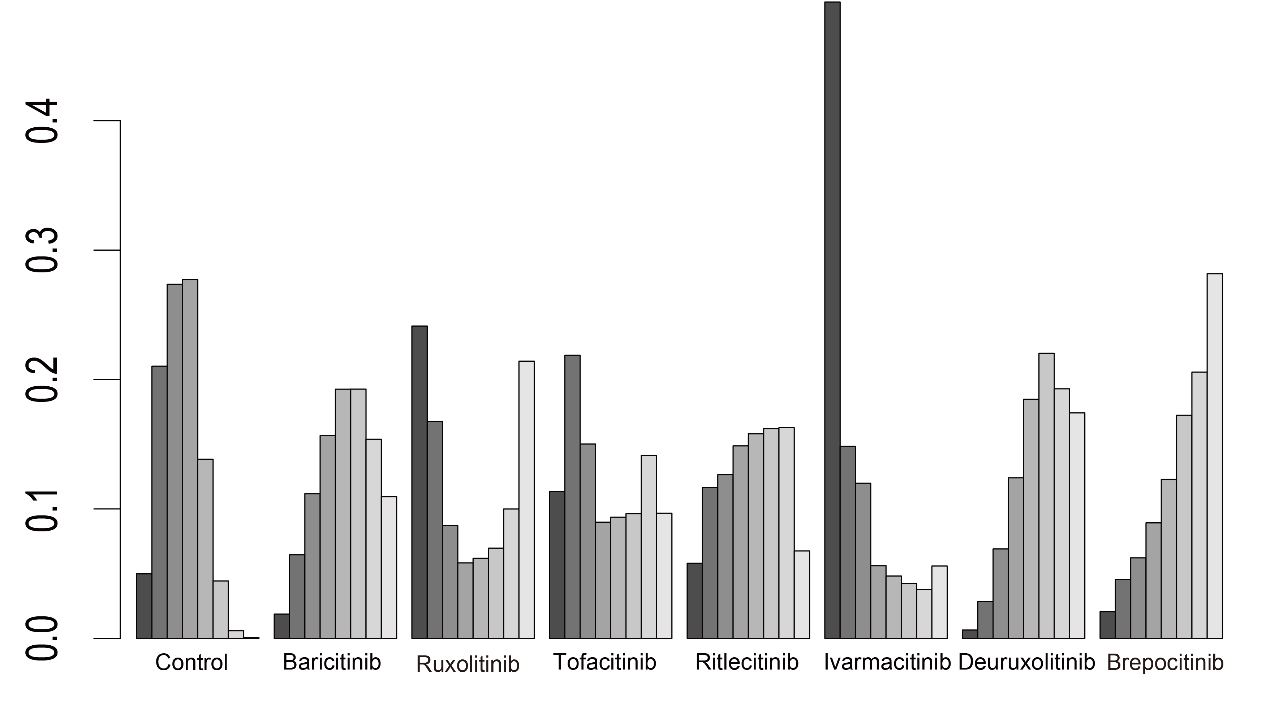


**Figure S31.** Probability Ranking Chart of Leukopenia Grade 3-4.


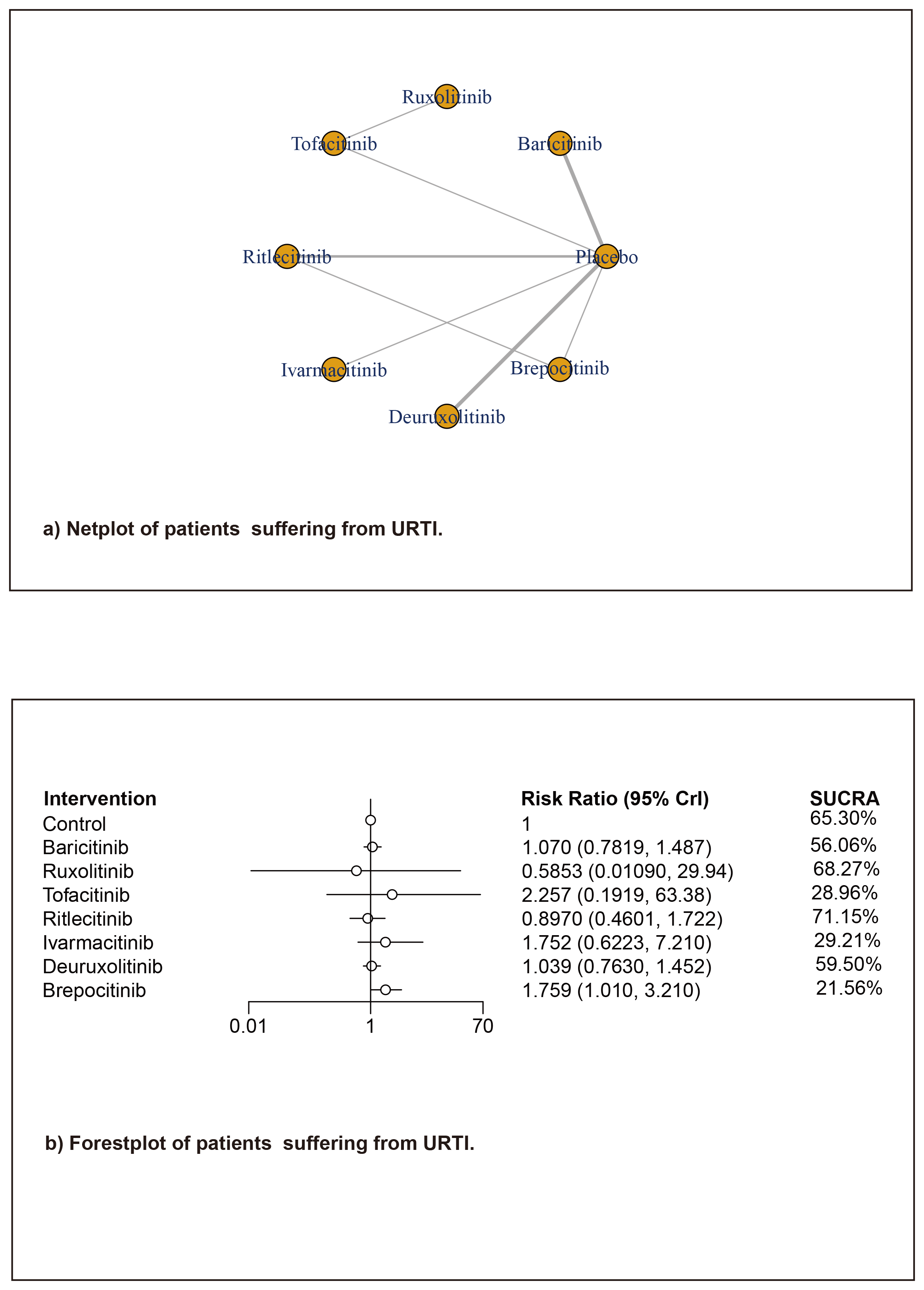


**Figure S32.** Netplot and forestplot of patients suffering from URTI.


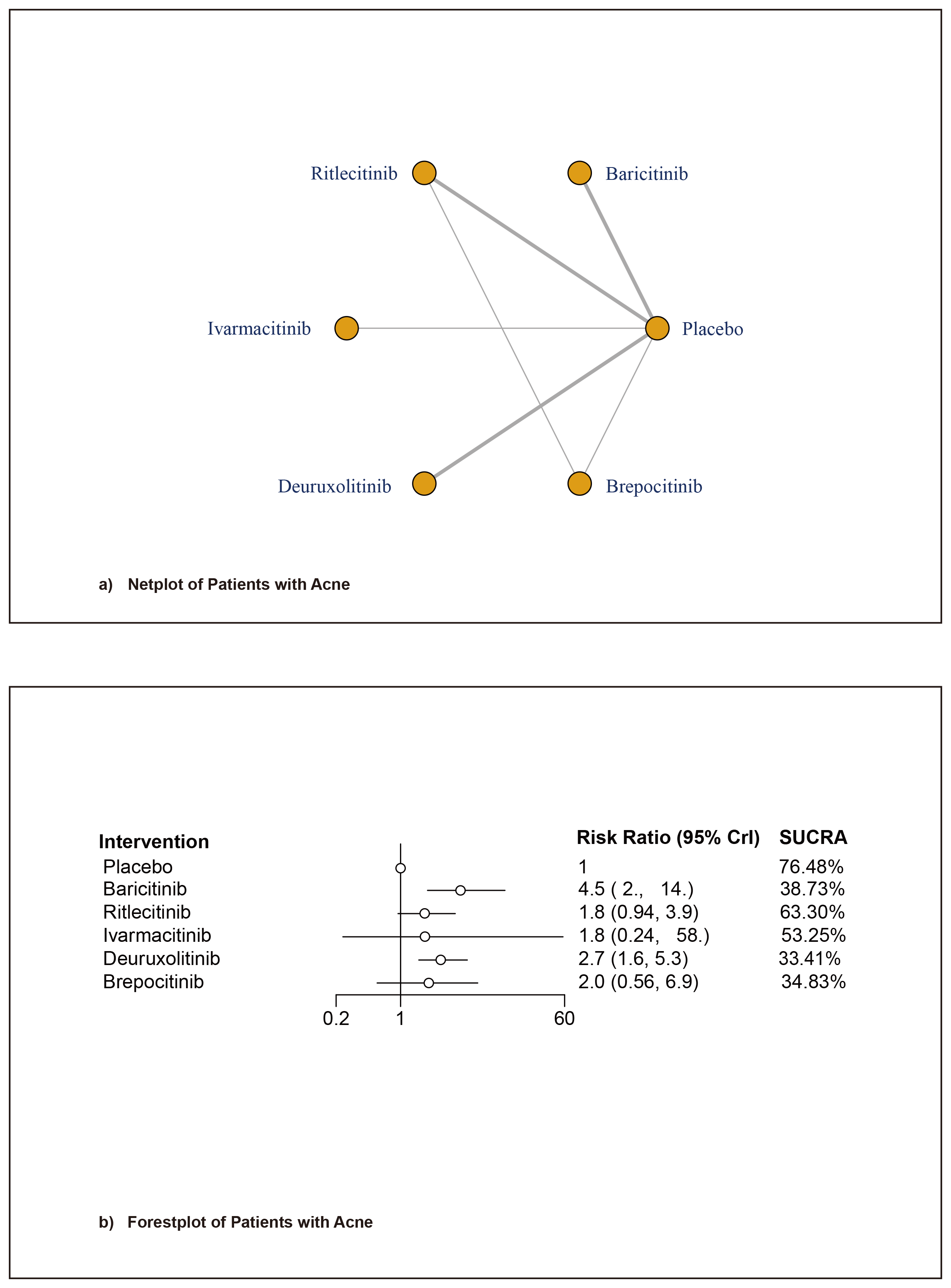


**Figure S33.** Netplot and forestplot of patients suffering from Acne

**
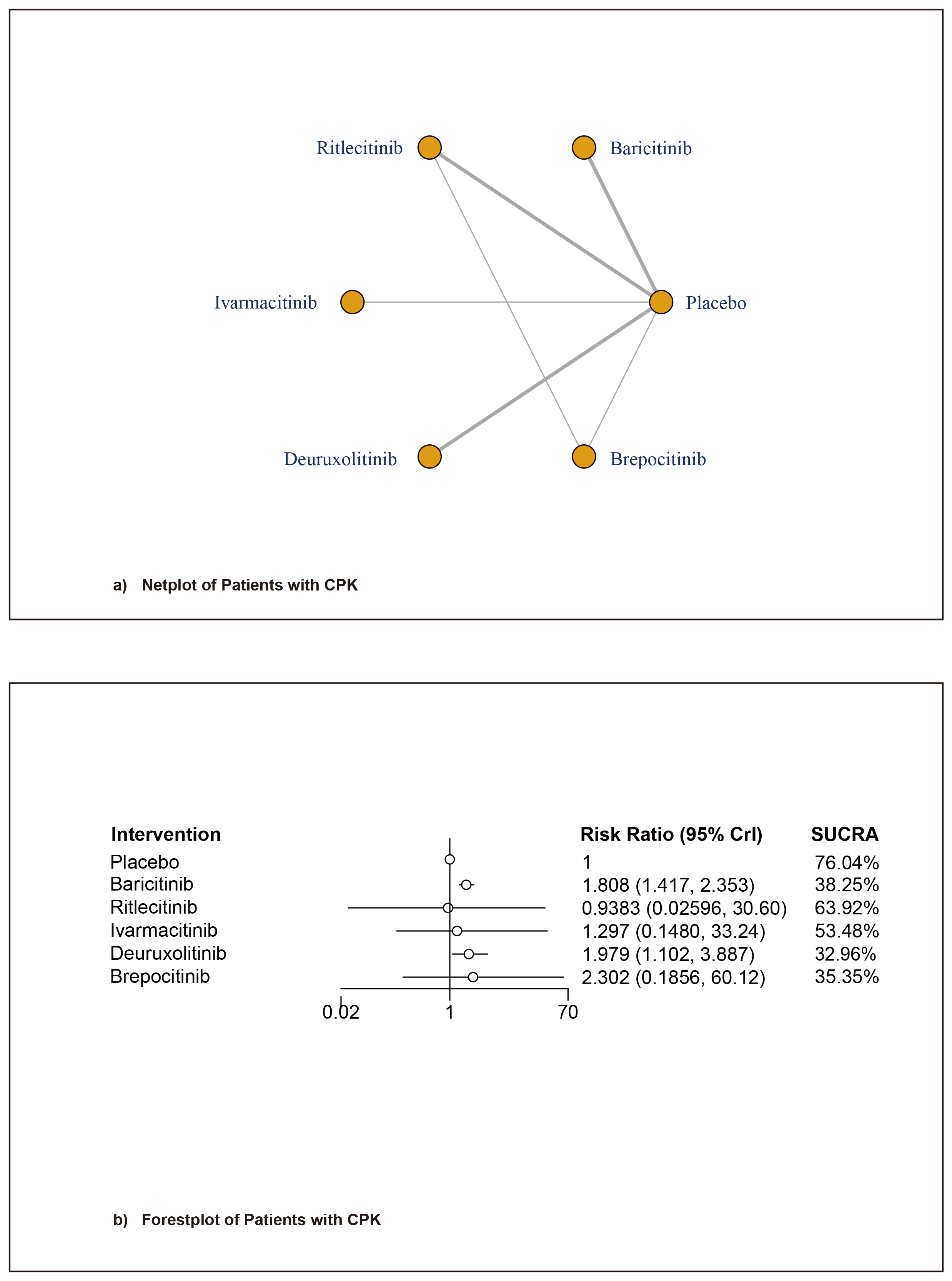
Figure S34.** Netplot and forestplot of patients suffering from CPK

**
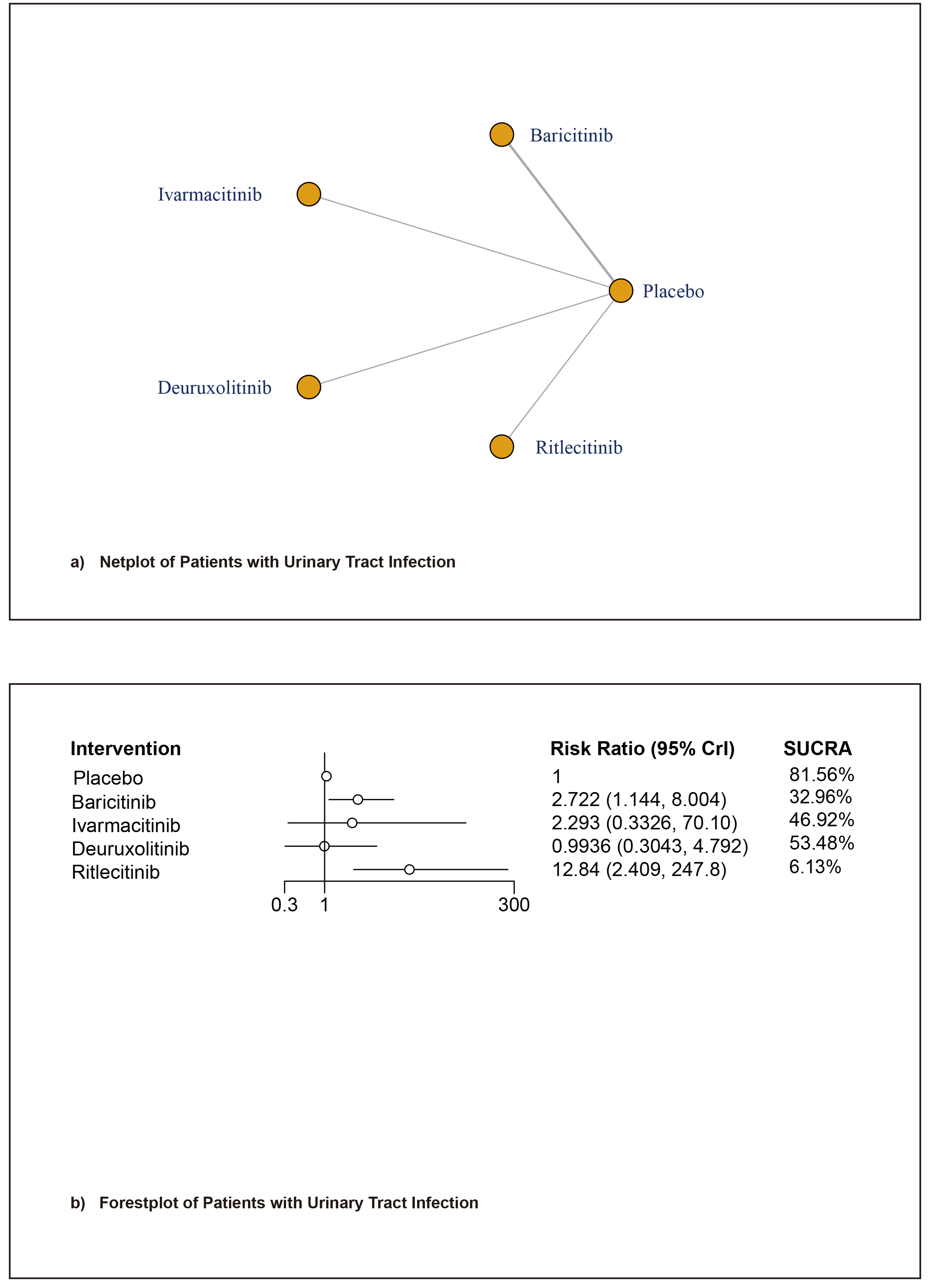
Figure S35.** Netplot and forestplot of patients suffering from Urinary Tract Infection

**
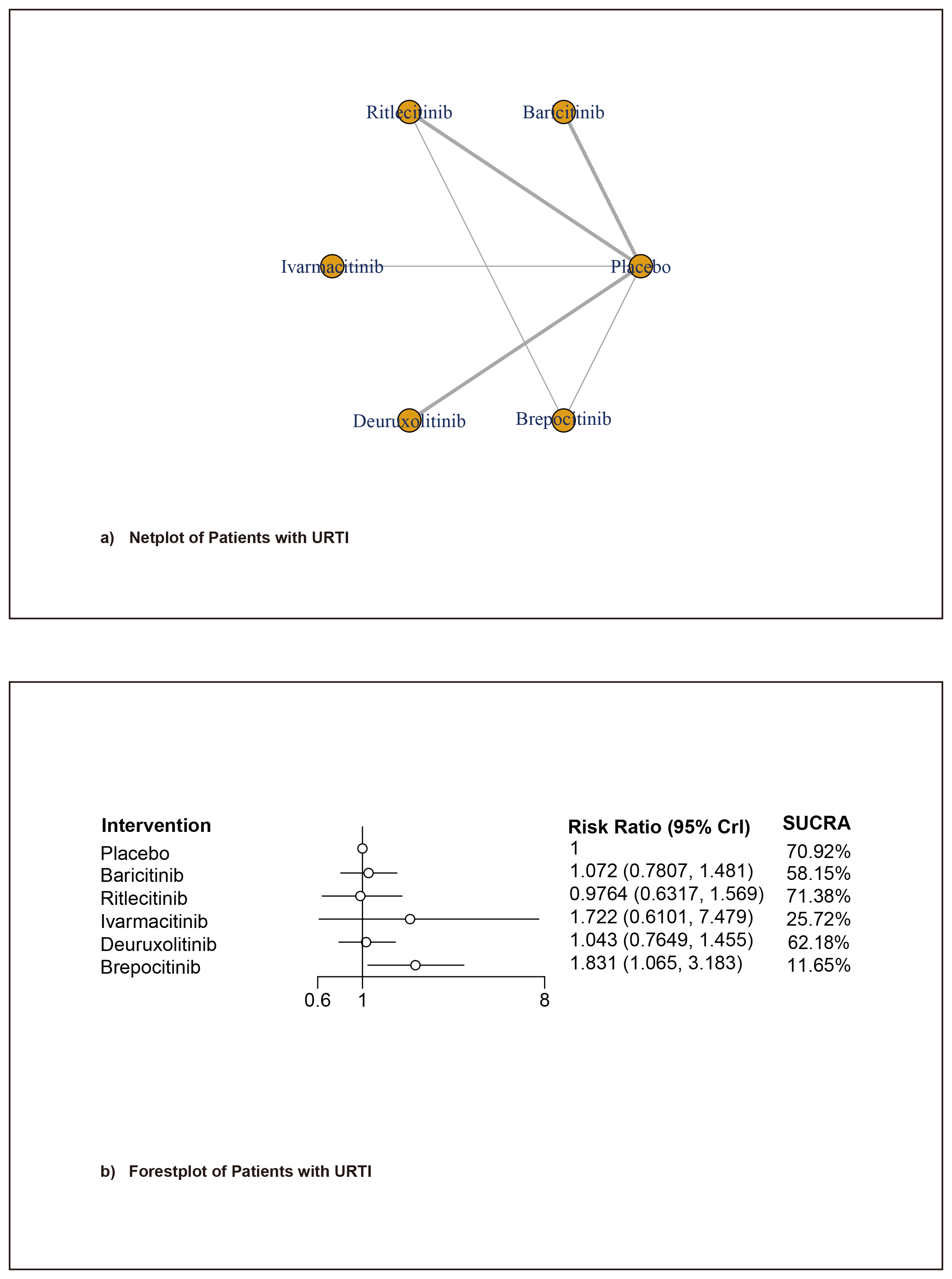
Figure S36.** Netplot and forestplot of patients suffering from URTI

**
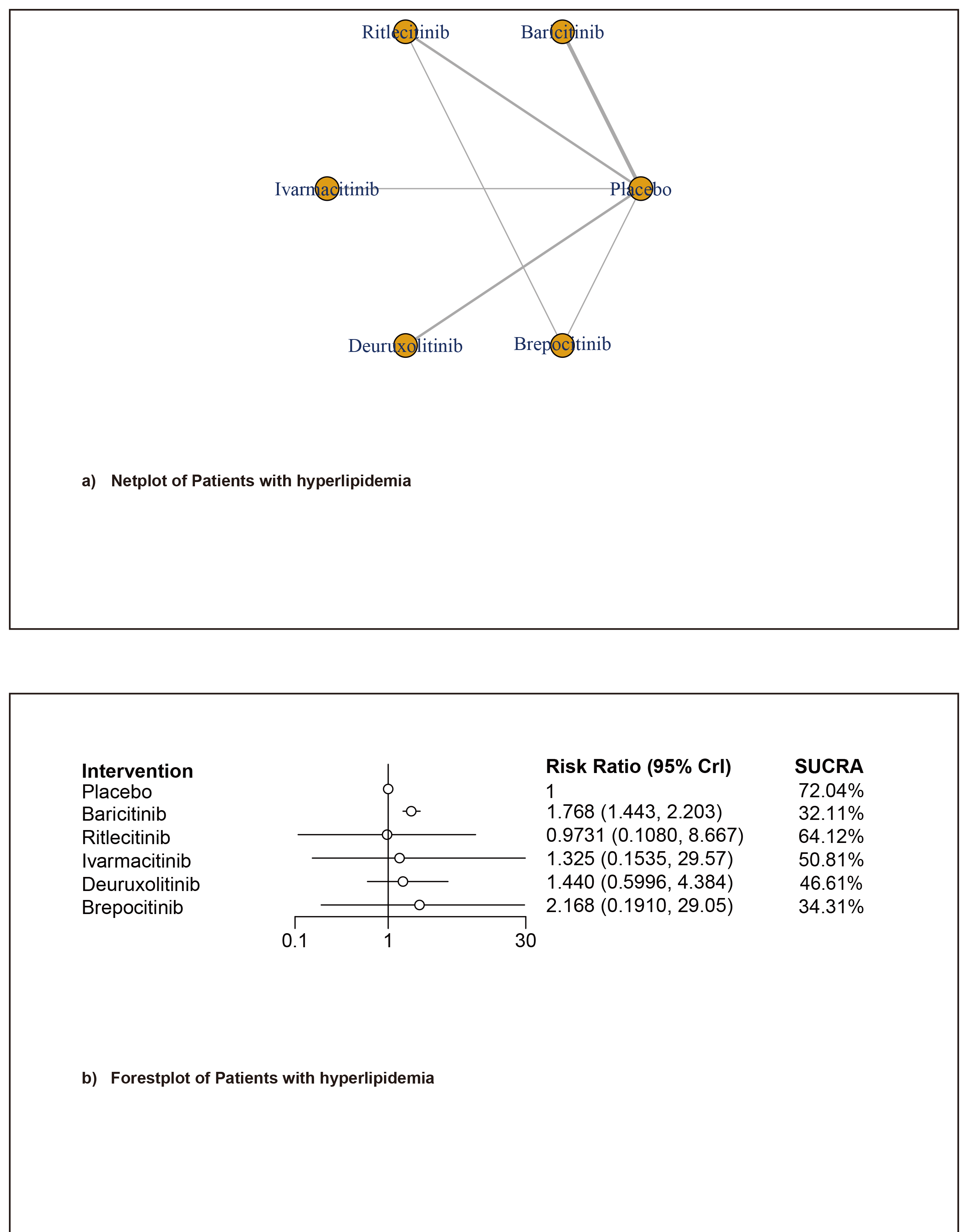
Figure S37.** Netplot and forestplot of patients suffering from hyperlipidemia

**
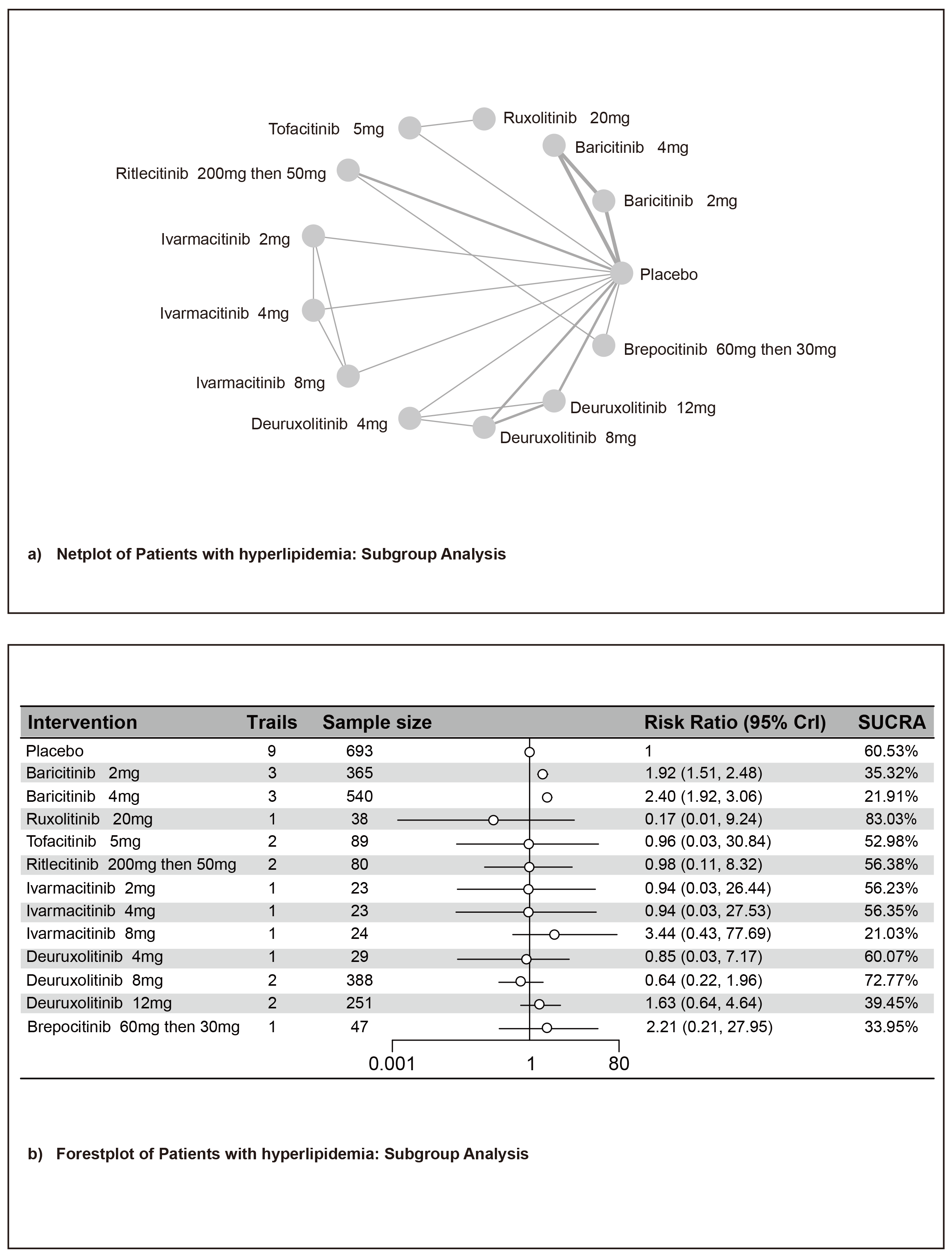
Figure S38.** Netplot and forestplot of patients suffering from hyperlipidemia

**
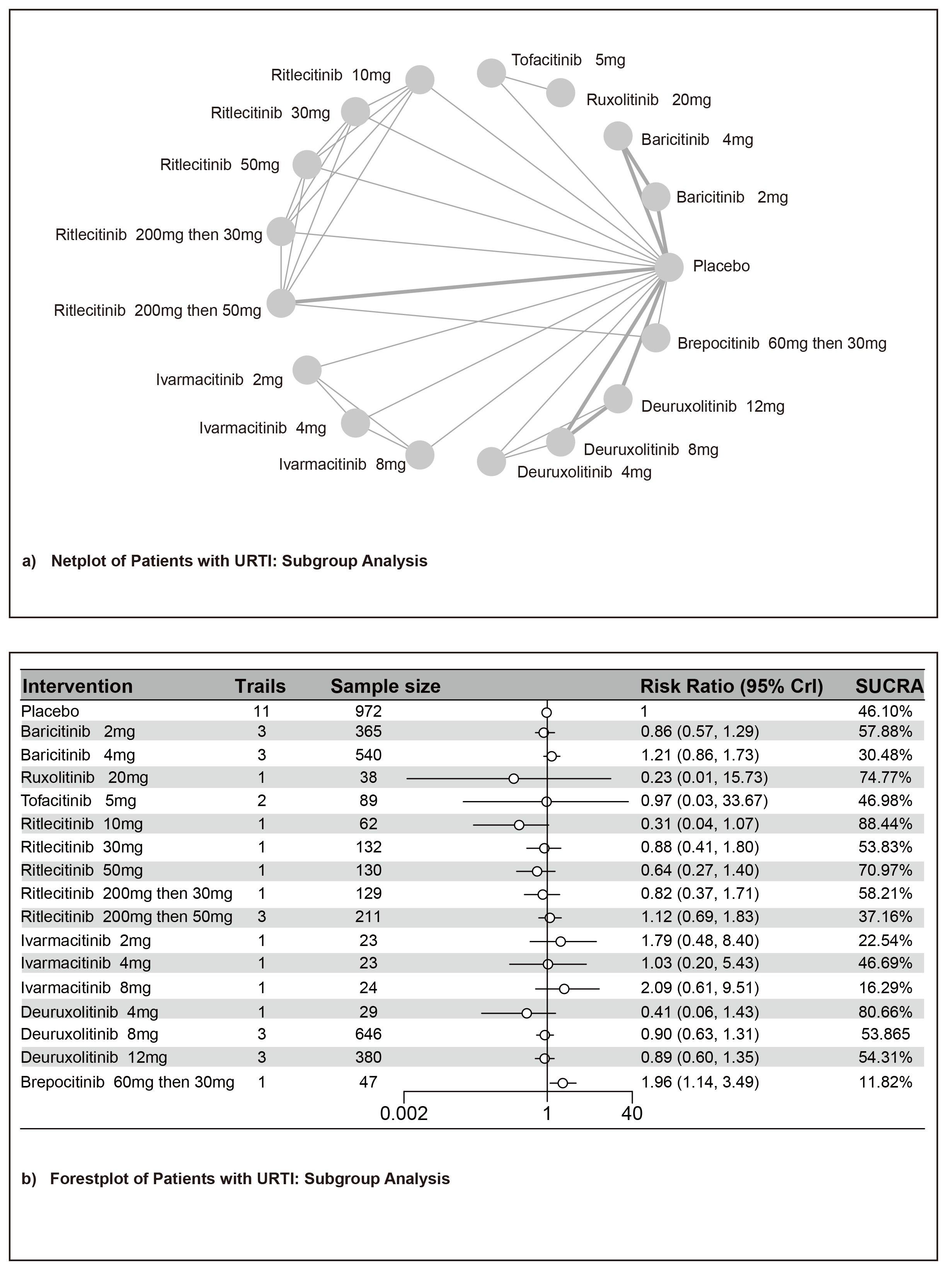
Figure S39.** Netplot and forestplot of patients suffering from URTI (Subgroup Analysis)

**
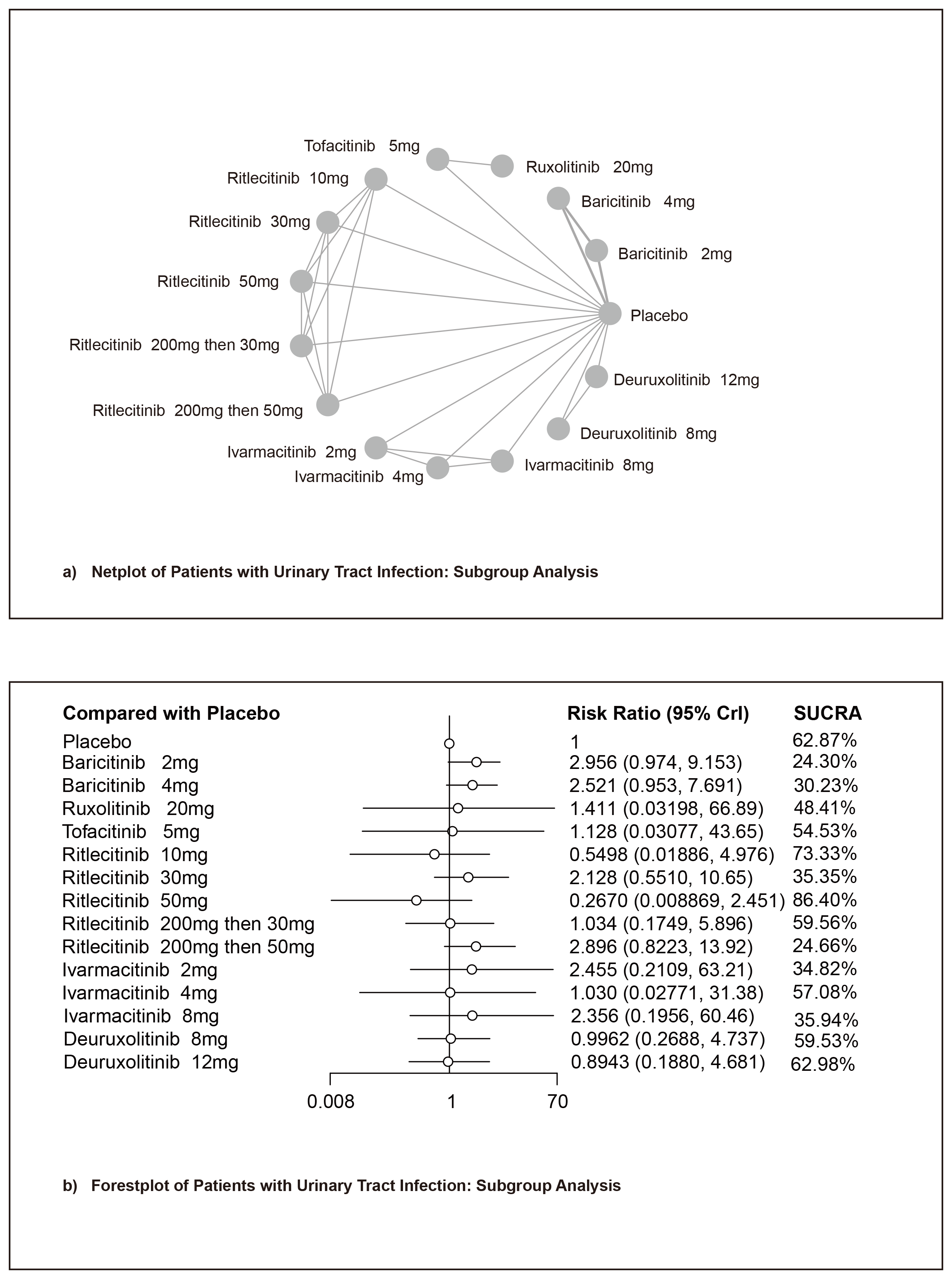
Figure S40.** Netplot and forestplot of patients suffering from Urinary Tract Infection (Subgroup Analysis)

**
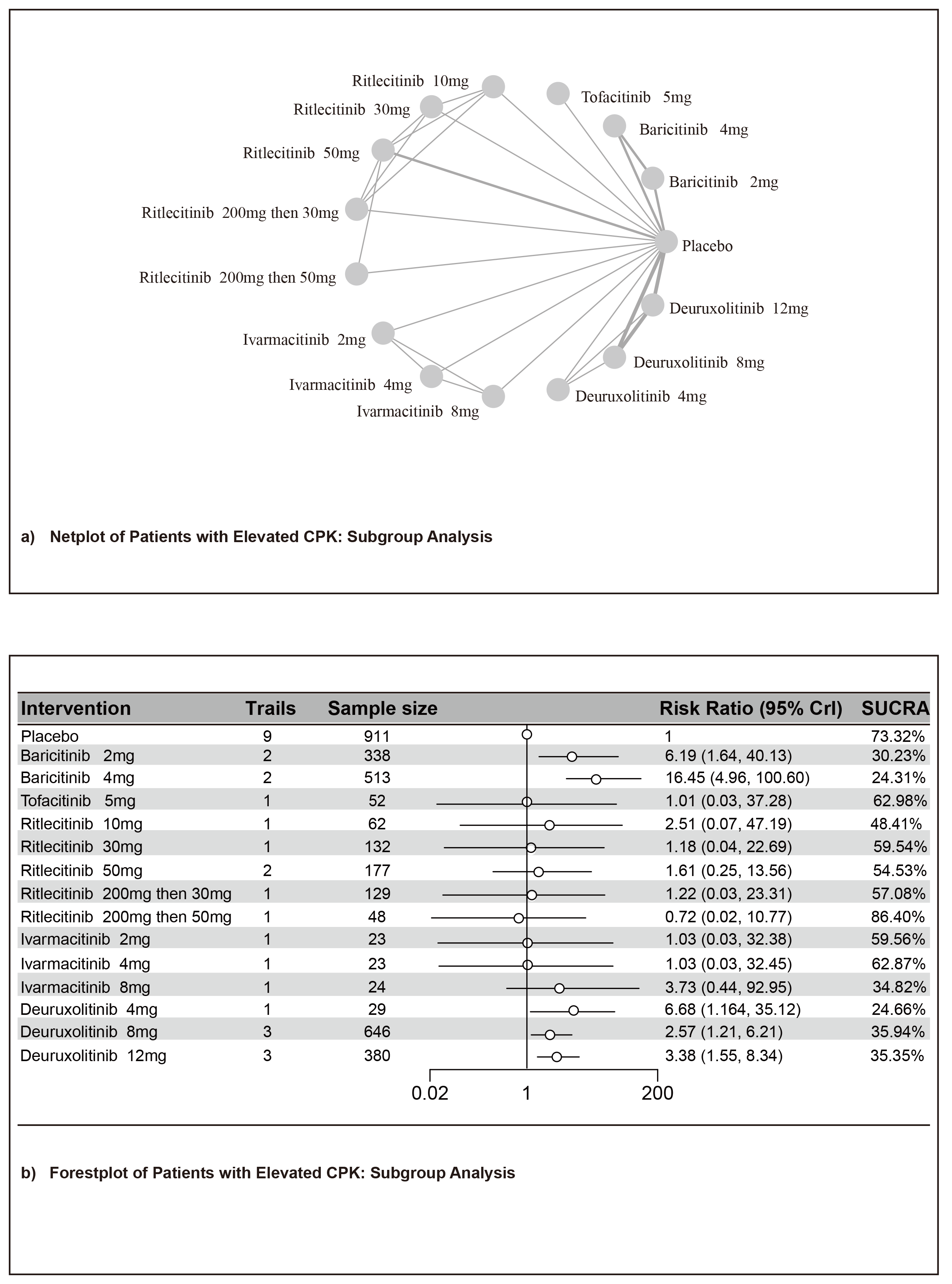
Figure S41.** Netplot and forestplot of patients suffering from CPK (Subgroup Analysis)

**
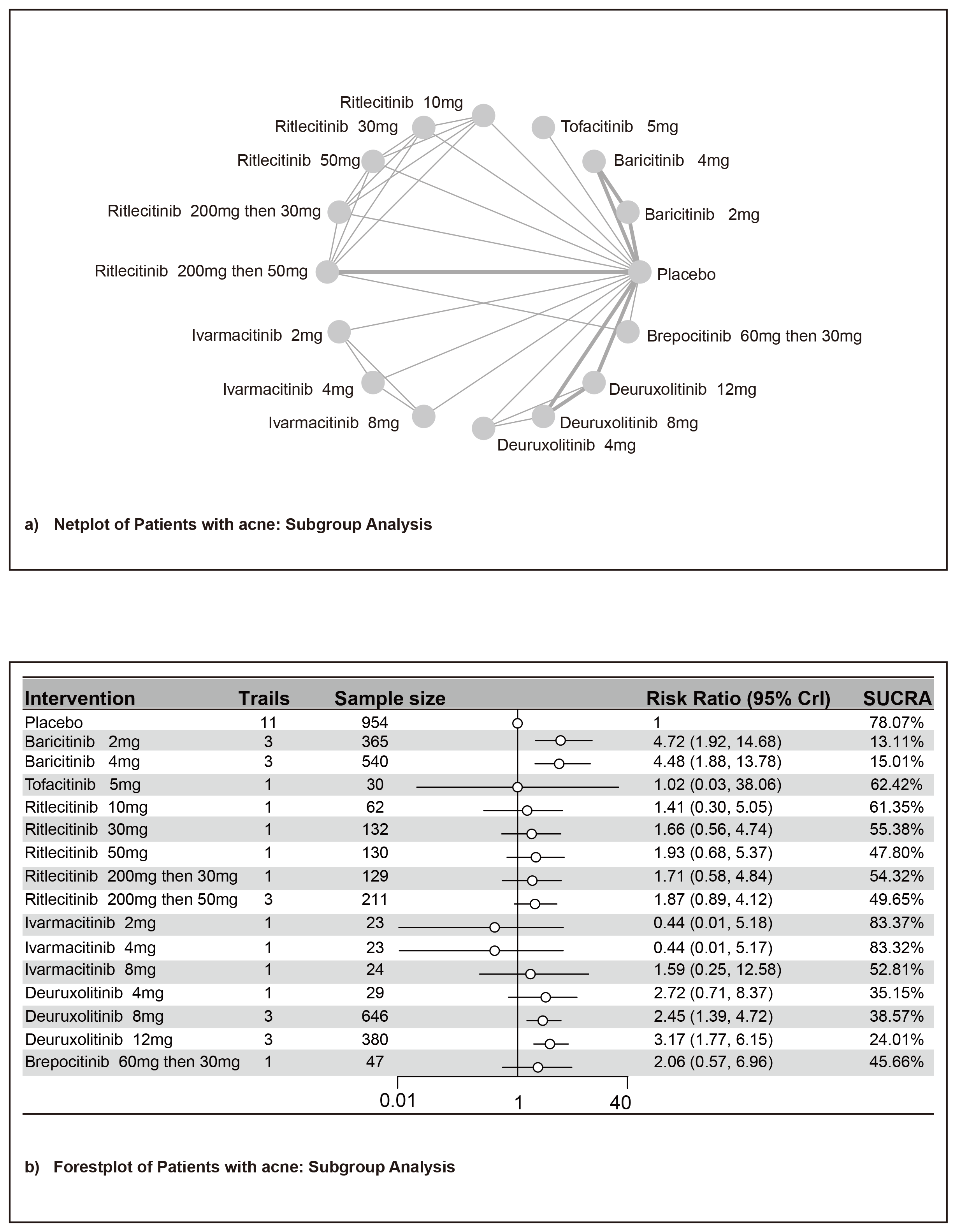
Figure S42.** Netplot and forestplot of patients suffering from Ance (Subgroup Analysis)

**
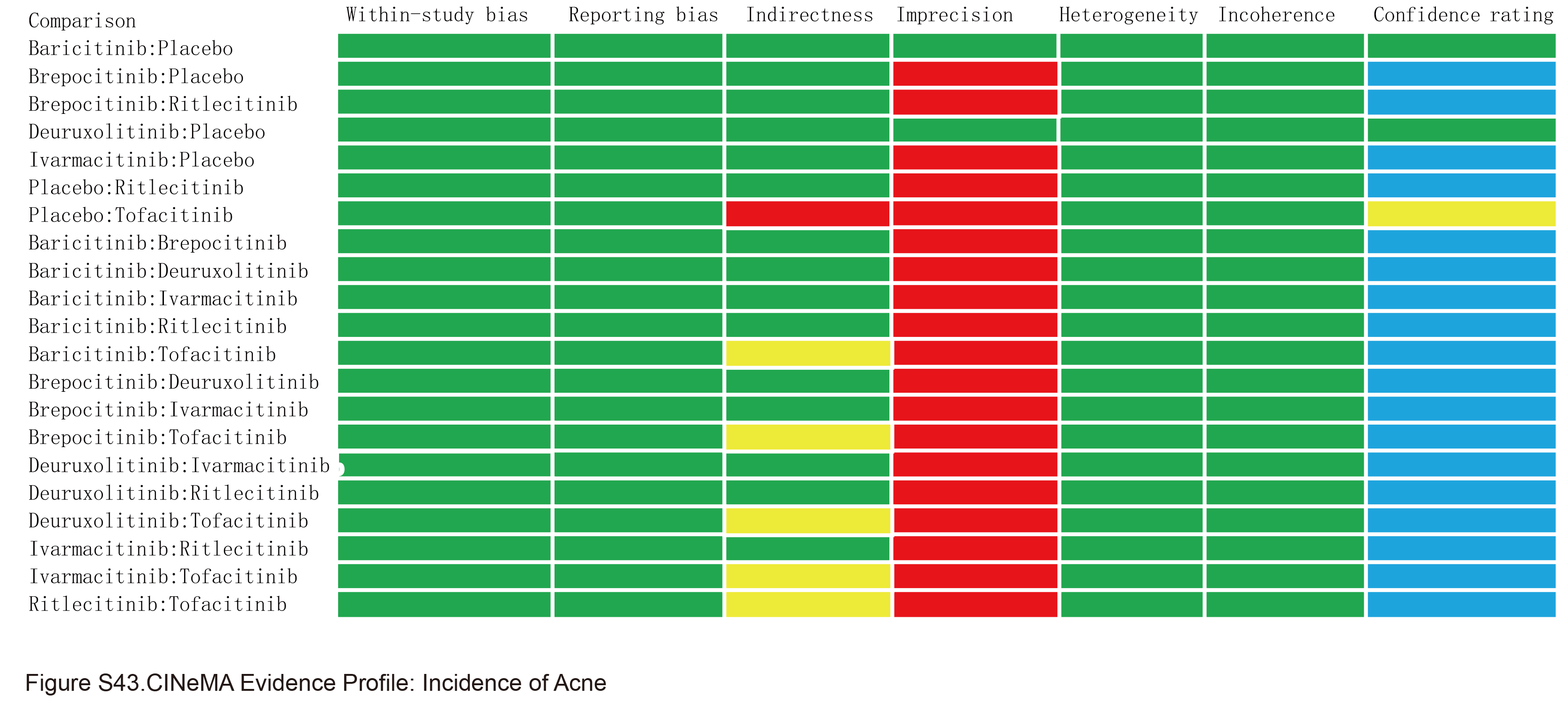
Figure S43.** CINeMA Evidence Profile (Incidence of Acne)

**
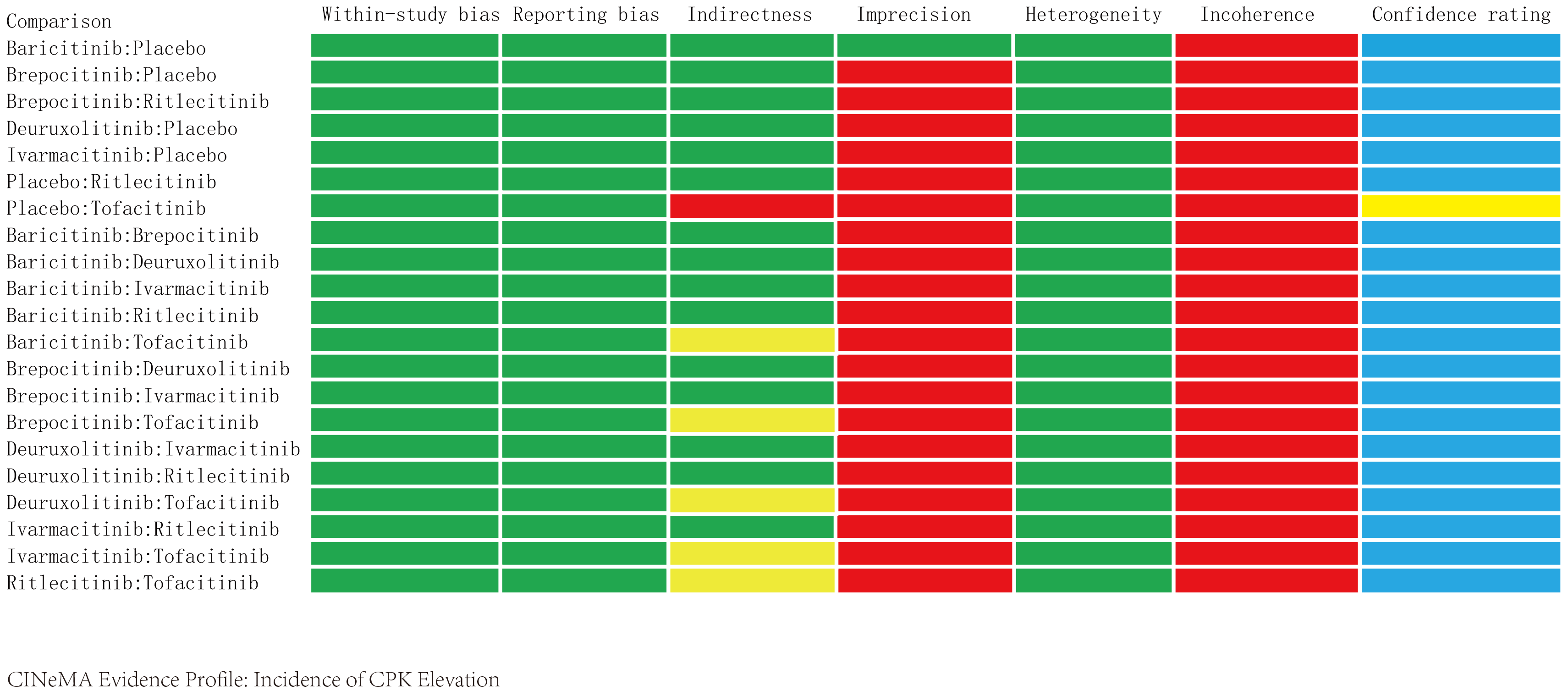
Figure S44.** CINeMA Evidence Profile (Incidence of CPK)

**
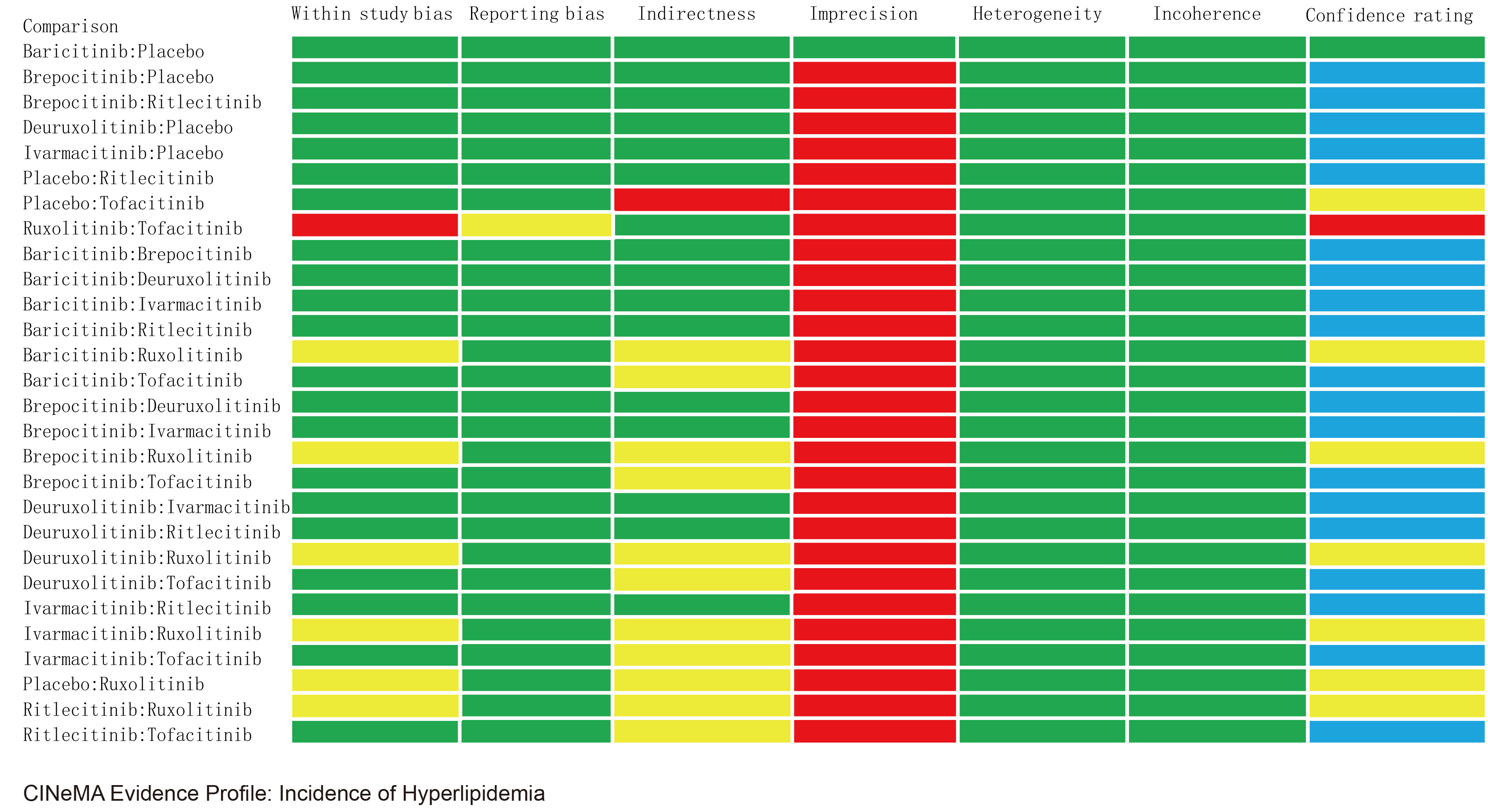
Figure S45.** CINeMA Evidence Profile (Incidence of Hyperlipidemia)

**
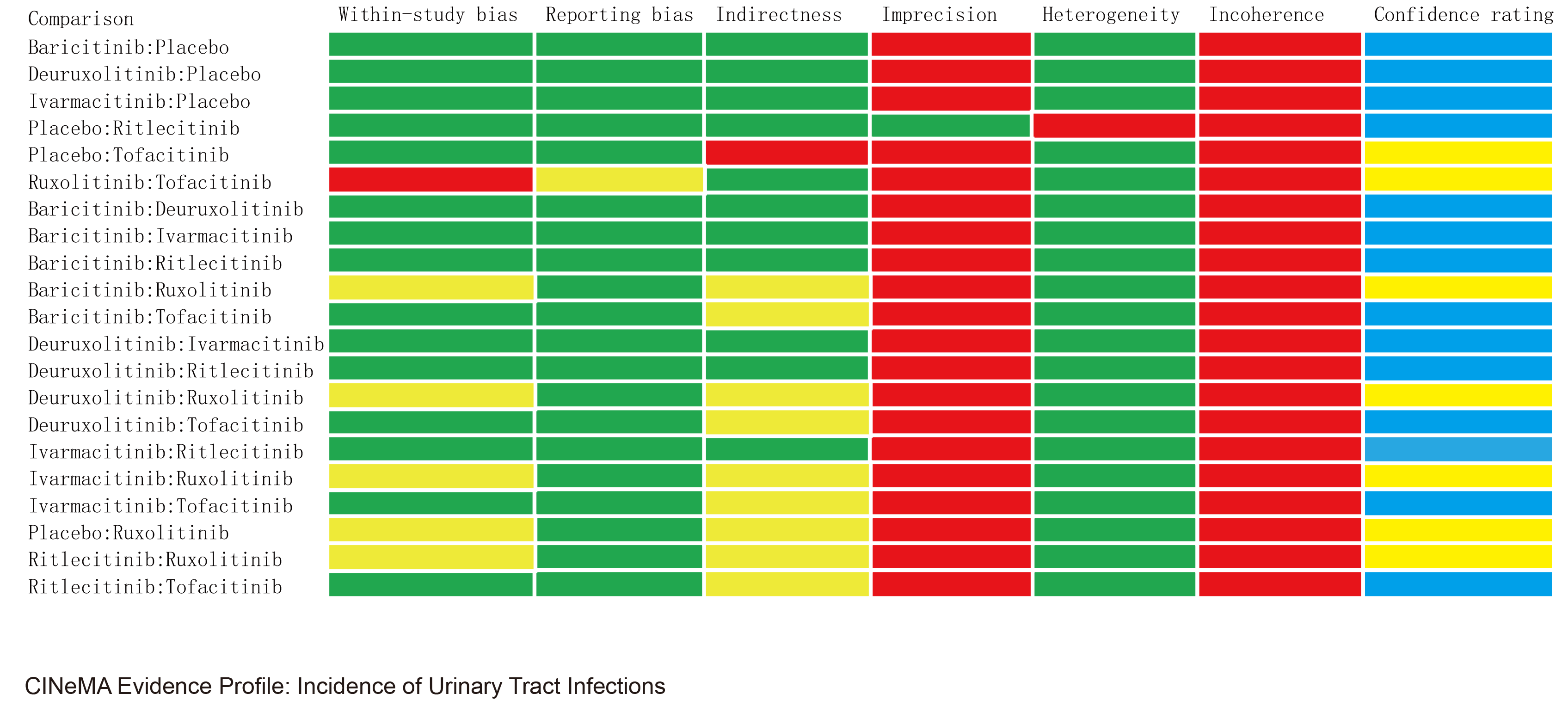
Figure S46.** CINeMA Evidence Profile (Incidence of Urinary Tract Infections)

**
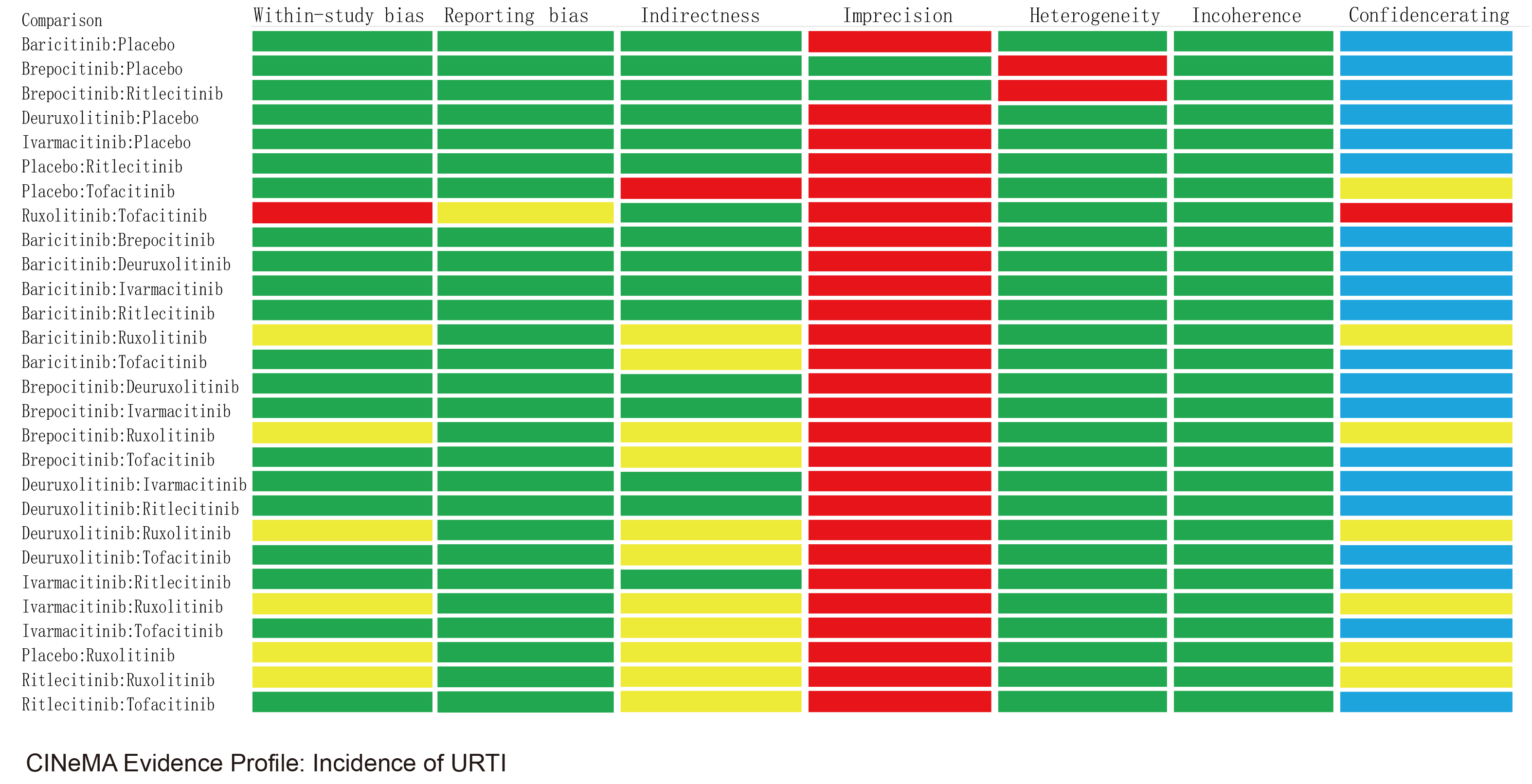
Figure S47.** CINeMA Evidence Profile (Incidence of URTI)
